# Temporal trends in leading causes of injury deaths in Canada from 2000 to 2023: a time series analysis of mortality data

**DOI:** 10.1186/s40621-026-00702-4

**Published:** 2026-08-06

**Authors:** Xiaoquan Yao, Steven R. McFaull, Gisèle Contreras, Wendy Thompson

**Affiliations:** Centre for Surveillance and Applied Research, Health Promotion and Chronic Disease Prevention Branch, Public Health Agency of Canada, 785 Carling Avenue, Ottawa, ON K1A 0K9 Canada

**Keywords:** Injuries, Mortality, Unintentional injuries, Suicide, Poisonings, Falls, Land transport incidents, Leading causes, Trend analysis, Canada

## Abstract

**Background:**

Understanding long-term trends in causes of injury mortality is essential for informing prevention strategies and public health priorities. In Canada, most studies focused on individual causes of injury deaths, with limited examination of how leading causes evolved collectively over time. This study aimed to assess trends in leading causes of injury mortality and changes in the rankings of leading causes in Canada from 2000 to 2023, overall and by sex and age group.

**Methods:**

Mortality data were obtained from the Canadian Vital Statistics - Death database, Canadian Coroner and Medical Examiner Database and provincial/territorial chief coroner or chief medical examiner offices. Leading causes of injury deaths were ranked based on their annual crude mortality rates. A joinpoint regression model was applied to quantify trends by annual percent change of age-standardized or crude mortality rates for each leading cause with stratification by sex and age group.

**Results:**

Falls, poisonings, suicide and land transport incidents were consistently the top four leading causes of injury deaths for both males and females during the study period, although their relative rankings and trends varied by sex. Age groups had different leading cause rankings and trends. Overall, age-standardized mortality rate (ASMR) increased annually for poisonings (9.2%) and falls (4.6%), while ASMR for land transport incidents had an annual decline of 3.1%. ASMR also decreased annually for struck by/against (2.1%), smoke/fire/flame (2.0%), homicide (1.7%) and drowning (0.8%). Compared with males, females experienced a faster increase in fall-related mortality and a steeper decline in land transport incident-related deaths. Females had an annual increase of 0.8% in the suicide ASMR. Crude mortality rate for poisonings increased annually among 10–79-years-olds (6.5%–9.3%), while fall-related mortality increased among those 45 years and older (1.7%–5.7% annually) but declined among those under 45 years (1.1%–4.8% annually).

**Conclusions:**

The profile of injury mortality in Canada has shifted over the past two decades, highlighting the need for targeted injury prevention strategies that address the growing burden of falls and poisonings while sustaining progress in declining causes. Continuous data collection and research are important for injury surveillance and prevention.

**Supplementary Information:**

The online version contains supplementary material available at https://doi.org/10.1186/s40621-026-00702-4.

## Background

Injuries are a significant public health concern, contributing to substantial morbidity and mortality worldwide [1]. In Canada, injuries were associated with an economic burden of at least $29.4 billion in 2018, of which $20.4 billion were from health care expenditures and $8.9 billion were due to reduced productivity from hospitalization, disability and premature death [2]. Most injuries are preventable. Understanding injury causes and mortality trends provide valuable information for injury prevention.

Injuries are caused by diverse mechanisms (such as a fall, poisoning, land transport incident, etc.) [3] and can be sustained unintentionally or with intent by oneself or others (such as suicide or homicide) [4]. Based on a list of 113 selected causes of death [5] developed by the U.S. National Center for Health Statistics [6], unintentional injuries have been ranked among the top five leading causes of all deaths in Canada since 2000 [7]. In 2015, unintentional injuries accounted for 68.1% of total injury deaths [4]. Among the deaths from unintentional injuries, the most common causes were falls, poisonings, motor vehicle traffic crashes (MVT, a main component of land transport incidents), suffocation, drowning, smoke/fire/flames, struck by/against [4]. For simplicity, the qualifier “unintentional” will be omitted hereafter when referring to these causes. Other than unintentional injury deaths, suicide and homicide resulted in 25.4% and 2.6% of the total injury deaths, respectively [4].

Previous studies have shown that injury mortality from some of the common causes in Canada has changed over time [8–11]. The number of deaths due to drug poisonings (a primary contributor of poisonings) has steadily risen since 2000 [8]. The deaths due to falls among older people aged 65 years and older increased in both number and rate from 2010 to 2022 [9]. The mortality rate due to MVT dropped between 2007 and 2012 [10]. The age-standardized suicide rate for individuals aged 10 years and older decreased from 1981 to 2008 but remained stable from 2008 to 2018 [11]. However, these studies either focused on specific causes or populations [8, 9, 11], or used data from a decade ago [10]. Another study [4] observed that, in 2015, poisonings became the 2nd leading cause of unintentional injury deaths, replacing MVT from the previous years, but the study did not conduct a long-term trend analysis. Few studies have examined trends of the leading causes of injury deaths collectively over time with more recent data. Such analyses are critical for identifying shifts in Canada’s injury burden and for informing prevention priorities and evaluating the impact of past interventions. This study aimed to examine the temporal trends in leading causes of injury deaths in Canada from 2000 to 2023, focusing on changes in cause ranking and quantifying cause-specific trends, overall and by sex and age group.

## Methods

### Design

A time series design was used to analyze the temporal trends in the leading causes of injury deaths in Canada from 2000 to 2023. Specifically, it compared annual crude mortality rates from each cause to identify changes in ranking over time. Annual age-standardized mortality rates (ASMRs) were also computed. A joinpoint regression analysis was applied to quantify the trend over time for each cause. The Reporting of Studies Conducted using Observational Routinely-Collected Health Data (RECORD) guideline was followed to report the study [12].

### Setting

In Canada, deaths are registered in the provincial and territorial (P/T) vital statistics registries. Statistics Canada manages the Canadian Vital Statistics - Death database (CVSD) by collecting the death information from P/T vital statistics registries [13]. Although widely used, CVSD is subject to reporting delays, particularly for deaths requiring lengthy coroner or medical examiner investigations [14]. This lag can lead to an underestimation of certain injury deaths at the time of its release, affecting rate estimates and the ranking of leading causes of injury deaths. To improve the timeliness of suicide mortality data for national surveillance, the Public Health Agency of Canada collaborates with Statistics Canada and the P/T chief coroner or chief medical examiner offices to obtain more current data through the Canadian Coroner and Medical Examiner Database (CCMED) [15, 16].

### Data sources and study population

This study included all injury deaths occurring in Canada from three data sources, (1) CVSD [13, 14] for all injury deaths in 2000–2023 except suicide during 2020–2023; (2) suicide 2020–2023 data directly from the P/T’s chief coroner or chief medical examiner offices in British Columbia, Alberta, Manitoba and Nunavut [16]; and (3) suicide 2020–2023 data from CCMED for the other P/Ts [15]. Since 2000, CVSD has been coding the underlying cause of injury deaths based on the external causes from the International Statistical Classification of Diseases and Related Health Problems 10th Revision (ICD-10) [17].

### Outcomes and variables

The following most common causes of injury deaths were examined: falls, poisonings, land transport incidents, suffocation, drowning, smoke/fire/flames, struck by/against, suicide and homicide [4]. Table 1 lists the ICD-10 codes used to define the causes. For contextual understanding, we also analyzed the injury deaths from all causes, all unintentional causes, unintentional causes other than the most common ones, and undetermined causes. To understand the main drivers of changes in top leading causes, we further analyzed sub-causes (ICD-10 in Table 1).

**Table 1:** ICD-10 for injury death causes and sub-causes

Cause	ICD-10
All causes^i^	V01–Y34, Y85–Y87, Y899
All unintentional causes	V01–X59, Y85, Y86
*Falls*	W00–W19 (slip/trip: W01 same level, involving snow/ice/skates/skis/another person: W00, W02–W04 other falls on the same level: W18 involving wheelchair/furniture: W05–W08 on/from stairs: W10 other falls from one level to another: W09, W11–W17 unspecified falls: W19)
*Poisonings*	X40–X49 (psychoactive drugs: X41, X42 nonopioid pain/fever & autonomic drugs: X40, X43 other/unspecified drugs: X44 alcohol: X45 chemicals & gases: X46–X49)
*Land transport incidents*	V01–V89 (MVT: V02–V04(0.1), V02–V04(0.9), V09.2, V12–V14 (0.3-0.9), V19 (0.4-0.6), V20–V28 (0.3-0.9), V29 (0.4-0.9), V30–V79 (0.4-0.9), V80 (0.3-0.5), V81.1, V82.1, V83–V86(0.0-0.3), V87 (0.0-0.8), V89.2 non MVT: codes in the range of V01–V89 excluding MVT)
*Suffocation*	W75–W84
*Drowning*	W65–W74, V90, V92
*Smoke/fire/flames*	X00–X09
*Struck by/against*	W20–W22, W50–W52
*Other unintentional causes*	codes in the range of V01–X59, Y85, Y86 excluding falls, poisonings, Land transport incidents, suffocation, drowning, smoke/fire/flames, struck by/against)
Suicide	X60–X84, Y870 (asphyxia: X70 drugs: X60-X64 firearms: X72-X74 jumping: X80 other means/sequelae: X65-X69, X71, X75-X79, X81-X84, Y870)
Homicide	X85–Y09, Y871
Undetermined causes	Y10–Y34, Y872, Y899

^i^ All causes exclude complications of medical and surgical care, and legal intervention and operations of war

Changes in the rankings of leading causes of injury deaths were assessed using annual crude mortality rates from 2000 to 2023. The temporal trend of each cause was quantified using annual percent change (APC) in mortality rates. Analyses were stratified by sex and age group (0–9, 10–19, 20–44, 45–64, 65–79 and 80+). For total population and sex stratification, ASMRs that account for age structure were used to calculate APC; while for age groups, crude mortality rates were used. For the sub-cause analysis, annual number of deaths and crude mortality rates were computed among the total population.

### Statistical analysis

Statistics Canada’s population data [18] were used to calculate crude and sex- and age-specific mortality rates. All rates are expressed in the unit of per 100 000 population. Statistical uncertainty was assessed using 95% confidence intervals (CI). Direct age standardization was performed based on the 2021 Canadian population, with a 5-year interval (0–4, 5–9, …, 90+) for age stratification for each cause except suicide. Due to data confidentiality, the P/T chief coroner or chief medical examiner offices did not provide suicide data with 5-year intervals, so a different stratification (0–9, 10–14, 15–19, 20–24, 25–44, 45–64, 65–79, 80+) was used for suicide. To assess the impact of the wider age interval, the ASMRs from suicide based on a 5-year interval were compared with those based on the wider age interval for the years 2000 to 2019. A Gamma distribution was applied to get the CIs for the ASMRs [19].

For the suicide 2020–2023 data, sex and/or age information was missing for few deaths (missing sex: 2 deaths in 2021, 7 deaths in 2022, 16 deaths in 2023; missing age: 2 deaths in 2021, 2 deaths in 2022, 1 death in 2023). Those deaths were included in the total population-analyses but excluded for sex- or age- analyses. In addition, sub-cause information was missing for the suicide 2020–2023.

A joinpoint regression model was applied to quantify temporal trends. The model uses segmented linear regression [20], which finds the optimal number and locations of join points (also known as change points) that best fit the data. Through the regression coefficient (β) of log of mortality rate on year, APC was calculated using the formula.

APC = [exp(β) – 1] × 100.

The AAPC (average annual percent change) from 2000 to 2023 was computed by the weighted average of the APCs, with the weights equal to the length of the segments (i.e., the number of years) [20]. If a mortality rate was zero (zero count) for a year, a method of combining years was used, for example, rates were calculated for 2000–2001, 2002–2003,…, 2022–2023.

To assess the robustness of the joinpoint regression findings, a sensitivity analysis was conducted by excluding data in 2023, when the death records with unknown causes in CVSD are relatively high compared to the previous years at the time of analysis. The joinpoint model was re-estimated using data from 2000 to 2022. This approach was used to determine whether the inclusion of potentially incomplete or unstable 2023 data substantially influenced the model estimates.

SAS EG 7.1 was used to analyse data. Further, the Joinpoint 5.4.0.0 software [21] was utilized to perform the joinpoint regression analysis. We followed the standard Joinpoint practice, assuming errors to be heteroscedastic but uncorrelated across years [21]. The model selection method was Weighted Bayesian Information Criteria. The maximum number of join points was set to 4; the minimum number of observations before the first join point, after the last join point and between two join points was set to 2. P value was set at 0.05.

### Patient and public involvement

Patient involvement was not applicable. Parachute, Canada’s national injury prevention charity and publisher of the Cost of Injury in Canada Reports [2, 22], was consulted during the reporting of this study.

### Ethics approval

This study did not require research ethics review pursuant to article 2.2(a) of the Canadian Tri-Council Policy Statement: Ethical Conduct for Research Involving Humans because the human data used was publicly available through a mechanism set out by legislation or regulation and protected by law.

## Results

### Ranking change in leading causes

**Fig. 1:**
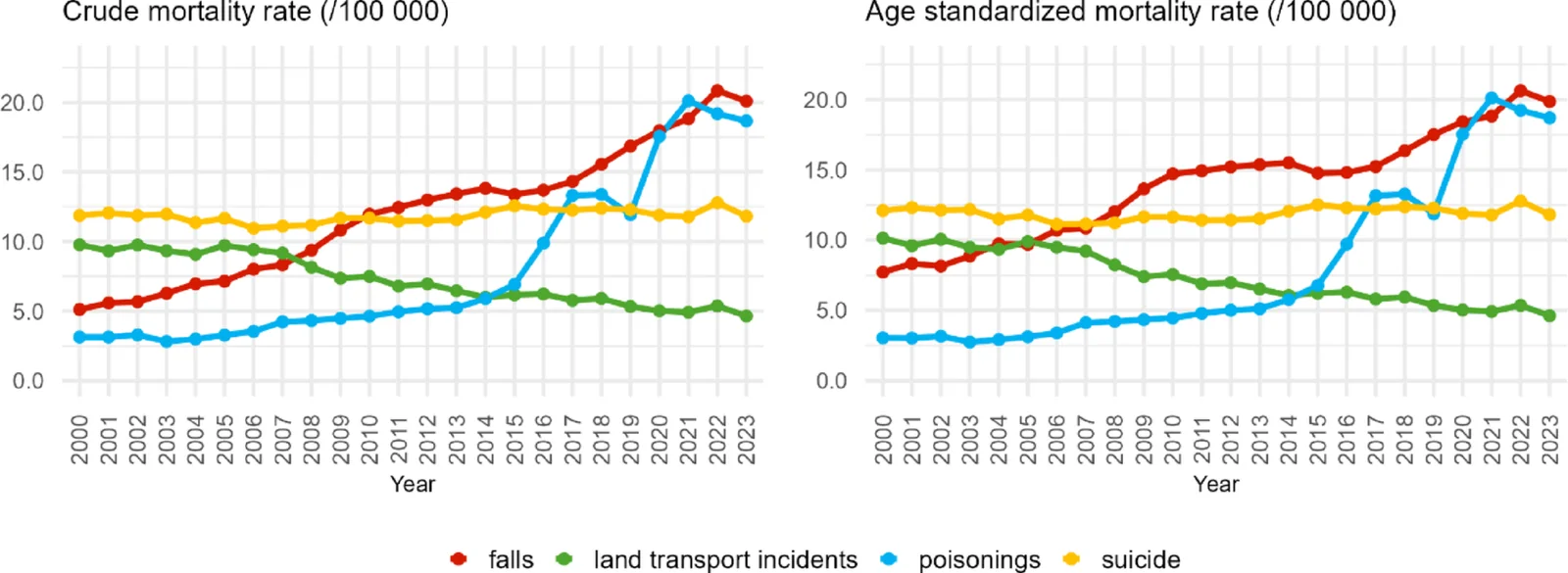
Crude mortality rates and age standardized mortality rates from leading causes of injury deaths, total population, 2000–2023

The additional file 1 provides the numbers of deaths, crude mortality rates, ASMRs and CIs from 2000 to 2023 for each cause of injury deaths (Table A1-1 – A9-4) and sub-causes (Table 10-1–13-2). Based on the crude mortality rates, falls, poisonings, land transport incidents and suicide were the top four leading causes of injury deaths throughout the study period for the total population (Table A1-3). Figure 1 shows the crude mortality rates from these four causes by year. Their ranking changed during the study period. In 2000, suicide was the leading cause of death (11.9), followed by land transport incidents (9.8), falls (5.1) and poisonings (3.1); in 2023, falls ranked first (20.1), followed by poisonings (18.7), suicide (11.8) and land transport incidents (4.6). Specifically, falls have been the leading cause of injury deaths since 2010 (except in 2021 when poisonings were the leading cause). Figure 1 also shows the ASMRs from the top four leading causes by year, presenting similar trends in the ASMRs as in the crude rates.

**Fig. 2:**
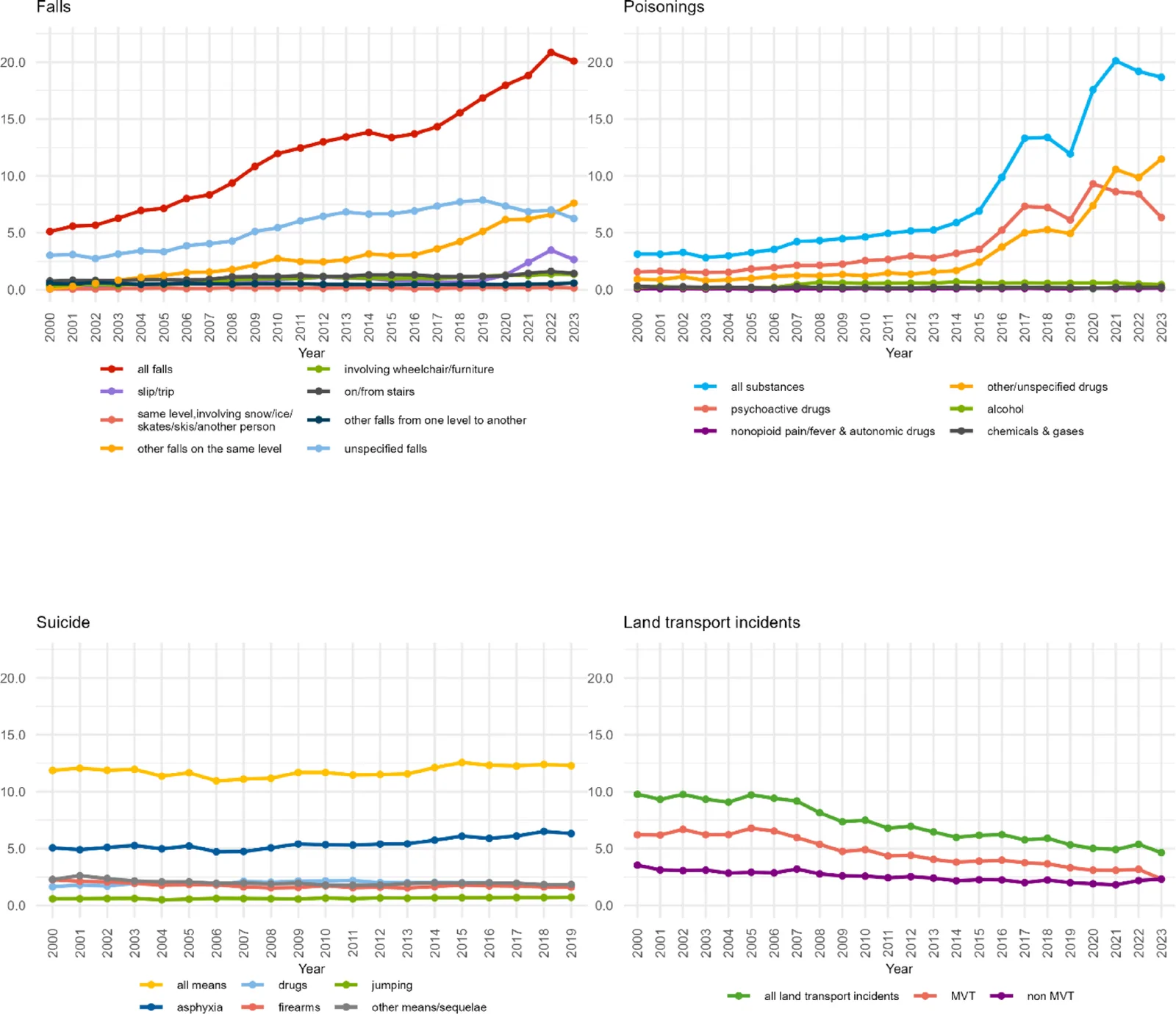
Crude mortality rates by sub-cause for the top leading causes, total population, 2000–2023*. * For suicide, the period covered only 2000–2019 because the 2020–2023 data did not have the sub-cause information; MVT — motor vehicle trafficcrashes

Figure 2 displays the trends in crude mortality rates by sub-cause for the top four leading causes among the total population. Among poisoning deaths, psychoactive drugs and other/unspecified drugs (coded neither in psychoactive drugs nor nonopioid pain/fever & autonomic drugs) accounted for the highest mortality rates and largely drove the observed increases over time. For falls, unspecified falls and other falls on the same level (coded neither in slip/trip nor involving snow/ice/skates/skis/another person) contributed the greatest mortality increase. From 2019 to 2023, slip/trip-related falls increased, whereas unspecified falls decreased. Asphyxia (hanging, strangulation and suffocation) consistently represented the leading mechanism of suicide with an increase during the 2014–2019 period. The decline in mortality from land transport incidents was primarily due to reductions in MVT deaths.

**Fig. 3:**
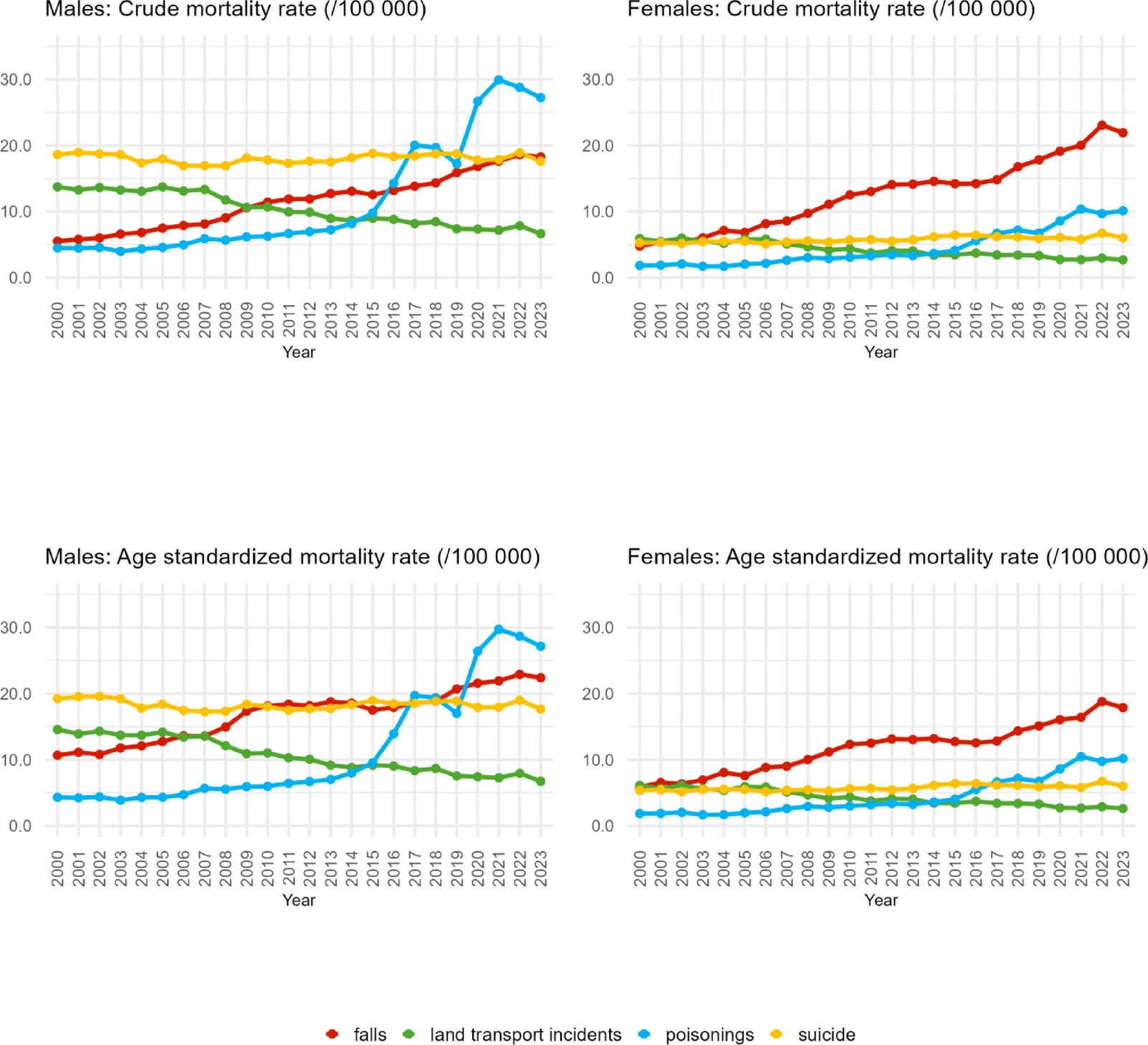
Crude mortality rates and age standardized mortality rates from leading causes of injury deaths by sex, 2000–2023.

For both males and females, falls, poisonings, land transport incidents and suicide were the top four leading causes of injury deaths from 2000 to 2023 (additional file 1: Table A2-1 – A3-6). Figure 3 shows the sex-specific crude mortality rates from the four leading causes by year. Overall, crude mortality rates were higher among males than females throughout the study period for all causes except falls. Among males, suicide was the leading cause of injury death from 2000 to 2016, after which poisonings became the leading cause (except in 2019). Among females, falls were the leading cause from 2003 onward; by 2023, the fall-related mortality rate was more than double that of poisonings (21.9 vs. 10.1). Since 2006, fall-related crude mortality rates have been consistently higher among females than males.

Figure 3 also shows the sex-specific ASMRs from the four leading causes of injury deaths by year. For males, the ASMRs from falls were higher than from suicide in some years. Although crude fall-related mortality rates were higher among females since 2006, the ASMRs for falls were consistently higher among males throughout 2000–2023, reflecting differences in age structure between sexes.

**Fig. 4:**
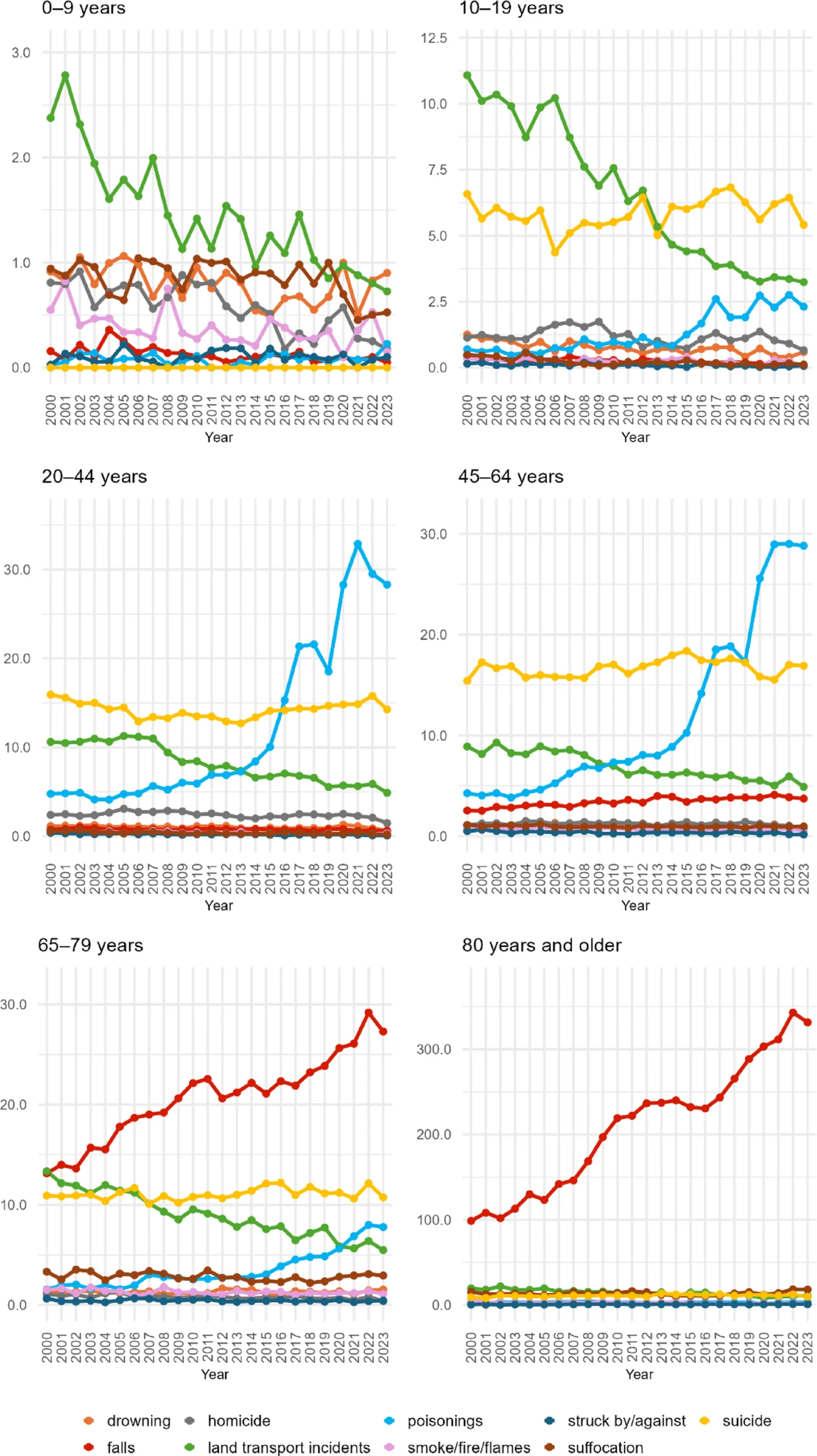
Age-specific crude mortality rates (/100 000)* from leading causes of injury deaths, 2000–2023. * For age group 0–9 years, suicide rates were set to 0 as a small number suppressing method

The leading causes of injury deaths varied among different age groups based on age-specific crude mortality rates (Fig. 4, additional file Table A4-1 – A9-4). Among young children aged 0–9 years, land transport incidents were a leading cause, while suffocation or drowning ranked first in some years. Suicide rates were set to zero in Fig. 4 for this age group as a data suppressing method. For children and youth aged 10–19 years, land transport incidents ranked as the leading cause of injury deaths from 2000 to 2013 and second from 2014 to 2023, while suicide rose from the second to first over the same periods. Poisonings has been the third leading cause of injury deaths for 10–19-year-olds beginning in 2014 to the end of the study period, while homicide consistently ranked third or fourth. For adults aged 20–44 and 45–64 years, similar patterns were observed, with poisonings, suicide, and land transport incidents as the top three causes, though with different transition points. Poisonings became the leading cause by 2016–2017, accompanied by a marked increase in mortality rates. Homicide ranked fourth among those aged 20–44 years, whereas falls ranked fourth among those aged 45–64 years. Among adults aged 65–79 years, falls have been the leading cause since 2001, with steadily increasing mortality rates; suicide has remained the second leading cause since 2005. Among those aged 80 years and older, falls were the leading cause of injury deaths throughout the study period, with sharply rising mortality rates and a widening gap relative to other causes. The mortality rate from falls increased from 98.6 in 2000—five times higher than the second leading cause (land transport incidents: 19.6)—to 331.5 in 2023, nearly 18 times higher than the second leading cause (suffocation: 18.0).

### Annual percent changes

Table 2 presents the AAPC and APCs of the ASMRs from each cause for total population and by sex. For the total population, the ASMR due to falls increased 4.6% annually for the study period. The period 2000–2011 saw the steepest increase (APC: 6.8%). The ASMR from poisonings had an annual increase of 9.2% from 2000 to 2023. For land transport incidents, the ASMR steadily decreased (AAPC: -3.1). The significant decline started in 2006. Overall, there was no significant change in ASMR for suicide during the study period. However, the ASMR for suicide did show significant increase during 2006–2023 (APC: 0.5). ASMR also decreased annually for struck by/against (2.1%), smoke/fire/flame (2.0%), homicide (1.7%), and drowning (0.8%).

Sex-specific comparisons showed that ASMRs for falls increased more rapidly among females than males, while ASMRs for land transport incidents declined more steeply among females (AAPC for falls: 5.3% vs. 3.4%; AAPC for land transport incidents: −3.8% vs. −3.1%). In contrast, suicide ASMRs among females showed a small but statistically significant annual increase (AAPC: 0.8%).

**Table 2:** AAPC and APC of ASMRs by cause, total population and by sex, 2000–2023

Cause	Total population							Males							Females						
	AAPC	LCL^#^	UCL^#^	period	APC	LCL^#^	UCL^#^	AAPC	LCL^#^	UCL^#^	period	APC	LCL^#^	UCL^#^	AAPC	LCL^#^	UCL^#^	period	APC	LCL^#^	UCL^#^
All causes^i^	1.4*	1.2	1.6	2000–2015	0.3	-0.0	0.7	1.1*	0.8	1.4	2000–2014	-0.1	-0.7	0.4	1.9*	1.7	2.1	2000–2015	0.9*	0.5	1.2
				2015–2021	4.8*	3.8	7.7				2014–2021	4.5*	1.5	7.5				2015–2023	3.8*	3.1	4.8
				2021–2023	-0.8	-3.9	2.7				2021–2023	-1.8	-5.5	2.8							
All unintentional causes	1.8*	1.5	2.1	2000–2015	0.3	-0.2	0.8	1.7*	1.3	2.0	2000–2015	0.1	-0.6	0.7	2.1*	1.9	2.3	2000–2015	0.6*	0.1	1.0
				2015–2021	6.7*	5.5	10.5				2015–2021	7.1*	5.7	11.9				2015–2023	4.9*	4.2	6.1
				2021–2023	-0.7	-4.7	3.5				2021–2023	-2.3	-7.0	2.7							
*Falls*	4.6*	4.3	5.2	2000–2011	6.8*	5.9	8.3	3.4*	3.1	3.9	2000–2007	4.0	-0.7	7.4	5.3*	5.0	5.7	2000–2012	7.3*	6.6	8.5
				2011–2016	-0.2	-4.1	2.1				2007–2010	10.3*	1.9	12.6				2012–2016	-1.6	-4.9	1.4
				2016–2023	4.8*	3.5	8.7				2010–2017	-0.3	-2.8	10.5				2016–2023	5.9*	4.8	8.0
											2017–2020	6.4	-0.5	8.1							
											2020–2023	1.5	-1.8	3.9							
*Poisonings*	9.2*	8.2	10.4	2000–2014	5.2*	1.9	7.4	9.5*	8.3	10.8	2000–2014	4.9*	1.0	7.6	8.2*	7.3	9.4	2000–2013	5.8*	1.9	8.7
				2014–2017	32.2*	17.8	40.7				2014–2017	36.5*	20.0	46.6				2013–2021	14.4*	7.8	24.6
				2017–2023	8.4*	2.7	11.2				2017–2023	8.3*	2.3	11.4				2021–2023	-0.2	-8.4	11.2
*Land transport incidents*	-3.1*	-3.4	-2.6	2000–2006	-0.5	-1.9	3.3	-3.1*	-3.5	-2.6	2000–2007	-1.0	-2.4	2.3	-3.8*	-4.3	-3.3	2000–2023	-3.8*	-4.3	-3.3
				2006–2009	-7.7*	-9.7	-3.9				2007–2010	-7.7*	-9.8	-3.7							
				2009–2023	-3.2*	-3.9	-0.4				2010–2023	-3.1	-4.0	0.5							
*Suffocation*	-0.2	-0.7	0.7	2000–2012	-0.8	-2.1	8.3	-0.2	-1.3	0.7	2000–2018	-1.6*	-5.9	-0.5	-0.2	-0.6	0.4	2000–2009	0.0	-1.3	5.5
				2012–2015	-6.9	-10.5	3.1				2018–2023	4.9	-0.3	17.3				2009–2016	-3.9*	-9.8	-2.0
				2015–2023	3.3*	0.7	10.6											2016–2023	3.4*	1.5	7.8
*Drowning*	-0.8*	-1.3	-0.4	2000–2023	-0.8*	-1.3	-0.4	-1.2*	-1.9	-0.6	2000–2023	-1.2*	-1.9	-0.6	0.6	-0.1	1.4	2000–2023	0.6	-0.1	1.4
*Smoke/fire/flames*	-2.0*	-2.9	-1.1	2000–2023	-2.0*	-2.9	-1.1	-1.8*	-2.8	-0.8	2000–2023	-1.8*	-2.8	-0.8	-2.3*	-3.4	-1.3	2000–2023	-2.3*	-3.4	-1.3
*Struck by/against*	-2.1*	-2.8	-1.4	2000–2023	-2.1*	-2.8	-1.4	-2.4*	-3.3	-1.5	2000–2023	-2.4*	-3.3	-1.5	-0.5	-4.9	5.4	2000–2014	3.8	-0.3	67.2
																		2014–2023	-6.8*	-41.3	-0.4
*Other unintentional causes*	-2.3*	-3.1	-1.7	2000–2003	0.6	-6.1	10.2	-2.8*	-3.5	-2.1	2000–2012	-5.6*	-8.4	-4.1	-2.4*	-3.0	-1.8	2000–2003	0.8	-6.1	9.3
				2003–2012	-7.3	-13.3	4.5				2012–2023	0.3	-1.1	3.6				2003–2012	-8.5*	-13.7	-6.7
				2012–2023	1.3	-1.7	3.1											2012–2023	2.1*	0.7	3.6
Suicide	0.0	-0.3	0.4	2000–2006	-1.4	-5.6	0.2	-0.3	-0.6	0.1	2000–2006	-1.8*	-6.1	-0.2	0.8*	0.5	1.2	2000–2023	0.8*	0.5	1.2
				2006–2023	0.5*	0.1	2.3				2006–2023	0.3	-0.1	1.8							
Homicide	-1.7*	-2.6	-0.8	2000–2005	4.7*	1.0	13.2	-1.6*	-2.3	-0.9	2000–2008	2.6*	0.9	5.9	-2.8*	-4.9	-0.4	2000–2018	-1.3	-3.0	19.1
				2005–2014	-3.2*	-9.8	-1.9				2008–2014	-5.7*	-12.2	-3.1				2018–2023	-7.8*	-31.2	-1.7
				2014–2020	2.1	-0.4	9.3				2014–2020	4.4*	1.7	11.7							
				2020–2023	-13.8*	-24.4	-7.7				2020–2023	-15.1*	-24.9	-9.0							
Undetermined causes	-1.7*	-3.0	-0.4	2000–2014	2.9*	1.4	5.2	-1.6*	-2.7	-0.3	2000–2014	2.7*	1.1	4.9	-0.3	-2.0	1.8	2000–2003	22.5*	6.0	63.3
				2014–2017	-30.0*	-36.8	-16.6				2014–2017	-29.5*	-35.9	-16.4				2003–2014	1.5	-2.6	3.5
				2017–2023	4.6	-2.1	27.0				2017–2023	5.3	-0.8	21.6				2014–2017	-29.1*	-35.2	-15.3
																		2017–2023	2.9	-3.7	20.3

AAPC: average annual percent change APC: annual percent change ASMRs: age standardized mortality rates

^#^ LCL: 95% lower confidence limit; UCL: 95% upper confidence limit

^i^ All causes exclude medical and surgical adverse effects, and legal intervention and operations of war

* *p*<0.05

Table 3 presents the AAPC and APCs of the age-specific crude mortality rates from each cause. The mortality rates from falls decreased among 0–44-year-olds (AAPC for 0–9 years: -4.8; for 10–19 years: -3.6; for 20–44 years: -1.1) but increased among the older age groups (AAPC for 45–64 years: 1.7; for 65–79 years: 3.4; for 80 + years: 5.7). The mortality rates from poisonings increased annually by 6.5%–9.3% among those aged 10–79 years. For land transport incidents, all age groups showed negative AAPC. There was no significant change for the suicide rate among all age groups.

**Table 3:** AAPC and APC of crude mortality rates by cause and age group, 2000–2023

Cause	0–9 years							10–19 years							20–44 years						
	AAPC	LCL^#^	UCL^#^	period	APC	LCL^#^	UCL^#^	AAPC	LCL^#^	UCL^#^	period	APC	LCL^#^	UCL^#^	AAPC	LCL^#^	UCL^#^	period	APC	LCL^#^	UCL^#^
All causes^i^	-2.8*	-3.5	-2.2	2000–2023	-2.8*	-3.5	-2.2	-2.1*	-2.4	-1.8	2000–2023	-2.1*	-2.4	-1.8	1.2*	0.6	1.6	2000–2014	-0.7	-1.6	0.1
																		2014–2021	7.2*	5.6	13.4
																		2021–2023	-5.6	-12.2	1.6
All unintentional causes	-2.7*	-3.3	-2.3	2000–2023	-2.7*	-3.3	-2.3	-3.3*	-4.2	-2.7	2000–2015	-4.3	-8.6	3.1	2.4*	1.6	3.1	2000–2013	-1.1	-2.8	0.3
											2015–2023	-1.6	-5.3	7.4				2013–2021	10.8*	8.8	19.0
																		2021–2023	-6.5	-15.0	3.6
* Falls*	-4.8*	-7.9	-2.4	2000–2023	-4.8*	-7.9	-2.4	-3.6*	-5.4	-2.2	2000–2023	-3.6*	-5.4	-2.2	-1.1*	-2.0	-0.3	2000–2023	-1.1*	-2.0	-0.3
* Poisonings*	1.4	-3.1	6.5	2000–2023^@^	1.4	-3.1	6.5	6.5*	4.6	8.4	2000–2014	3.9	-10.6	7.8	8.6*	7.4	11.5	2000–2013	3.7	-9.4	40.4
											2014–2017	30.7*	10.4	44.2				2013–2017	29.4	-13.2	44.6
											2017–2023	1.8	-11.2	8.7				2017–2021	13.1*	9.6	39.9
																		2021–2023	-4.6	-16.2	7.9
* Land transport incidents*	-4.7*	-5.8	-3.8	2000–2023	-4.7*	-5.8	-3.8	-5.7*	-7.1	-4.8	2000–2006	-2.4	-5.4	7.9	-2.9*	-3.3	-2.5	2000–2006	1.5*	0.1	4.3
											2006–2023	-6.8*	-12.6	-6.1				2006–2009	-9.2*	-11.2	-4.8
																		2009–2023	-3.4*	-4.0	-1.6
* Suffocation*	-3.2*	-5.5	-1.6	2000–2019	-0.2	-1.3	2.0	-6.1*	-8.3	-4.5	2000–2023	-6.1*	-8.3	-4.5	-3.2*	-4.8	-1.9	2000–2023	-3.2*	-4.8	-1.9
				2019–2023	-16.4*	-39.4	-5.4														
* Drowning*	-1.2	-3.0	0.4	2000–2023	-1.2	-3.0	0.4	-3.3*	-4.7	-2.1	2000–2023	-3.3*	-4.7	-2.1	-2.6*	-4.4	-1.4	2000–2018	-1.5	-7.5	0.9
																		2018–2021	10.6	-0.5	17.3
																		2021–2023	-27.6*	-42.0	-10.1
* Smoke/fire/flames*	-2.8*	-5.3	-0.6	2000–2023	-2.8*	-5.3	-0.6	-2.2	-5.4	0.3	2000–2023	-2.2*	-5.4	0.3	-3.4*	-4.8	-2.2	2000–2023	-3.4*	-4.8	-2.2
* Struck by/against*	-0.5	-3.8	2.9	2000–2012^&^	5.1*	0.6	25.6	-4.1*	-7.8	-1.4	2000–2023	-4.1*	-7.8	-1.4	-3.8*	-5.4	-2.4	2000–2023	-3.8*	-5.4	-2.4
				2012–2023^&^	-6.9*	-23.2	-1.7														
* Other unintentional causes*	-1.5	-3.6	0.5	2000–2023	-1.5	-3.6	0.5	-3.1*	-4.6	-1.8	2000–2023	-3.1*	-4.6	-1.8	-1.7*	-2.5	-1.0	2000–2016	-3.5*	-5.7	-2.5
																		2016–2023	2.5	-0.9	12.9
Suicide^$^	-	-	-	2000–2023	-	-	-	0.1	-0.6	0.9	2000–2006	-2.8	-11.4	0.2	-0.2	-0.5	0.0	2000–2008	-2.5*	-3.9	-1.5
											2006–2023	1.1*	0.4	5.6				2008–2023	1.0	0.6	1.5
Homicide	-4.3*	-6.4	-2.7	2000–2023	-4.3*	-6.4	-2.7	-1.9*	-4.1	-0.3	2000–2009	5.5*	2.0	10.9	-1.8*	-2.8	-1.0	2000–2008	2.8*	1.1	6.7
											2009–2012	-21.3*	-28.9	-4.2				2008–2013	-6.1*	-12.7	-2.6
											2012–2020	5.4*	0.9	29.9				2013–2021	1.8*	0.1	9.7
											2020–2023	-18.5*	-41.4	-2.8				2021–2023	-20.9*	-30.6	-9.5
Undetermined causes	0.3	-3.0	4.3	2000–2023	0.3	-3.0	4.3	-2.2	-5.6	1.1	2000–2011	4.1	-0.4	26.3	-1.9*	-3.4	-0.1	2000–2014	3.8*	1.7	6.5
											2011–2023	-7.6*	-23.1	-3.7				2014–2017	-32.9*	-41.3	-16.9
																		2017–2023	4.2	-4.0	29.8

Cause	45–64 years							65–79 years							80 years and older						
	AAPC	LCL^#^	UCL^#^	period	APC	LCL^#^	UCL^#^	AAPC	LCL^#^	UCL^#^	period	APC	LCL^#^	UCL^#^	AAPC	LCL^#^	UCL^#^	period	APC	LCL^#^	UCL^#^
All causes^i^	2.0*	1.7	2.3	2000–2014	0.9*	0.2	1.4	0.7*	0.4	1.0	2000–2017	-0.1	-0.8	0.3	2.1*	1.9	2.3	2000–2007	0.1	-1.9	1.0
				2014–2023	3.8*	3.0	5.0				2017–2023	3.1*	1.7	6.8				2007–2010	5.4*	2.8	7.0
																		2010–2016	0.5	-1.8	1.4
																		2016–2023	4.1*	3.4	5.1
All unintentional causes	3.0*	2.5	3.9	2000–2014	0.8	-0.7	3.5	0.9*	0.6	1.2	2000–2016	-0.3	-1.0	0.3	2.2*	2.0	2.4	2000–2007	0.1	-1.8	0.9
				2014–2021	8.9	-2.9	16.1				2016–2023	3.5*	2.2	6.9				2007–2010	5.6*	3.0	7.2
				2021–2023	-1.0	-8.2	7.3											2010–2016	0.5	-1.9	1.3
																		2016–2023	4.3*	3.7	5.3
*Falls*	1.7*	1.0	2.5	2000–2013	2.7*	1.9	8.5	3.4*	3.1	3.9	2000–2010	5.3*	4.3	7.2	5.7*	5.2	6.4	2000–2007	6.0	-2.6	9.0
				2013–2023	0.5	-4.9	1.5				2010–2015	-0.9	-4.9	1.5				2007–2010	15.4*	2.5	19.5
											2015–2023	3.8*	2.9	7.2				2010–2016	1.0	-3.5	3.6
																		2016–2023	5.6*	4.1	9.7
*Poisonings*	9.3*	8.3	10.7	2000–2013	6.4*	2.5	9.8	7.0*	6.0	8.2	2000–2014	3.9	-0.6	6.2	-0.5	-1.4	0.6	2000–2023	-0.5	-1.4	0.6
				2013–2021	16.6*	13.0	27.9				2014–2023	12.0*	9.7	18.3							
				2021–2023	-0.1	-9.1	12.3														
*Land transport incidents*	-2.1*	-2.5	-1.6	2000–2007	-0.3	-1.7	3.7	-3.5*	-4.0	-3.0	2000–2023	-3.5*	-4.0	-3.0	-3.0*	-3.8	-2.3	2000–2023	-3.0*	-3.8	-2.3
				2007–2011	-6.7*	-10.1	-3.4														
				2011–2023	-1.6	-2.5	1.1														
*Suffocation*	-0.5	-1.0	0.0	2000–2023	-0.5	-1.0	0.0	-0.3	-1.4	0.6	2000–2018	-1.7*	-6.4	-0.5	1.5*	0.9	2.4	2000–2012	1.0	-0.3	4.9
											2018–2023	5.0	-0.4	18.9				2012–2015	-11.6*	-15.9	-2.2
																		2015–2023	7.5*	5.2	12.0
* Drowning*	-0.8	-1.7	0.1	2000–2023	-0.8	-1.7	0.1	0.3	-0.6	1.4	2000–2023	0.3	-0.6	1.4	0.5	-0.7	2.0	2000–2023	0.5	-0.7	2.0
* Smoke/fire/flames*	-1.1*	-1.8	-0.3	2000–2023	-1.1*	-1.8	-0.3	-1.0	-2.0	0.0	2000–2023	-1.0	-2.0	0.0	-3.2*	-5.3	-1.0	2000–2023	-3.2*	-5.3	-1.0
* Struck by/against*	-2.7*	-4.2	-1.2	2000–2023	-2.7*	-4.2	-1.2	-1.1	-3.0	0.9	2000–2023	-1.1	-3.0	0.9	2.2*	0.3	4.8	2000–2023	2.2*	0.3	4.8
* Other unintentional causes*	0.3	-0.4	1.1	2000–2023	0.3	-0.4	1.1	-1.4*	-2.7	-0.2	2000–2013	-5.1*	-11.3	-2.8	-2.7*	-3.3	-1.9	2000–2003	0.5	-6.7	11.0
											2013–2023	3.6*	0.3	15.6				2003–2011	-8.7	-15.6	0.9
																		2011–2023	0.8	-0.7	2.8
Suicide	0.2	-0.4	1.0	2000–2015	0.7*	0.2	7.6	0.2	-0.1	0.6	2000–2023	0.2	-0.1	0.6	0.6	-0.2	1.5	2000–2015	1.8*	0.9	6.3
				2015–2023	-0.8	-6.6	0.3											2015–2023	-1.7	-8.7	0.2
Homicide	-2.0*	-3.7	-0.4	2000–2020	0.0	-0.8	2.6	-2.4*	-3.7	-1.1	2000–2023	-2.4*	-3.7	-1.1	-0.8	-2.9	1.6	2000–2023	-0.8	-2.9	1.6
				2020–2023	-14.3*	-34.1	-2.0														
Undetermined causes	-0.7	-1.8	0.7	2000–2004	16.1*	8.6	36.3	-1.5	-3.3	-0.2	2000–2013	-0.9	-5.3	6.7	-1.2	-2.8	0.5	2000–2023	-1.2	-2.8	0.5
				2004–2014	1.0	-1.1	2.6				2013–2018	-12.4	-26.6	5.1							
				2014–2017	-32.2*	-37.0	-22.3				2018–2023	8.8	-3.7	36.0							
				2017–2023	5.3*	0.5	15.0														

AAPC: average annual percent change APC: annual percent change

^#^ LCL: 95% lower confidence limit; UCL: 95% upper confidence limit

^i^ All causes exclude medical and surgical adverse effects, and legal intervention and operations of war

@ APC and AAPC were calculated based on combining years, [2000,2001], [2002,2003], …, [2022,2023]

& APC and AAPC were calculated based on combining years, [2000,2001], [2002,2003], …, [2022,2023]. The interval 2000–2012 was [2000,2001]–[2012,2013] and the interval 2012–2023 was [2012,2013]–[2022,2023]

^$^ Suicide counts for 0–9 year group are extremely small, so no joinpoint regression analysis was done

* *p*<0.05

The sensitivity analysis excluding 2023 data (additional file 2 and 3: Table A14 and A15) revealed that the overall trend patterns were similar. The direction and magnitude of the AAPCs, and the location of join points remained largely consistent with the primary analysis. The sensitivity analysis suggested that the inclusion of 2023 data did not substantially influence the overall trends.

## Discussion

Injuries, intentional and unintentional, remain a significant public health concern in Canada. This study examined the temporal trends in the leading causes of injury deaths in Canada from 2000 to 2023 with a focus on the change in the ranking of the leading causes and quantified trends. Falls, poisonings, suicide and land transport incidents were consistently the top four leading causes of injury deaths for both males and females during the study period, although their relative rankings and trends varied by sex. Age groups had different leading cause rankings and trends. For the total population, the ASMRs from poisonings and falls had an annual increase of 9.2% and 4.6% respectively. For land transport incidents, the ASMR steadily decreased annually by 3.1%. ASMR also decreased annually for struck by/against (2.1%), smoke/fire/flame (2.0%), homicide (1.7%), and drowning (0.8%). Compared to males, females showed a faster increase in the ASMR for falls and a more rapid decline for land transport incidents. Females had an annual increase of 0.8% in the suicide ASMR. Among individuals aged 0–44 years, crude mortality rates from falls decreased annually (1.1%–4.8%), whereas increases were observed in older age groups (1.7%–5.7%). Crude mortality rates from poisonings rose among those aged 10–79 years, with annual increases ranging from 6.5% to 9.3%.

### Falls

The deaths from falls have increased steadily over the last two decades as has been the leading cause of injury deaths since 2010 (except 2021). One of the main drivers is the aging population. Older people are at higher risk for falls due to a number of factors such as decreased mobility and balance, muscle weakness, visual impairment and adverse effects of medication, etc [23]. However, this study shows that the ASMR due to falls increased even after adjusting for the age structure. Improvement in reporting and changes regarding how to assign an underlying cause of death may have played a role in the increase of fall-caused deaths [24, 25]. The predominance of deaths from unspecified falls and other falls on the same level suggests that increases in fall mortality may be associated with everyday falls among older adults. The substantial contribution of unspecified falls indicates that improvements in the characterization and coding of fall circumstances are needed. The opposing trends observed for slip/trip-caused and unspecified falls from 2019 to 2023 suggest improved specificity in the reporting or coding of fall circumstances. U.S. studies have found that medication use and polypharmacy among adults aged 65 years and older have increased substantially over recent decades [26], and exposure to fall-risk-increasing medications is widespread [27]. Although national trend data on fall-risk-increasing medication use among older adults in Canada are limited, the Canadian Institute for Health Information reports that one-quarter of older adults were prescribed 10 or more drug classes in 2021 [28]. An Ontario study has shown that fall-risk-increasing medications were associated with increased risk of fall-related injuries among older adults [29]. Therefore, medication use may have contributed to the rise in fatal falls in Canada. Another consideration is, younger decedents are more likely to receive postmortem investigation, including autopsy and/or toxicology testing, while older adults who die often undergo less investigation [30]. In Canada, there is no single national policy governing investigation of fall-related deaths. Death investigations are conducted under provincial and territorial coroner or medical examiner legislation, and practices can vary across jurisdictions [31]. Limited postmortem investigation may contribute to misclassification of the underlying cause of death. For example, acute medical events such as stroke, cardiac arrhythmia, or drug overdose may precipitate falls but remain unrecognized, resulting in classification as fall-caused deaths. Conversely, injuries sustained in a fall may initiate a fatal sequence of events but ultimately be attributed to natural causes among older adults with multiple chronic conditions. If medication use contributes to a fatal fall, the absence of postmortem investigation may hinder identification of this important contributing factor. In terms of fall prevention, medication review and deprescribing of high-risk medications, where clinically appropriate, may be an important component of efforts to reduce fall-related morbidity and mortality [27]. Other effective fall prevention strategies include exercise, regular checkup for sight and hearing, hazard-free home and community environments and support rails and bars, etc [32].

### Poisonings

The deaths from poisonings had the largest increase during the study period, mainly from poisonings with psychoactive drugs and other/unspecified drugs, reflecting the increasing role of substances such as opioids and other psychoactive drugs in fatal poisonings. Canada’s illegal drug supply in recent years has become tainted with powerful opioids, such as fentanyl, and other drugs, such as benzodiazepines and xylazine. This toxic drug crisis has posed a major public health challenge in Canada [33]. The substantial contribution of other/unspecified drugs may also reflect the increasing complexity of fatal poisonings and potential limitations in the identification or classification of the substances involved. Canada has implemented a multifaceted approach to address the situation including public health initiatives, harm reduction strategies, law enforcement actions and international collaboration [34]. Challenges remain significant and debates around the balance between harm reduction approaches and law enforcement measures are ongoing [35]. Nevertheless, continued data collection and research are core elements of the approach.

### Suicide

Suicide rates remained relatively stable from 2000 to 2023, with no sustained decline. However, both the number of deaths and age-standardized mortality rates (ASMRs) increased from 2006 onward. Among females, ASMRs increased annually by 0.8% from 2000 to 2023, highlighting the need for targeted prevention in this group. National efforts aim to address these trends. The National Suicide Prevention Action Plan (2024–2027) provides a coordinated framework to reduce suicide rates [36]. Integration of coroner and medical examiner data with the Canadian Vital Statistics Death Database (CVSD), as used in this study, supports these efforts [16]. The 9-8-8 Suicide Crisis Helpline offers 24/7 nationwide support [37]. Continued surveillance is needed to assess the impact of these interventions.

### Land transport incidents

Deaths from land transport incidents declined steadily in both number and rate over the study period for all ages. The decline was primarily due to reductions in deaths from motor vehicle traffic crashes. This suggests that comprehensive approaches in Canada including regulatory measures, targeted initiatives, infrastructure improvements, and public awareness campaigns [38] have contributed to improved road safety and reduced injury mortality.

### Comparison with other countries

Compared with global estimates, Canada had higher mortality rates for both falls (global rate: 10.6 per 100 000 in 2023) and suicide (9.5 per 100 000 in 2023) [39]. In contrast, mortality from land transport incidents was substantially lower in Canada than the global rate (16.7 per 100 000 in 2023) [39]. Direct comparison for poisoning deaths was not possible due to differences in case definitions between global estimates and this study [40]. In terms of trends for leading causes of injury deaths, Canada showed patterns generally similar to some other high-income countries over the past two decades. Mortality from land transport incidents declined in the UK and Australia, while fall-related mortality increased in the UK, Australia, and the United States [39]. Drug poisoning deaths rose sharply in the United States [41], whereas suicide rates remained relatively stable in the UK and Australia [39].

#### Strengths and limitations

A strength of this study is the inclusion of more timely suicide data to partially address reporting delays in CVSD; suicide counts in CVSD were 12.9%–19.0% lower for 2021–2023 (Additional file 4: Table A16). However, for suicide and other injury deaths such as poisonings and homicide, some cases may require a lengthy investigation, which would still result in underreporting for the more recent years of data. Deaths with undetermined or pending causes are coded in CVSD as “R99,” including “R99.1” and “R99.2,” and are updated once investigations are completed [8]. Elevated counts of these codes in 2023 (10,564 vs. 3,350–7,954 in 2020–2022) [42] suggest that injury-related mortality will likely be revised upward, especially for 2023. However, we do not expect changes in ranking. In addition, suicide mortality may be differentially misclassified by sex because methods that are more common among females, particularly poisonings, can be more difficult to distinguish from unintentional or undetermined deaths than methods more common among males, such as hanging or firearms. Consequently, female suicide mortality may be somewhat underestimated. However, available Canadian evidence suggests that the higher suicide mortality observed among males persists even after accounting for potential misclassification [43]. Furthermore, the suicide data from the P/T chief coroner and chief medical examiner offices were only available in a broad age grouping due to confidentiality, so the stratification for the suicide ASMRs was based on a wider interval than the 5-year interval for other causes. Even so, the rankings were not affected since they were based on the crude mortality rates. Our assessment shows that the wider interval used in this study had little impact on the ASMRs (Additional file 4: Table A17). Moreover, this study aimed to collectively compare the leading causes of injury deaths in terms of a relative broad categorization, so we did not stratify the sub-cause analysis by sex and age, which can help understand the demographics of main drivers in the changes. Nevertheless, this study has set a stage for a follow-up study that will focus on the sub-causes. Finally, this study examined national trends only and did not assess variation by geography or socioeconomic status. Future research should examine disparities among specific populations, including First Nations, Inuit, and Métis communities, as well as rural, remote, and socioeconomic groups. Health data disparities should also be considered, such as disparities in the quality or completeness of health data, since unequal data quality across population groups could lead to differential misclassification of injury deaths.

## Conclusion

The findings from this study provide insights on key shifts of leading causes of injury deaths in Canada. They highlight the need for sustained, cause-specific prevention effort. Timely and complete surveillance remains essential to accurately monitor trends, and evaluate effectiveness of interventions and guide targeted, equitable prevention strategies.

## Supplementary Information

Supplementary Material 1: Number of deaths and crude and age-standardized mortality rates of injury deaths by cause, age, sex in Canada from 2020 to 2023

Supplementary Material 2: Sensitivity analysis for the joinpoint analysis of age-standardized mortality rates of injury deaths by cause, total population and sex

Supplementary Material 3: Sensitivity analysis for the joinpoint analysis of crude mortality rates of injury deaths by cause by age group

Supplementary Material 4: 1. Comparison of suicide numbers from 2020 to 2023 between this study and the Canadian Vital Statistics - Death database; 2. Canadian age-standardized suicide rates (per 100 000 population) with a stratification based on a wider age interval (0–9, 10–14, 15–19, 20–24, 25–44, 45–64, 65–79, 80+) compared to a 5-year interval from 2000 to 2019

## Data Availability

The CVSD data used in this study are available at: [https://www150.statcan.gc.ca/t1/tbl1/en/cv.action? pid=1310015601](https:/www150.statcan.gc.ca/t1/tbl1/en/cv.action? pid=1310015601) . The suicide 2020-2023 data are available at: [https://health-infobase.canada.ca/mental-health/suicide-self-harm/suicide-mortality.html](https:/health-infobase.canada.ca/mental-health/suicide-self-harm/suicide-mortality.html) .

## Abbreviations

AAPC: Average annual percent change
APC: Annual percent change
ASMR: Age-standardized mortality rate
CCMED: Canadian Coroner and Medical Examiner Database
CI: Confidence interval
CVSD: Canadian Vital Statistics - Death database
ICD-10: International Statistical Classification of Diseases and Related Health Problems 10th Revision
MVT: Motor vehicle traffic crashes
P/T: Provincial/territorial
RECORD: Reporting of Studies Conducted using Observational Routinely-Collected Health Data
