# Supplementary material for: Temporal trends in leading causes of injury deaths in Canada from 2000 to 2023: a time series analysis of mortality data
Source: Inj Epidemiol. 2026 Aug 6;13:61. doi: 10.1186/s40621-026-00702-4 (PMC13587287; doi:10.1186/s40621-026-00702-4)
Supplement: Supplementary file 1 [file 40621_2026_702_MOESM1_ESM.docx]

**Table A1-1 to A13-2**

Notes:

- In the following tables, for simplicity, the qualifier “unintentional” is omitted for falls, poisonings, land transport incidents, suffocation, drowning, smoke/fire/flames and struck by/against.
- For total population (Table A1-1 to A1-4), the deaths with unknown sex and/or unknown age were included, and the deaths with unknown age were exclude for age-standardized mortality rates (Table A1-5, A1-6). The sex- or age-specific numbers and rates (Table A2s – A9s) excluded the deaths with unknown sex or unknown age.
- The suicide numbers from 2020 to 2023 for those aged 0 to 9 years are suppressed due to small count. For the suicide from 2000 to 2019 and other causes from 2000 to 2023, small numbers are not suppressed since they are publicly available from Statistics Canada’s website ([Add/Remove data - Deaths, by cause, Chapter XX: External causes of morbidity and mortality (V01 to Y89)](https://www150.statcan.gc.ca/t1/tbl1/en/cv.action?pid=1310015601)).

Table A1-1: Number of deaths due to leading causes, total population, Canada, from 2000 to 2023

| Year | Falls | Poisonings | Land transport incidents | Suffocation | Drowning | Smoke/fire/flames | Struck by/against | Suicide | Homicide |
| --- | --- | --- | --- | --- | --- | --- | --- | --- | --- |
| 2000 | 1 570 | 963 | 2 999 | 438 | 349 | 242 | 112 | 3 643 | 484 |
| 2001 | 1 734 | 973 | 2 893 | 392 | 361 | 250 | 120 | 3 742 | 496 |
| 2002 | 1 779 | 1 030 | 3 060 | 447 | 377 | 224 | 87 | 3 725 | 490 |
| 2003 | 1 989 | 893 | 2 954 | 424 | 356 | 198 | 78 | 3 787 | 468 |
| 2004 | 2 225 | 957 | 2 901 | 401 | 347 | 236 | 91 | 3 629 | 558 |
| 2005 | 2 306 | 1 055 | 3 133 | 399 | 342 | 215 | 106 | 3 762 | 623 |
| 2006 | 2 611 | 1 155 | 3 068 | 426 | 362 | 200 | 95 | 3 568 | 556 |
| 2007 | 2 740 | 1 392 | 3 017 | 473 | 340 | 216 | 104 | 3 654 | 566 |
| 2008 | 3 119 | 1 437 | 2 712 | 441 | 358 | 275 | 106 | 3 718 | 589 |
| 2009 | 3 641 | 1 509 | 2 476 | 429 | 318 | 225 | 82 | 3 931 | 587 |
| 2010 | 4 067 | 1 577 | 2 549 | 446 | 372 | 226 | 90 | 3 975 | 537 |
| 2011 | 4 276 | 1 701 | 2 335 | 518 | 346 | 195 | 86 | 3 938 | 565 |
| 2012 | 4 510 | 1 796 | 2 415 | 492 | 376 | 191 | 94 | 3 997 | 506 |
| 2013 | 4 709 | 1 841 | 2 268 | 448 | 356 | 242 | 100 | 4 057 | 462 |
| 2014 | 4 898 | 2 090 | 2 122 | 433 | 328 | 228 | 94 | 4 292 | 461 |
| 2015 | 4 777 | 2 469 | 2 203 | 437 | 344 | 213 | 93 | 4 486 | 510 |
| 2016 | 4 946 | 3 569 | 2 251 | 439 | 330 | 215 | 87 | 4 450 | 475 |
| 2017 | 5 232 | 4 865 | 2 111 | 486 | 393 | 191 | 87 | 4 478 | 564 |
| 2018 | 5 765 | 4 964 | 2 191 | 487 | 363 | 204 | 107 | 4 595 | 522 |
| 2019 | 6 340 | 4 490 | 2 007 | 523 | 330 | 206 | 88 | 4 621 | 546 |
| 2020 | 6 832 | 6 680 | 1 910 | 523 | 442 | 190 | 100 | 4 523 | 582 |
| 2021 | 7 199 | 7 690 | 1 882 | 563 | 386 | 240 | 91 | 4 508 | 497 |
| 2022 | 8 120 | 7 472 | 2 095 | 651 | 376 | 253 | 79 | 4 982 | 499 |
| 2023 | 8 045 | 7 475 | 1 861 | 650 | 355 | 191 | 82 | 4 735 | 354 |

Table A1-2: Number of deaths from all causes, all unintentional causes, other unintentional causes and undetermined causes, total population, Canada, from 2000 to 2023

| Year | All causes | All unintentional causes | Other unintentional causes | Undetermined causes |
| --- | --- | --- | --- | --- |
| 2000 | 13 163 | 8 631 | 1 958 | 405 |
| 2001 | 13 257 | 8 594 | 1 871 | 425 |
| 2002 | 13 809 | 9 149 | 2 145 | 445 |
| 2003 | 14 013 | 9 108 | 2 216 | 650 |
| 2004 | 13 838 | 9 010 | 1 852 | 641 |
| 2005 | 14 677 | 9 592 | 2 036 | 700 |
| 2006 | 14 572 | 9 817 | 1 900 | 631 |
| 2007 | 14 962 | 1 0073 | 1 791 | 669 |
| 2008 | 15 235 | 10 238 | 1 790 | 690 |
| 2009 | 15 444 | 10 238 | 1 558 | 688 |
| 2010 | 16 169 | 10 915 | 1 588 | 742 |
| 2011 | 16 219 | 11 011 | 1 554 | 705 |
| 2012 | 16 646 | 11 354 | 1 480 | 789 |
| 2013 | 16 837 | 11 523 | 1 559 | 795 |
| 2014 | 17 371 | 11 789 | 1 596 | 829 |
| 2015 | 17 824 | 12 130 | 1 594 | 698 |
| 2016 | 18 804 | 13 577 | 1 740 | 302 |
| 2017 | 20 473 | 15 103 | 1 738 | 328 |
| 2018 | 21 408 | 16 028 | 1 947 | 263 |
| 2019 | 21 408 | 15 910 | 1 926 | 331 |
| 2020 | 24 036 | 18 529 | 1 852 | 402 |
| 2021 | 25 897 | 20 481 | 2 430 | 411 |
| 2022 | 26 965 | 21 097 | 2 051 | 387 |
| 2023 | 26 180 | 20 709 | 2 050 | 382 |

Table A1-3: Crude mortality rates (per 100 000 population) due to leading causes, total population, Canada, from 2000 to 2023 (with 95% confidence intervals)

| Year | Falls | Poisonings | Land transport incidents | Suffocation | Drowning | Smoke/fire/ flames | Struck by/against | Suicide | Homicide |
| --- | --- | --- | --- | --- | --- | --- | --- | --- | --- |
| 2000 | 5.1 [4.9, 5.4] | 3.1 [2.9, 3.3] | 9.8 [9.4, 10.1] | 1.4 [1.3, 1.6] | 1.1 [1.0, 1.3] | 0.8 [0.7, 0.9] | 0.4 [0.3, 0.4] | 11.9 [11.5, 12.3] | 1.6 [1.4, 1.7] |
| 2001 | 5.6 [5.3, 5.9] | 3.1 [2.9, 3.3] | 9.3 [9.0, 9.7] | 1.3 [1.1, 1.4] | 1.2 [1.0, 1.3] | 0.8 [0.7, 0.9] | 0.4 [0.3, 0.5] | 12.1 [11.7, 12.4] | 1.6 [1.5, 1.7] |
| 2002 | 5.7 [5.4, 5.9] | 3.3 [3.1, 3.5] | 9.8 [9.4, 10.1] | 1.4 [1.3, 1.6] | 1.2 [1.1, 1.3] | 0.7 [0.6, 0.8] | 0.3, [0.2, 0.3] | 11.9 [11.5, 12.3] | 1.6 [1.4, 1.7] |
| 2003 | 6.3 [6.0, 6.6] | 2.8 [2.6, 3.0] | 9.3 [9.0, 9.7] | 1.3 [1.2, 1.5] | 1.1 [1.0, 1.2] | 0.6 [0.5, 0.7] | 0.2 [0.2, 0.3] | 12.0 [11.6, 12.3] | 1.5 [1.3, 1.6] |
| 2004 | 7.0 [6.7, 7.3] | 3.0 [2.8, 3.2] | 9.1 [8.8, 9.4] | 1.3 [1.1, 1.4] | 1.1 [1.0, 1.2] | 0.7 [0.6, 0.8] | 0.3 [0.2, 0.3] | 11.4 [11.0, 11.7] | 1.7 [1.6, 1.9] |
| 2005 | 7.2 [6.9, 7.4] | 3.3 [3.1, 3.5] | 9.7 [9.4, 10.1] | 1.2 [1.1, 1.4] | 1.1 [0.9, 1.2] | 0.7 [0.6, 0.8] | 0.3 [0.3, 0.4] | 11.7 [11.3, 12.0] | 1.9 [1.8, 2.1] |
| 2006 | 8.0 [7.7, 8.3] | 3.5 [3.3, 3.8] | 9.4 [9.1, 9.8] | 1.3 [1.2, 1.4] | 1.1 [1.0, 1.2] | 0.6 [0.5, 0.7] | 0.3 [0.2, 0.4] | 11.0 [10.6, 11.3] | 1.7 [1.6, 1.8] |
| 2007 | 8.3 [8.0, 8.6] | 4.2 [4.0, 4.5] | 9.2 [8.8, 9.5] | 1.4 [1.3, 1.6] | 1.0 [0.9, 1.1] | 0.7 [0.6, 0.7] | 0.3 [0.3, 0.4] | 11.1 [10.7, 11.5] | 1.7 [1.6, 1.9] |
| 2008 | 9.4 [9.1, 9.7] | 4.3 [4.1, 4.5] | 8.2 [7.9, 8.5] | 1.3 [1.2, 1.5] | 1.1 [1.0, 1.2] | 0.8 [0.7, 0.9] | 0.3 [0.3, 0.4] | 11.2 [10.8, 11.5] | 1.8 [1.6, 1.9] |
| 2009 | 10.8 [10.5, 11.2] | 4.5 [4.3, 4.7] | 7.4 [7.1, 7.7] | 1.3 [1.2, 1.4] | 0.9 [0.8, 1.0] | 0.7 [0.6, 0.8] | 0.2 [0.2, 0.3] | 11.7 [11.3, 12.1] | 1.7 [1.6, 1.9] |
| 2010 | 12.0 [11.6, 12.3] | 4.6 [4.4, 4.9] | 7.5 [7.2, 7.8] | 1.3 [1.2, 1.4] | 1.1 [1.0, 1.2] | 0.7 [0.6, 0.8] | 0.3 [0.2, 0.3] | 11.7 [11.3, 12.1] | 1.6 [1.4, 1.7] |
| 2011 | 12.5 [12.1, 12.8] | 5.0 [4.7, 5.2] | 6.8 [6.5, 7.1] | 1.5 [1.4, 1.6] | 1.0 [0.9, 1.1] | 0.6 [0.5, 0.6] | 0.3 [0.2, 0.3] | 11.5 [11.1, 11.8] | 1.6 [1.5, 1.8] |
| 2012 | 13.0 [12.6, 13.4] | 5.2 [4.9, 5.4] | 7.0 [6.7, 7.2] | 1.4 [1.3, 1.5] | 1.1 [1.0, 1.2] | 0.5 [0.5, 0.6] | 0.3 [0.2, 0.3] | 11.5 [11.2, 11.9] | 1.5 [1.3, 1.6] |
| 2013 | 13.4 [13.0, 13.8] | 5.2 [5.0, 5.5] | 6.5 [6.2, 6.7] | 1.3 [1.2, 1.4] | 1.0 [0.9, 1.1] | 0.7 [0.6, 0.8] | 0.3 [0.2, 0.3] | 11.6 [11.2, 11.9] | 1.3 [1.2, 1.4] |
| 2014 | 13.8 [13.4, 14.2] | 5.9 [5.6, 6.2] | 6.0 [5.7, 6.2] | 1.2 [1.1, 1.3] | 0.9 [0.8, 1.0] | 0.6 [0.6, 0.7] | 0.3 [0.2, 0.3] | 12.1 [11.8, 12.5] | 1.3 [1.2, 1.4] |
| 2015 | 13.4 [13.0, 13.8] | 6.9 [6.6, 7.2] | 6.2 [5.9, 6.4] | 1.2 [1.1, 1.3] | 1.0 [0.9, 1.1] | 0.6 [0.5, 0.7] | 0.3 [0.2, 0.3] | 12.6 [12.2, 12.9] | 1.4 [1.3, 1.6] |
| 2016 | 13.7 [13.3, 14.1] | 9.9 [9.6, 10.2] | 6.2 [6.0, 6.5] | 1.2 [11, 1.3] | 0.9 [0.8, 1.0] | 0.6 [0.5, 0.7] | 0.2 [0.2, 0.3] | 12.3 [12.0, 12.7] | 1.3 [1.2, 1.4] |
| 2017 | 14.3 [13.9, 14.7] | 13.3 [12.9, 13.7] | 5.8 [5.5, 6.0] | 1.3 [1.2, 1.4] | 1.1 [1.0, 1.2] | 0.5 [0.4, 0.6] | 0.2 [0.2, 0.3] | 12.3 [11.9, 12.6] | 1.5 [1.4, 1.7] |
| 2018 | 15.6 [15.1, 16.0] | 13.4 [13.0, 13.8] | 5.9 [5.7, 6.2] | 1.3 [1.2, 1.4] | 1.0 [0.9, 1.1] | 0.6 [0.5, 0.6] | 0.3 [0.2, 0.3] | 12.4 [12.0, 12.8] | 1.4 [1.3, 1.5] |
| 2019 | 16.9 [16.4, 17.3] | 11.9 [11.6, 12.3] | 5.3 [5.1, 5.6] | 1.4 [1.3, 1.5] | 0.9 [0.8, 1.0] | 0.5 [0.5, 0.6] | 0.2 [0.2, 0.3] | 12.3 [11.9, 12.6] | 1.5 [1.3, 1.6] |
| 2020 | 18.0 [17.5, 18.4] | 17.6 [17.1, 18.0] | 5.0 [4.8, 5.2] | 1.4 [1.3, 1.5] | 1.2 [1.1, 1.3] | 0.5 [0.4, 0.6] | 0.3 [0.2, 0.3] | 11.9 [11.5, 12.2] | 1.5 [1.4, 1.7] |
| 2021 | 18.8 [18.4, 19.3] | 20.1 [19.7, 20.6] | 4.9 [4.7, 5.1] | 1.5 [1.4, 1.6] | 1.0 [0.9, 1.1] | 0.6 [0.5, 0.7] | 0.2 [0.2, 0.3] | 11.8 [11.4, 12.1] | 1.3 [1.2, 1.4] |
| 2022 | 20.8 [20.4, 21.3] | 19.2 [18.7, 19.6] | 5.4 [5.1, 5.6] | 1.7 [1.5, 1.8] | 1.0 [0.9, 1.1] | 0.6 [0.6, 0.7] | 0.2 [0.2, 0.2] | 12.8 [12.4, 13.1] | 1.3 [1.2, 1.4] |
| 2023 | 20.1 [19.6, 20.5] | 18.7 [18.2, 19.1] | 4.6 [4.4, 4.9] | 1.6 [1.5, 1.7] | 0.9 [0.8, 1.0] | 0.5 [0.4, 0.5] | 0.2 [0.2, 0.2] | 11.8 [11.5, 12.2] | 0.9 [0.8, 1.0] |

Table A1-4: Crude mortality rates (per 100 000 population) from all causes, all unintentional causes, other unintentional causes and undetermined causes, total population, Canada, from 2000 to 2023 (with 95% confidence intervals)

| Year | All causes | All unintentional causes | Other unintentional causes | Undetermined causes |
| --- | --- | --- | --- | --- |
| 2000 | 42.9 [42.2, 43.6] | 28.1 [27.5, 28.7] | 6.4 [6.1, 6.7] | 1.3 [1.2, 1.4] |
| 2001 | 42.7 [42.0, 43.5] | 27.7 [27.1, 28.3] | 6.0 [5.8, 6.3] | 1.4 [1.2, 1.5] |
| 2002 | 44.0 [43.3, 44.8] | 29.2 [28.6, 29.8] | 6.8 [6.6, 7.1] | 1.4 [1.3, 1.6] |
| 2003 | 44.3 [43.6, 45.0] | 28.8 [28.2, 29.4] | 7.0 [6.7, 7.3] | 2.1 [1.9, 2.2] |
| 2004 | 43.3 [42.6, 44.0] | 28.2 [27.6, 28.8] | 5.8 [5.5, 6.1] | 2.0 [1.9, 2.2] |
| 2005 | 45.5 [44.8, 46.3] | 29.7 [29.2, 30.3] | 6.3 [6.0, 6.6] | 2.2 [2.0, 2.3] |
| 2006 | 44.7 [44.0, 45.5] | 30.1 [29.5, 30.7] | 5.8 [5.6, 6.1] | 1.9 [1.8, 2.1] |
| 2007 | 45.5 [44.8, 46.2] | 30.6 [30.0, 31.2] | 5.4 [5.2, 5.7] | 2.0 [1.9, 2.2] |
| 2008 | 45.8 [45.1, 46.6] | 30.8 [30.2, 31.4] | 5.4 [5.1, 5.6] | 2.1 [1.9, 2.2] |
| 2009 | 45.9 [45.2, 46.6] | 30.4 [29.9, 31.0] | 4.6 [4.4, 4.9] | 2.0 [1.9, 2.2] |
| 2010 | 47.5 [46.8, 48.3] | 32.1 [31.5, 32.7] | 4.7 [4.4, 4.9] | 2.2 [2.0, 2.3] |
| 2011 | 47.2 [46.5, 48.0] | 32.1 [31.5, 32.7] | 4.5 [4.3, 4.8] | 2.1 [1.9, 2.2] |
| 2012 | 48.0 [47.2, 48.7] | 32.7 [32.1, 33.3] | 4.3 [4.0, 4.5] | 2.3 [2.1, 2.4] |
| 2013 | 48.0 [47.3, 48.7] | 32.8 [32.2, 33.4] | 4.4 [4.2, 4.7] | 2.3 [2.1, 2.4] |
| 2014 | 49.0 [48.3, 49.8] | 33.3 [32.7, 33.9] | 4.5 [4.3, 4.7] | 2.3 [2.2, 2.5] |
| 2015 | 49.9 [49.2, 50.7] | 34.0 [33.4, 34.6] | 4.5 [4.2, 4.7] | 2.0 [1.8, 2.1] |
| 2016 | 52.1 [51.3, 52.8] | 37.6 [37.0, 38.2] | 4.8 [4.6, 5.0] | 0.8 [0.7, 0.9] |
| 2017 | 56.0 [55.3, 56.8] | 41.3 [40.7, 42.0] | 4.8 [4.5, 5.0] | 0.9 [0.8, 1.0] |
| 2018 | 57.7 [57.0, 58.5] | 43.2 [42.6, 43.9] | 5.3 [5.0, 5.5] | 0.7 [0.6, 0.8] |
| 2019 | 56.9 [56.1, 57.7] | 42.3 [41.6, 43.0] | 5.1 [4.9, 5.3] | 0.9 [0.8, 1.0] |
| 2020 | 63.2 [62.4, 64.0] | 48.7 [48.0, 49.4] | 4.9 [4.6, 5.1] | 1.1 [1.0, 1.2] |
| 2021 | 67.7 [66.9, 68.5] | 53.6 [52.8, 54.3] | 6.4 [6.1, 6.6] | 1.1 [1.0, 1.2] |
| 2022 | 69.2 [68.4, 70.1] | 54.2 [53.4, 54.9] | 5.3 [5.0, 5.5] | 1.0 [0.9, 1.1] |
| 2023 | 65.4 [64.6, 66.2] | 51.7 [51.0, 52.4] | 5.1 [4.9, 5.3] | 1.0 [0.9, 1.0] |

Table A1-5: Age-standardized mortality rates (per 100 000 population) due to leading causes, total population, Canada, from 2000 to 2023 (with 95% confidence intervals)

| Year | Falls | Poisonings | Land transport incidents | Suffocation | Drowning | Smoke/file/ flames | Struck by/against | Suicide | Homicide |
| --- | --- | --- | --- | --- | --- | --- | --- | --- | --- |
| 2000 | 7.7 [7.3, 8.1] | 3.0 [2.8, 3.3] | 10.1 [9.8, 10.5] | 1.8 [1.6, 2.0] | 1.1 [1.0, 1.3] | 0.9 [0.8, 1.0] | 0.4 [0.3, 0.5] | 12.1 [11.7, 12.5] | 1.5 [1.4, 1.7] |
| 2001 | 8.3 [7.9, 8.7] | 3.0 [2.8, 3.2] | 9.6 [9.2, 10.0] | 1.6 [1.4, 1.8] | 1.2 [1.0, 1.3] | 0.9 [0.8, 1.0] | 0.4 [0.3, 0.5] | 12.3 [11.9, 12.7] | 1.5 [1.4, 1.6] |
| 2002 | 8.2 [7.8, 8.6] | 3.2 [3.0, 3.4] | 10.1 [9.7, 10.4] | 1.8 [1.6, 2.0] | 1.2 [1.1, 1.3] | 0.8 [0.7, 0.9] | 0.3 [0.2, 0.4] | 12.1 [11.7, 12.5] | 1.5 [1.4, 1.7] |
| 2003 | 8.9 [8.5, 9.3] | 2.8 [2.6, 2.9] | 9.5 [9.1, 9.8] | 1.7 [1.5, 1.9] | 1.1 [1.0, 1.3] | 0.7 [0.6, 0.8] | 0.3 [0.2, 0.3] | 12.2 [11.8, 12.6] | 1.4 [1.3, 1.6] |
| 2004 | 9.7 [9.3, 10.2] | 2.9 [2.7, 3.1] | 9.3 [9.0, 9.7] | 1.5 [1.4, 1.7] | 1.1 [1.0, 1.2] | 0.8 [0.7, 0.9] | 0.3 [0.2, 0.4] | 11.5 [11.1, 11.9] | 1.7 [1.6, 1.9] |
| 2005 | 9.7 [9.3, 10.1] | 3.1 [2.9, 3.3] | 9.9 [9.5, 10.3] | 1.5 [1.4, 1.7] | 1.1 [0.9, 1.2] | 0.7 [0.6, 0.8] | 0.3 [0.3, 0.4] | 11.8 [11.4, 12.2] | 1.9 [1.7, 2.1] |
| 2006 | 10.7 [10.3, 11.1] | 3.4 [3.2, 3.6] | 9.5 [9.1, 9.8] | 1.6 [1.4, 1.7] | 1.1 [1.0, 1.3] | 0.6 [0.6, 0.7] | 0.3 [0.3, 0.4] | 11.1 [10.8, 11.5] | 1.6 [1.5, 1.8] |
| 2007 | 10.9 [10.4, 11.3] | 4.1 [3.9, 4.4] | 9.2 [8.9, 9.6] | 1.7 [1.6, 1.9] | 1.1 [1.0, 1.2] | 0.7 [0.6, 0.8] | 0.4 [0.3, 0.4] | 11.1 [10.8, 11.5] | 1.7 [1.5, 1.8] |
| 2008 | 12.0 [11.6, 12.5] | 4.2 [4.0, 4.4] | 8.2 [7.9, 8.6] | 1.6 [1.4, 1.7] | 1.1 [1.0, 1.2] | 0.9 [0.8, 1.0] | 0.3 [0.3, 0.4] | 11.2 [10.9, 11.6] | 1.7 [1.6, 1.8] |
| 2009 | 13.6 [13.2, 14.1] | 4.3 [4.1, 4.6] | 7.4 [7.1, 7.7] | 1.5 [1.4, 1.6] | 1.0 [0.8, 1.1] | 0.7 [0.6, 0.8] | 0.3 [0.2, 0.3] | 11.7 [11.3, 12.0] | 1.7 [1.6, 1.8] |
| 2010 | 14.7 [14.3, 15.2] | 4.5 [4.2, 4.7] | 7.6 [7.3, 7.9] | 1.5 [1.4, 1.7] | 1.1 [1.0, 1.2] | 0.7 [0.6, 0.8] | 0.3 [0.2, 0.4] | 11.6 [11.3, 12.0] | 1.5 [1.4, 1.7] |
| 2011 | 14.9 [14.5, 15.4] | 4.8 [4.6, 5.0] | 6.9 [6.6, 7.2] | 1.7 [1.6, 1.9] | 1.0 [0.9, 1.1] | 0.6 [0.5, 0.7] | 0.3 [0.2, 0.3] | 11.4 [11.1, 11.8] | 1.6 [1.5, 1.7] |
| 2012 | 15.2 [14.8, 15.7] | 5.0 [4.8, 5.2] | 7.0 [6.7, 7.3] | 1.6 [1.4, 1.7] | 1.1 [1.0, 1.2] | 0.6 [0.5, 0.7] | 0.3 [0.2, 0.3] | 11.4 [11.1, 11.8] | 1.4 [1.3, 1.6] |
| 2013 | 15.4 [14.9, 15.8] | 5.1 [4.9, 5.4] | 6.5 [6.3, 6.8] | 1.4 [1.3, 1.5] | 1.0 [0.9, 1.1] | 0.7 [0.6, 0.8] | 0.3 [0.2, 0.4] | 11.5 [11.2, 11.9] | 1.3 [1.2, 1.4] |
| 2014 | 15.5 [15.1, 15.9] | 5.8 [5.5, 6.0] | 6.1 [5.8, 6.3] | 1.3 [1.2, 1.4] | 0.9 [0.8, 1.0] | 0.7 [0.6, 0.8] | 0.3 [0.2, 0.3] | 12.1 [11.7, 12.4] | 1.3 [1.2, 1.4] |
| 2015 | 14.8 [14.3, 15.2] | 6.8 [6.5, 7.0] | 6.2 [6.0, 6.5] | 1.3 [1.2, 1.4] | 1.0 [0.9, 1.1] | 0.6 [0.5, 0.7] | 0.3 [0.2, 0.3] | 12.5 [12.1, 12.9] | 1.4 [1.3, 1.5] |
| 2016 | 14.8 [14.4, 15.2] | 9.7 [9.4, 10.0] | 6.3 [6.0, 6.6] | 1.3 [1.2, 1.4] | 0.9 [0.8, 1.0] | 0.6 [0.5, 0.7] | 0.2 [0.2, 0.3] | 12.3 [11.9, 12.7] | 1.3 [1.2, 1.4] |
| 2017 | 15.2 [14.8, 15.7] | 13.2 [12.8, 13.5] | 5.8 [5.6, 6.1] | 1.4 [1.3, 1.5] | 1.1 [1.0, 1.2] | 0.5 [0.5, 0.6] | 0.2 [0.2, 0.3] | 12.2 [11.9, 12.6] | 1.5 [1.4, 1.7] |
| 2018 | 16.3 [15.9, 16.8] | 13.3 [12.9, 13.7] | 5.9 [5.7, 6.2] | 1.4 [1.2, 1.5] | 1.0 [0.9, 1.1] | 0.6 [0.5, 0.6] | 0.3 [0.2, 0.4] | 12.4 [12.0, 12.7] | 1.4 [1.3, 1.5] |
| 2019 | 17.5 [17.1, 17.9] | 11.9 [11.5, 12.2] | 5.4 [5.1, 5.6] | 1.4 [1.3, 1.6] | 0.9 [0.8, 1.0] | 0.6 [0.5, 0.6] | 0.2 [0.2, 0.3] | 12.3 [11.9, 12.6] | 1.4 [1.3, 1.6] |
| 2020 | 18.4 [18.0, 18.9] | 17.5 [17.1, 17.9] | 5.0 [4.8, 5.3] | 1.4 [1.3, 1.5] | 1.2 [1.1, 1.3] | 0.5 [0.4, 0.6] | 0.3 [0.2, 0.3] | 11.9 [11.6, 12.2] | 1.5 [1.4, 1.7] |
| 2021 | 18.8 [18.4, 19.3] | 20.1 [19.7, 20.6] | 4.9 [4.7, 5.1] | 1.5 [1.4, 1.6] | 1.0 [0.9, 1.1] | 0.6 [0.6, 0.7] | 0.2 [0.2, 0.3] | 11.8 [11.4, 12.1] | 1.3 [1.2, 1.4] |
| 2022 | 20.6 [20.2, 21.1] | 19.2 [18.8, 19.7] | 5.4 [5.1, 5.6] | 1.7 [1.5, 1.8] | 1.0 [0.9, 1.1] | 0.6 [0.6, 0.7] | 0.2 [0.2, 0.3] | 12.8 [12.4, 13.1] | 1.3 [1.2, 1.4] |
| 2023 | 19.9 [19.4, 20.3] | 18.7 [18.3, 19.1] | 4.6 [4.4, 4.8] | 1.6 [1.5, 1.7] | 0.9 [0.8, 1.0] | 0.5 [0.4, 0.5] | 0.2 [0.2, 0.3] | 11.8 [11.5, 12.2] | 0.9 [0.8, 1.0] |

Table A1-6: Age-standardized mortality rates (per 100 000 population) from all causes, all unintentional causes, other unintentional causes and undetermined causes, total population, Canada, from 2000 to 2023 (with 95% confidence intervals)

| Year | All causes | All unintentional causes | Other unintentional causes | Undetermined causes |
| --- | --- | --- | --- | --- |
| 2000 | 48.6 [47.7, 49.4] | 34.8 [34.0, 35.6] | 9.6 [9.2, 10.1] | 1.4 [1.2, 1.5] |
| 2001 | 47.8 [47.0, 48.7] | 33.9 [33.1, 34.6] | 8.8 [8.4, 9.3] | 1.4 [1.2, 1.5] |
| 2002 | 49.2 [48.3, 50.0] | 35.4 [34.6, 36.1] | 9.9 [9.5, 10.4] | 1.4 [1.3, 1.6] |
| 2003 | 49.2 [48.3, 50.0] | 34.9 [34.1, 35.6] | 10.0 [9.6, 10.4] | 2.0 [1.9, 2.2] |
| 2004 | 47.7 [46.9, 48.5] | 33.8 [33.1, 34.5] | 8.1 [7.7, 8.4] | 2.0 [1.8, 2.1] |
| 2005 | 49.5 [48.7, 50.3] | 34.9 [34.2, 35.6] | 8.5 [8.1, 8.9] | 2.1 [1.9, 2.3] |
| 2006 | 48.4 [47.6, 49.2] | 34.9 [34.2, 35.6] | 7.6 [7.3, 8.0] | 1.9 [1.7, 2.0] |
| 2007 | 48.8 [48.0, 49.6] | 35.0 [34.3, 35.7] | 6.9 [6.6, 7.3] | 1.9 [1.8, 2.1] |
| 2008 | 49.0 [48.2, 49.7] | 35.1 [34.4, 35.8] | 6.7 [6.4, 7.0] | 2.0 [1.9, 2.2] |
| 2009 | 48.8 [48.0, 49.6] | 34.4 [33.8, 35.1] | 5.6 [5.3, 5.9] | 2.0 [1.8, 2.1] |
| 2010 | 50.3 [49.5, 51.1] | 35.9 [35.2, 36.6] | 5.5 [5.3, 5.8] | 2.1 [2.0, 2.3] |
| 2011 | 49.7 [48.9, 50.5] | 35.4 [34.8, 36.1] | 5.2 [5.0, 5.5] | 2.0 [1.8, 2.1] |
| 2012 | 49.9 [49.2, 50.7] | 35.5 [34.9, 36.2] | 4.8 [4.6, 5.1] | 2.2 [2.1, 2.4] |
| 2013 | 49.8 [49.1, 50.6] | 35.4 [34.7, 36.1] | 4.9 [4.7, 5.2] | 2.2 [2.1, 2.4] |
| 2014 | 50.6 [49.9, 51.4] | 35.5 [34.8, 36.1] | 4.9 [4.7, 5.2] | 2.3 [2.1, 2.5] |
| 2015 | 51.3 [50.5, 52.0] | 35.7 [35.1, 36.4] | 4.8 [4.6, 5.1] | 1.9 [1.8, 2.1] |
| 2016 | 53.3 [52.5, 54.0] | 39.0 [38.3, 39.7] | 5.1 [4.9, 5.4] | 0.8 [0.7, 0.9] |
| 2017 | 57.0 [56.2, 57.8] | 42.4 [41.8, 43.1] | 5.0 [4.8, 5.2] | 0.9 [0.8, 1.0] |
| 2018 | 58.7 [57.9, 59.5] | 44.2 [43.5, 44.9] | 5.5 [5.2, 5.7] | 0.7 [0.6, 0.8] |
| 2019 | 57.7 [56.9, 58.5] | 43.1 [42.4, 43.8] | 5.3 [5.0, 5.5] | 0.9 [0.8, 1.0] |
| 2020 | 63.7 [62.9, 64.5] | 49.3 [48.6, 50.0] | 5.0 [4.7, 5.2] | 1.1 [1.0, 1.2] |
| 2021 | 67.7 [66.9, 68.5] | 53.6 [52.8, 54.3] | 6.4 [6.1, 6.6] | 1.1 [1.0, 1.2] |
| 2022 | 69.0 [68.1, 69.8] | 53.9 [53.2, 54.7] | 5.2 [5.0, 5.5] | 1.0 [0.9, 1.1] |
| 2023 | 64.9 [64.1, 65.7] | 51.4 [50.7, 52.1] | 5.1 [4.9, 5.3] | 1.0 [0.9, 1.1] |

Table A2-1: Number of deaths due to leading causes, males, Canada, from 2000 to 2023

| Year | Falls | Poisonings | Land transport incidents | Suffocation | Drowning | Smoke/fire/flames | Struck by/against | Suicide | Homicide |
| --- | --- | --- | --- | --- | --- | --- | --- | --- | --- |
| 2000 | 835 | 681 | 2086 | 252 | 287 | 150 | 104 | 2829 | 350 |
| 2001 | 887 | 682 | 2039 | 256 | 309 | 148 | 110 | 2911 | 355 |
| 2002 | 931 | 705 | 2117 | 283 | 305 | 141 | 82 | 2911 | 331 |
| 2003 | 1031 | 621 | 2077 | 239 | 297 | 121 | 73 | 2919 | 341 |
| 2004 | 1079 | 684 | 2065 | 236 | 292 | 140 | 78 | 2748 | 397 |
| 2005 | 1194 | 728 | 2192 | 224 | 276 | 136 | 101 | 2869 | 461 |
| 2006 | 1272 | 801 | 2115 | 239 | 305 | 131 | 86 | 2733 | 409 |
| 2007 | 1321 | 957 | 2175 | 281 | 274 | 141 | 91 | 2760 | 418 |
| 2008 | 1490 | 932 | 1935 | 256 | 299 | 162 | 95 | 2787 | 463 |
| 2009 | 1761 | 1025 | 1773 | 220 | 269 | 137 | 65 | 3020 | 437 |
| 2010 | 1921 | 1052 | 1800 | 257 | 309 | 144 | 77 | 3000 | 396 |
| 2011 | 2021 | 1133 | 1691 | 320 | 284 | 118 | 75 | 2942 | 398 |
| 2012 | 2048 | 1194 | 1699 | 288 | 304 | 112 | 83 | 3030 | 372 |
| 2013 | 2209 | 1258 | 1556 | 263 | 282 | 136 | 81 | 3046 | 327 |
| 2014 | 2296 | 1437 | 1518 | 252 | 266 | 117 | 73 | 3188 | 338 |
| 2015 | 2221 | 1729 | 1586 | 256 | 267 | 126 | 80 | 3330 | 356 |
| 2016 | 2360 | 2556 | 1577 | 265 | 259 | 144 | 79 | 3283 | 367 |
| 2017 | 2508 | 3634 | 1483 | 297 | 315 | 117 | 69 | 3338 | 414 |
| 2018 | 2640 | 3628 | 1557 | 280 | 279 | 131 | 93 | 3456 | 390 |
| 2019 | 2972 | 3220 | 1380 | 311 | 256 | 134 | 82 | 3507 | 421 |
| 2020 | 3180 | 5049 | 1388 | 307 | 349 | 121 | 88 | 3364 | 456 |
| 2021 | 3354 | 5700 | 1363 | 343 | 306 | 165 | 79 | 3399 | 390 |
| 2022 | 3611 | 5582 | 1522 | 378 | 284 | 168 | 69 | 3665 | 384 |
| 2023 | 3648 | 5444 | 1324 | 387 | 276 | 121 | 68 | 3516 | 264 |

Table A2-2: Number of deaths from all causes, all unintentional causes, other unintentional causes and undetermined causes, males, Canada, from 2000 to 2023

| Year | All causes | All unintentional causes | Other unintentional causes | Undetermined causes |
| --- | --- | --- | --- | --- |
| 2000 | 8790 | 5317 | 922 | 294 |
| 2001 | 8913 | 5365 | 934 | 282 |
| 2002 | 9109 | 5570 | 1006 | 297 |
| 2003 | 9191 | 5496 | 1037 | 435 |
| 2004 | 8989 | 5437 | 863 | 407 |
| 2005 | 9672 | 5876 | 1025 | 466 |
| 2006 | 9426 | 5895 | 946 | 389 |
| 2007 | 9731 | 6112 | 872 | 441 |
| 2008 | 9813 | 6106 | 937 | 457 |
| 2009 | 9943 | 6058 | 808 | 428 |
| 2010 | 10218 | 6394 | 834 | 428 |
| 2011 | 10268 | 6469 | 827 | 459 |
| 2012 | 10394 | 6509 | 781 | 483 |
| 2013 | 10504 | 6625 | 840 | 506 |
| 2014 | 10862 | 6779 | 820 | 557 |
| 2015 | 11263 | 7091 | 826 | 486 |
| 2016 | 11989 | 8144 | 904 | 195 |
| 2017 | 13258 | 9309 | 886 | 197 |
| 2018 | 13672 | 9650 | 1042 | 176 |
| 2019 | 13493 | 9351 | 996 | 214 |
| 2020 | 15536 | 11459 | 977 | 257 |
| 2021 | 16638 | 12554 | 1244 | 295 |
| 2022 | 16972 | 12678 | 1064 | 245 |
| 2023 | 16335 | 12295 | 1027 | 260 |

Table A2-3: Crude mortality rates (per 100 000 population) due to leading causes, males, Canada, from 2000 to 2023 (with 95% confidence intervals)

| Year | Falls | Poisonings | Land transport incidents | Suffocation | Drowning | Smoke/fire/ flames | Struck by/against | Suicide | Homicide |
| --- | --- | --- | --- | --- | --- | --- | --- | --- | --- |
| 2000 | 5.5 [5.1, 5.9] | 4.5 [4.1, 4.8] | 13.7 [13.1, 14.3] | 1.7 [1.5, 1.9] | 1.9 [1.7, 2.1] | 1.0 [0.8, 1.1] | 0.7 [0.6, 0.8] | 18.6 [17.9, 19.3] | 2.3 [2.1, 2.5] |
| 2001 | 5.8 [5.4, 6.2] | 4.4 [4.1, 4.8] | 13.3 [12.7, 13.8] | 1.7 [1.5, 1.9] | 2.0 [1.8, 2.2] | 1.0 [0.8, 1.1] | 0.7 [0.6, 0.8] | 18.9 [18.3, 19.6] | 2.3 [2.1, 2.6] |
| 2002 | 6.0 [5.6, 6.4] | 4.5 [4.2, 4.9] | 13.6 [13.0, 14.2] | 1.8 [1.6, 2.0] | 2.0 [1.7, 2.2] | 0.9 [0.8, 1.1] | 0.5 [0.4, 0.6] | 18.7 [18.1, 19.4] | 2.1 [1.9, 2.4] |
| 2003 | 6.6 [6.2, 7.0] | 4.0 [3.6, 4.3] | 13.2 [12.7, 13.8] | 1.5 [1.3, 1.7] | 1.9 [1.7, 2.1] | 0.8 [0.6, 0.9] | 0.5 [0.4, 0.6] | 18.6 [17.9, 19.3] | 2.2 [1.9, 2.4] |
| 2004 | 6.8 [6.4, 7.2] | 4.3 [4.0, 4.6] | 13.0 [12.5, 13.6] | 1.5 [1.3, 1.7] | 1.8 [1.6, 2.1] | 0.9 [0.7, 1.0] | 0.5 [0.4, 0.6] | 17.4 [16.7, 18.0] | 2.5 [2.3, 2.8] |
| 2005 | 7.5 [7.0, 7.9] | 4.6 [4.2, 4.9] | 13.7 [13.1, 14.3] | 1.4 [1.2, 1.6] | 1.7 [1.5, 1.9] | 0.9 [0.7, 1.0] | 0.6 [0.5, 0.8] | 18.0 [17.3, 18.6] | 2.9 [2.6, 3.1] |
| 2006 | 7.9 [7.4, 8.3] | 5.0 [4.6, 5.3] | 13.1 [12.5, 13.7] | 1.5 [1.3, 1.7] | 1.9 [1.7, 2.1] | 0.8 [0.7, 1.0] | 0.5 [0.4, 0.6] | 16.9 [16.3, 17.6] | 2.5 [2.3, 2.8] |
| 2007 | 8.1 [7.7, 8.5] | 5.9 [5.5, 6.2] | 13.3 [12.8, 13.9] | 1.7 [1.5, 1.9] | 1.7 [1.5, 1.9] | 0.9 [0.7, 1.0] | 0.6 [0.4, 0.7] | 16.9 [16.3, 17.6] | 2.6 [2.3, 2.8] |
| 2008 | 9.0 [8.6, 9.5] | 5.7 [5.3, 6.0] | 11.7 [11.2, 12.3] | 1.6 [1.4, 1.7] | 1.8 [1.6, 2.0] | 1.0 [0.8, 1.1] | 0.6 [0.5, 0.7] | 16.9 [16.3, 17.5] | 2.8 [2.6, 3.1] |
| 2009 | 10.6 [10.1, 11.1] | 6.2 [5.8, 6.5] | 10.6 [10.1, 11.1] | 1.3 [1.1, 1.5] | 1.6 [1.4, 1.8] | 0.8 [0.7, 1.0] | 0.4 [0.3, 0.5] | 18.1 [17.5, 18.8] | 2.6 [2.4, 2.9] |
| 2010 | 11.4 [10.9, 11.9] | 6.2 [5.9, 6.6] | 10.7 [10.2, 11.2] | 1.5 [1.3, 1.7] | 1.8 [1.6, 2.0] | 0.9 [0.7, 1.0] | 0.5 [0.4, 0.6] | 17.8 [17.2, 18.4] | 2.4 [2.1, 2.6] |
| 2011 | 11.9 [11.4, 12.4] | 6.7 [6.3, 7.0] | 9.9 [9.5, 10.4] | 1.9 [1.7, 2.1] | 1.7 [1.5, 1.9] | 0.7 [0.6, 0.8] | 0.4 [0.3, 0.5] | 17.3 [16.7, 17.9] | 2.3 [2.1, 2.6] |
| 2012 | 11.9 [11.4, 12.4] | 6.9 [6.5, 7.3] | 9.9 [9.4, 10.3] | 1.7 [1.5, 1.9] | 1.8 [1.6, 2.0] | 0.7 [0.5, 0.8] | 0.5 [0.4, 0.6] | 17.6 [17.0, 18.2] | 2.2 [1.9, 2.4] |
| 2013 | 12.7 [12.2, 13.2] | 7.2 [6.8, 7.6] | 8.9 [8.5, 9.4] | 1.5 [1.3, 1.7] | 1.6 [1.4, 1.8] | 0.8 [0.7, 0.9] | 0.5 [0.4, 0.6] | 17.5 [16.9, 18.1] | 1.9 [1.7, 2.1] |
| 2014 | 13.1 [12.5, 13.6] | 8.2 [7.8, 8.6] | 8.6 [8.2, 9.1] | 1.4 [1.3, 1.6] | 1.5 [1.3, 1.7] | 0.7 [0.5, 0.8] | 0.4 [0.3, 0.5] | 18.1 [17.5, 18.8] | 1.9 [1.7, 2.1] |
| 2015 | 12.5 [12.0, 13.1] | 9.8 [9.3, 10.2] | 9.0 [8.5, 9.4] | 1.4 [1.3, 1.6] | 1.5 [1.3, 1.7] | 0.7 [0.6, 0.8] | 0.5 [0.4, 0.6] | 18.8 [18.2, 19.4] | 2.0 [1.8, 2.2] |
| 2016 | 13.2 [12.6, 13.7] | 14.3 [13.7, 14.8] | 8.8 [8.4, 9.2] | 1.5 [1.3, 1.7] | 1.4 [1.3, 1.6] | 0.8 [0.7, 0.9] | 0.4 [0.3, 0.5] | 18.3 [17.7, 19.0] | 2.0 [1.8, 2.3] |
| 2017 | 13.8 [13.3, 14.4] | 20.0 [19.4, 20.7] | 8.2 [7.8, 8.6] | 1.6 [1.5, 1.8] | 1.7 [1.5, 1.9] | 0.6 [0.5, 0.8] | 0.4 [0.3, 0.5] | 18.4 [17.8, 19.0] | 2.3 [2.1, 2.5] |
| 2018 | 14.3 [13.8, 14.9] | 19.7 [19.0, 20.3] | 8.5 [8.0, 8.9] | 1.5 [1.3, 1.7] | 1.5 [1.3, 1.7] | 0.7 [0.6, 0.8] | 0.5 [0.4, 0.6] | 18.8 [18.1, 19.4] | 2.1 [1.9, 2.3] |
| 2019 | 15.9 [15.3, 16.5] | 17.2 [16.6, 17.8] | 7.4 [7.0, 7.8] | 1.7 [1.5, 1.8] | 1.4 [1.2, 1.5] | 0.7 [0.6, 0.8] | 0.4 [0.3, 0.5] | 18.7 [18.1, 19.4] | 2.2 [2.0, 2.5] |
| 2020 | 16.8 [16.2, 17.4] | 26.7 [25.9, 27.4] | 7.3 [6.9, 7.7] | 1.6 [1.4, 1.8] | 1.8 [1.7, 2.0] | 0.6 [0.5, 0.8] | 0.5 [0.4, 0.6] | 17.8 [17.2, 18.4] | 2.4 [2.2, 2.6] |
| 2021 | 17.6 [17.0, 18.2] | 29.9 [29.2, 30.7] | 7.2 [6.8, 7.5] | 1.8 [1.6, 2.0] | 1.6 [1.4, 1.8] | 0.9 [0.7, 1.0] | 0.4 [0.3, 0.5] | 17.8 [17.2, 18.4] | 2.0 [1.8, 2.3] |
| 2022 | 18.6 [18.0, 19.2] | 28.8 [28.0, 29.5] | 7.8 [7.4, 8.2] | 1.9 [1.8, 2.1] | 1.5 [1.3, 1.6] | 0.9 [0.7, 1.0] | 0.4 [0.3, 0.4] | 18.9 [18.3, 19.5] | 2.0 [1.8, 2.2] |
| 2023 | 18.3 [17.7, 18.8] | 27.2 [26.5, 28.0] | 6.6 [6.3, 7.0] | 1.9 [1.7, 2.1] | 1.4 [1.2, 1.5] | 0.6 [0.5, 0.7] | 0.3 [0.3, 0.4] | 17.6 [17.0, 18.2] | 1.3 [1.2, 1.5] |

Table A2-4: Crude mortality rates (per 100 000 population) from all causes, all unintentional causes, other unintentional causes and undetermined causes, males, Canada, from 2000 to 2023 (with 95% confidence intervals)

| Year | All causes | All unintentional causes | Other unintentional causes | Undetermined causes |
| --- | --- | --- | --- | --- |
| 2000 | 57.9 [56.6, 59.1] | 35.0 [34.1, 35.9] | 6.1 [5.7, 6.5] | 1.9 [1.7, 2.2] |
| 2001 | 58.0 [56.8, 59.2] | 34.9 [34.0, 35.8] | 6.1 [5.7, 6.5] | 1.8 [1.6, 2.0] |
| 2002 | 58.6 [57.4, 59.8] | 35.8 [34.9, 36.8] | 6.5 [6.1, 6.9] | 1.9 [1.7, 2.1] |
| 2003 | 58.6 [57.4, 59.8] | 35.1 [34.1, 36.0] | 6.6 [6.2, 7.0] | 2.8 [2.5, 3.0] |
| 2004 | 56.8 [55.6, 58.0] | 34.4 [33.4, 35.3] | 5.5 [5.1, 5.8] | 2.6 [2.3, 2.8] |
| 2005 | 60.5 [59.3, 61.7] | 36.8 [35.8, 37.7] | 6.4 [6.0, 6.8] | 2.9 [2.7, 3.2] |
| 2006 | 58.4 [57.2, 59.6] | 36.5 [35.6, 37.4] | 5.9 [5.5, 6.2] | 2.4 [2.2, 2.6] |
| 2007 | 59.7 [58.5, 60.9] | 37.5 [36.6, 38.4] | 5.4 [5.0, 5.7] | 2.7 [2.5, 3.0] |
| 2008 | 59.6 [58.4, 60.7] | 37.1 [36.1, 38.0] | 5.7 [5.3, 6.1] | 2.8 [2.5, 3.0] |
| 2009 | 59.7 [58.5, 60.8] | 36.4 [35.4, 37.3] | 4.8 [4.5, 5.2] | 2.6 [2.3, 2.8] |
| 2010 | 60.6 [59.5, 61.8] | 38.0 [37.0, 38.9] | 5.0 [4.6, 5.3] | 2.5 [2.3, 2.8] |
| 2011 | 60.4 [59.2, 61.5] | 38.0 [37.1, 39.0] | 4.9 [4.5, 5.2] | 2.7 [2.5, 2.9] |
| 2012 | 60.4 [59.2, 61.6] | 37.8 [36.9, 38.7] | 4.5 [4.2, 4.9] | 2.8 [2.6, 3.1] |
| 2013 | 60.4 [59.2, 61.5] | 38.1 [37.2, 39.0] | 4.8 [4.5, 5.2] | 2.9 [2.7, 3.2] |
| 2014 | 61.8 [60.6, 63.0] | 38.6 [37.7, 39.5] | 4.7 [4.3, 5.0] | 3.2 [2.9, 3.4] |
| 2015 | 63.6 [62.4, 64.8] | 40.0 [39.1, 41.0] | 4.7 [4.3, 5.0] | 2.7 [2.5, 3.0] |
| 2016 | 66.9 [65.7, 68.1] | 45.5 [44.5, 46.4] | 5.0 [4.7, 5.4] | 1.1 [0.9, 1.2] |
| 2017 | 73.1 [71.8, 74.3] | 51.3 [50.3, 52.4] | 4.9 [4.6, 5.2] | 1.1 [0.9, 1.2] |
| 2018 | 74.2 [73.0, 75.4] | 52.4 [51.3, 53.4] | 5.7 [5.3, 6.0] | 1.0 [0.8, 1.1] |
| 2019 | 72.1 [70.9, 73.3] | 50.0 [49.0, 51.0] | 5.3 [5.0, 5.7] | 1.1 [1.0, 1.3] |
| 2020 | 82.1 [80.8, 83.4] | 60.5 [59.4, 61.6] | 5.2 [4.8, 5.5] | 1.4 [1.2, 1.5] |
| 2021 | 87.4 [86.0, 88.7] | 65.9 [64.8, 67.1] | 6.5 [6.2, 6.9] | 1.5 [1.4, 1.7] |
| 2022 | 87.5 [86.2, 88.8] | 65.3 [64.2, 66.5] | 5.5 [5.2, 5.8] | 1.3 [1.1, 1.4] |
| 2023 | 81.7 [80.5, 83.0] | 61.5 [60.4, 62.6] | 5.1 [4.8, 5.5] | 1.3 [1.1, 1.5] |

Table A2-5: Age-standardized mortality rates (per 100 000 population) due to leading causes, males, Canada, from 2000 to 2023 (with 95% confidence intervals)

| Year | Falls | Poisonings | Land transport incidents | Suffocation | Drowning | Smoke/fire/ flames | Struck by/against | Suicide | Homicide |
| --- | --- | --- | --- | --- | --- | --- | --- | --- | --- |
| 2000 | 10.7 [9.9, 11.5] | 4.3 [4.0, 4.7] | 14.6 [13.9, 15.3] | 2.4 [2.1, 2.7] | 1.9 [1.7, 2.1] | 1.2 [1.0, 1.4] | 0.8 [0.6, 1.0] | 19.2 [18.5, 20.0] | 2.3 [2.0, 2.5] |
| 2001 | 11.1 [10.3, 12.0] | 4.2 [3.9, 4.6] | 13.9 [13.3, 14.6] | 2.3 [2.0, 2.7] | 2.0 [1.8, 2.3] | 1.2 [1.0, 1.4] | 0.8 [0.6, 0.9] | 19.5 [18.8, 20.3] | 2.1 [1.9, 2.4] |
| 2002 | 10.8 [10.0, 11.6] | 4.3 [4.0, 4.7] | 14.3 [13.7, 15.0] | 2.6 [2.2, 2.9] | 2.0 [1.8, 2.3] | 1.0 [0.8, 1.2] | 0.6 [0.5, 0.7] | 19.6 [18.9, 20.3] | 2.1 [1.8, 2.3] |
| 2003 | 11.8 [11.0, 12.6] | 3.9 [3.6, 4.2] | 13.7 [13.1, 14.3] | 2.3 [2.0, 2.6] | 1.9 [1.7, 2.1] | 0.9 [0.8, 1.1] | 0.5 [0.4, 0.7] | 19.2 [18.5, 20.0] | 2.1 [1.9, 2.4] |
| 2004 | 12.1 [11.3, 12.9] | 4.3 [3.9, 4.6] | 13.7 [13.1, 14.3] | 2.0 [1.7, 2.3] | 1.9 [1.7, 2.2] | 1.0 [0.8, 1.2] | 0.5 [0.4, 0.6] | 17.8 [17.1, 18.5] | 2.4 [2.2, 2.7] |
| 2005 | 12.7 [12.0, 13.6] | 4.3 [4.0, 4.6] | 14.2 [13.5, 14.8] | 1.9 [1.7, 2.2] | 1.7 [1.5, 2.0] | 1.1 [0.9, 1.3] | 0.7 [0.5, 0.8] | 18.4 [17.7, 19.1] | 2.8 [2.5, 3.0] |
| 2006 | 13.6 [12.8, 14.4] | 4.7 [4.4, 5.1] | 13.4 [12.8, 14.0] | 2.0 [1.7, 2.3] | 1.9 [1.7, 2.2] | 0.9 [0.7, 1.0] | 0.6 [0.5, 0.8] | 17.5 [16.8, 18.1] | 2.4 [2.2, 2.7] |
| 2007 | 13.6 [12.8, 14.4] | 5.7 [5.3, 6.0] | 13.6 [13.0, 14.2] | 2.4 [2.1, 2.7] | 1.8 [1.5, 2.0] | 1.0 [0.8, 1.2] | 0.6 [0.5, 0.8] | 17.3 [16.6, 17.9] | 2.4 [2.2, 2.7] |
| 2008 | 14.9 [14.1, 15.8] | 5.5 [5.2, 5.9] | 12.1 [11.6, 12.7] | 2.1 [1.9, 2.5] | 1.9 [1.7, 2.2] | 1.1 [0.9, 1.3] | 0.6 [0.5, 0.7] | 17.3 [16.7, 18.0] | 2.6 [2.4, 2.9] |
| 2009 | 17.3 [16.5, 18.2] | 5.9 [5.6, 6.3] | 10.9 [10.4, 11.5] | 1.9 [1.6, 2.1] | 1.6 [1.4, 1.8] | 1.0 [0.8, 1.1] | 0.4 [0.3, 0.6] | 18.4 [17.7, 19.1] | 2.5 [2.3, 2.8] |
| 2010 | 18.1 [17.3, 19.0] | 6.0 [5.6, 6.4] | 11.0 [10.5, 11.6] | 2.1 [1.8, 2.4] | 1.9 [1.6, 2.1] | 0.9 [0.8, 1.1] | 0.5 [0.4, 0.6] | 18.0 [17.4, 18.7] | 2.3 [2.0, 2.5] |
| 2011 | 18.4 [17.6, 19.3] | 6.4 [6.1, 6.8] | 10.3 [9.8, 10.8] | 2.6 [2.3, 2.9] | 1.7 [1.5, 1.9] | 0.8 [0.6, 0.9] | 0.5 [0.4, 0.6] | 17.5 [16.9, 18.1] | 2.3 [2.0, 2.5] |
| 2012 | 18.2 [17.4, 19.0] | 6.7 [6.3, 7.1] | 10.1 [9.6, 10.6] | 2.2 [1.9, 2.5] | 1.8 [1.6, 2.1] | 0.7 [0.6, 0.9] | 0.5 [0.4, 0.6] | 17.7 [17.1, 18.3] | 2.1 [1.9, 2.3] |
| 2013 | 18.8 [18.0, 19.6] | 7.0 [6.6, 7.4] | 9.2 [8.7, 9.6] | 1.9 [1.7, 2.1] | 1.7 [1.5, 1.9] | 0.9 [0.7, 1.0] | 0.5 [0.4, 0.6] | 17.7 [17.1, 18.4] | 1.8 [1.6, 2.1] |
| 2014 | 18.6 [17.8, 19.4] | 8.0 [7.6, 8.4] | 8.8 [8.4, 9.3] | 1.7 [1.5, 2.0] | 1.5 [1.4, 1.7] | 0.7 [0.6, 0.9] | 0.4 [0.3, 0.5] | 18.3 [17.7, 19.0] | 1.9 [1.7, 2.1] |
| 2015 | 17.5 [16.8, 18.3] | 9.5 [9.1, 10.0] | 9.2 [8.7, 9.6] | 1.7 [1.5, 1.9] | 1.5 [1.3, 1.7] | 0.8 [0.6, 0.9] | 0.5 [0.4, 0.6] | 18.9 [18.3, 19.6] | 2.0 [1.8, 2.2] |
| 2016 | 17.9 [17.2, 18.7] | 13.9 [13.4, 14.5] | 9.0 [8.6, 9.5] | 1.7 [1.5, 2.0] | 1.5 [1.3, 1.7] | 0.8 [0.7, 1.0] | 0.5 [0.4, 0.6] | 18.5 [17.8, 19.1] | 2.0 [1.8, 2.2] |
| 2017 | 18.5 [17.8, 19.3] | 19.7 [19.1, 20.3] | 8.3 [7.9, 8.8] | 1.9 [1.7, 2.1] | 1.8 [1.6, 2.0] | 0.7 [0.6, 0.8] | 0.4 [0.3, 0.5] | 18.5 [17.9, 19.2] | 2.2 [2.0, 2.5] |
| 2018 | 18.8 [18.1, 19.6] | 19.4 [18.8, 20.0] | 8.7 [8.2, 9.1] | 1.8 [1.6, 2.0] | 1.5 [1.4, 1.7] | 0.7 [0.6, 0.9] | 0.5 [0.4, 0.7] | 18.8 [18.2, 19.5] | 2.1 [1.9, 2.3] |
| 2019 | 20.7 [20.0, 21.5] | 17.0 [16.4, 17.6] | 7.5 [7.1, 7.9] | 2.0 [1.8, 2.2] | 1.4 [1.2, 1.6] | 0.8 [0.6, 0.9] | 0.5 [0.4, 0.6] | 18.9 [18.3, 19.5] | 2.2 [2.0, 2.4] |
| 2020 | 21.6 [20.8, 22.4] | 26.4 [25.7, 27.1] | 7.4 [7.0, 7.8] | 1.9 [1.6, 2.1] | 1.9 [1.7, 2.1] | 0.7 [0.6, 0.8] | 0.5 [0.4, 0.6] | 17.9 [17.3, 18.5] | 2.4 [2.2, 2.6] |
| 2021 | 21.9 [21.2, 22.7] | 29.7 [29.0, 30.5] | 7.3 [6.9, 7.6] | 2.1 [1.9, 2.3] | 1.6 [1.4, 1.8] | 0.9 [0.8, 1.0] | 0.4 [0.3, 0.5] | 17.9 [17.3, 18.6] | 2.0 [1.8, 2.2] |
| 2022 | 22.9 [22.2, 23.7] | 28.7 [27.9, 29.5] | 7.9 [7.5, 8.3] | 2.3 [2.0, 2.5] | 1.5 [1.3, 1.7] | 0.9 [0.8, 1.1] | 0.4 [0.3, 0.5] | 19.0 [18.4, 19.6] | 2.0 [1.8, 2.2] |
| 2023 | 22.4 [21.7, 23.2] | 27.2 [26.5, 27.9] | 6.7 [6.4, 7.1] | 2.2 [2.0, 2.5] | 1.4 [1.2, 1.6] | 0.6 [0.5, 0.7] | 0.4 [0.3, 0.5] | 17.7 [17.1, 18.3] | 1.3 [1.1, 1.5] |

Table A2-6: Age-standardized mortality rates (per 100 000 population) from all causes, all unintentional causes, other unintentional causes and undetermined causes, males, Canada, from 2000 to 2023 (with 95% confidence intervals)

| Year | All causes | All unintentional causes | Other unintentional causes | Undetermined causes |
| --- | --- | --- | --- | --- |
| 2000 | 67.5 [66.0, 69.1] | 46.7 [45.3, 48.2] | 11.0 [10.2, 11.8] | 2.0 [1.8, 2.3] |
| 2001 | 67.0 [65.5, 68.5] | 46.5 [45.0, 48.0] | 11.0 [10.2, 11.8] | 1.8 [1.6, 2.1] |
| 2002 | 68.0 [66.5, 69.5] | 47.1 [45.7, 48.6] | 11.5 [10.7, 12.4] | 1.9 [1.7, 2.1] |
| 2003 | 67.5 [66.1, 69.0] | 46.5 [45.1, 47.9] | 11.5 [10.7, 12.3] | 2.7 [2.4, 3.0] |
| 2004 | 64.7 [63.3, 66.1] | 44.6 [43.3, 45.9] | 9.1 [8.4, 9.8] | 2.5 [2.3, 2.8] |
| 2005 | 68.5 [67.1, 70.0] | 47.2 [45.9, 48.6] | 10.6 [9.9, 11.3] | 2.8 [2.6, 3.1] |
| 2006 | 65.8 [64.4, 67.2] | 46.3 [45.0, 47.6] | 9.2 [8.5, 9.8] | 2.3 [2.1, 2.5] |
| 2007 | 66.6 [65.3, 68.0] | 46.7 [45.4, 48.0] | 8.1 [7.5, 8.7] | 2.6 [2.3, 2.8] |
| 2008 | 66.6 [65.3, 68.0] | 46.8 [45.5, 48.1] | 8.4 [7.8, 9.0] | 2.7 [2.5, 3.0] |
| 2009 | 66.5 [65.2, 67.9] | 45.9 [44.6, 47.1] | 6.8 [6.3, 7.3] | 2.5 [2.2, 2.7] |
| 2010 | 67.5 [66.2, 68.9] | 47.4 [46.2, 48.7] | 6.9 [6.4, 7.4] | 2.5 [2.2, 2.7] |
| 2011 | 66.9 [65.6, 68.3] | 47.2 [46.0, 48.5] | 6.6 [6.1, 7.1] | 2.6 [2.3, 2.8] |
| 2012 | 66.1 [64.8, 67.4] | 46.2 [45.0, 47.4] | 6.0 [5.6, 6.5] | 2.8 [2.5, 3.0] |
| 2013 | 65.9 [64.7, 67.2] | 46.0 [44.8, 47.2] | 6.2 [5.7, 6.6] | 2.8 [2.6, 3.1] |
| 2014 | 66.9 [65.7, 68.2] | 45.8 [44.7, 47.0] | 6.0 [5.6, 6.5] | 3.1 [2.8, 3.4] |
| 2015 | 68.3 [67.0, 69.6] | 46.6 [45.4, 47.7] | 5.9 [5.5, 6.3] | 2.7 [2.5, 2.9] |
| 2016 | 71.4 [70.1, 72.7] | 51.7 [50.6, 52.9] | 6.3 [5.9, 6.7] | 1.1 [0.9, 1.2] |
| 2017 | 77.4 [76.1, 78.7] | 57.3 [56.1, 58.5] | 6.0 [5.6, 6.4] | 1.1 [0.9, 1.2] |
| 2018 | 78.5 [77.2, 79.8] | 58.3 [57.1, 59.5] | 6.8 [6.4, 7.2] | 1.0 [0.8, 1.1] |
| 2019 | 76.5 [75.2, 77.8] | 56.2 [55.0, 57.3] | 6.3 [5.9, 6.8] | 1.2 [1.0, 1.3] |
| 2020 | 86.1 [84.8, 87.5] | 66.4 [65.2, 67.6] | 6.1 [5.7, 6.5] | 1.4 [1.2, 1.5] |
| 2021 | 91.0 [89.6, 92.4] | 71.3 [70.1, 72.6] | 7.4 [7.0, 7.8] | 1.6 [1.4, 1.7] |
| 2022 | 91.0 [89.7, 92.4] | 70.8 [69.6, 72.1] | 6.2 [5.8, 6.6] | 1.3 [1.1, 1.4] |
| 2023 | 84.9 [83.6, 86.2] | 66.8 [65.6, 68.0] | 5.9 [5.5, 6.2] | 1.3 [1.2, 1.5] |

Table A3-1: Number of deaths due to leading causes, females, Canada, from 2000 to 2023

| Year | Falls | Poisonings | Land transport incidents | Suffocation | Drowning | Smoke/fire/flames | Struck by/against | Suicide | Homicide |
| --- | --- | --- | --- | --- | --- | --- | --- | --- | --- |
| 2000 | 735 | 282 | 913 | 186 | 62 | 92 | 8 | 814 | 134 |
| 2001 | 847 | 291 | 854 | 136 | 52 | 102 | 10 | 831 | 141 |
| 2002 | 848 | 325 | 943 | 164 | 72 | 83 | 5 | 814 | 159 |
| 2003 | 958 | 272 | 877 | 185 | 59 | 77 | 5 | 868 | 127 |
| 2004 | 1146 | 273 | 836 | 165 | 55 | 96 | 13 | 881 | 161 |
| 2005 | 1112 | 327 | 941 | 175 | 66 | 79 | 5 | 893 | 162 |
| 2006 | 1339 | 354 | 953 | 187 | 57 | 69 | 9 | 835 | 147 |
| 2007 | 1419 | 435 | 842 | 192 | 66 | 75 | 13 | 894 | 148 |
| 2008 | 1629 | 505 | 777 | 185 | 59 | 113 | 11 | 931 | 126 |
| 2009 | 1880 | 484 | 703 | 209 | 49 | 88 | 17 | 911 | 150 |
| 2010 | 2146 | 525 | 749 | 189 | 63 | 82 | 13 | 975 | 141 |
| 2011 | 2255 | 568 | 644 | 198 | 62 | 77 | 11 | 996 | 167 |
| 2012 | 2462 | 602 | 716 | 204 | 72 | 79 | 11 | 967 | 134 |
| 2013 | 2500 | 583 | 712 | 185 | 74 | 106 | 19 | 1011 | 135 |
| 2014 | 2602 | 653 | 604 | 181 | 62 | 111 | 21 | 1104 | 123 |
| 2015 | 2556 | 740 | 617 | 181 | 77 | 87 | 13 | 1156 | 154 |
| 2016 | 2586 | 1013 | 674 | 174 | 71 | 71 | 8 | 1167 | 108 |
| 2017 | 2724 | 1231 | 628 | 189 | 78 | 74 | 18 | 1140 | 150 |
| 2018 | 3125 | 1336 | 634 | 207 | 84 | 73 | 14 | 1139 | 132 |
| 2019 | 3368 | 1270 | 627 | 212 | 74 | 72 | 6 | 1114 | 125 |
| 2020 | 3652 | 1631 | 522 | 216 | 93 | 69 | 12 | 1159 | 126 |
| 2021 | 3845 | 1990 | 519 | 220 | 80 | 75 | 12 | 1107 | 107 |
| 2022 | 4509 | 1890 | 573 | 273 | 92 | 85 | 10 | 1310 | 115 |
| 2023 | 4397 | 2031 | 537 | 263 | 79 | 70 | 14 | 1203 | 90 |

Table A3-2: Number of deaths from all causes, all unintentional causes, other unintentional causes and undetermined causes, females, Canada, from 2000 to 2023

| Year | All causes | All unintentional causes | Other unintentional causes | Undetermined causes |
| --- | --- | --- | --- | --- |
| 2000 | 4373 | 3314 | 1036 | 111 |
| 2001 | 4344 | 3229 | 937 | 143 |
| 2002 | 4700 | 3579 | 1139 | 148 |
| 2003 | 4822 | 3612 | 1179 | 215 |
| 2004 | 4849 | 3573 | 989 | 234 |
| 2005 | 5005 | 3716 | 1011 | 234 |
| 2006 | 5146 | 3922 | 954 | 242 |
| 2007 | 5231 | 3961 | 919 | 228 |
| 2008 | 5422 | 4132 | 853 | 233 |
| 2009 | 5501 | 4180 | 750 | 260 |
| 2010 | 5951 | 4521 | 754 | 314 |
| 2011 | 5951 | 4542 | 727 | 246 |
| 2012 | 6252 | 4845 | 699 | 306 |
| 2013 | 6333 | 4898 | 719 | 289 |
| 2014 | 6509 | 5010 | 776 | 272 |
| 2015 | 6561 | 5039 | 768 | 212 |
| 2016 | 6815 | 5433 | 836 | 107 |
| 2017 | 7215 | 5794 | 852 | 131 |
| 2018 | 7736 | 6378 | 905 | 87 |
| 2019 | 7915 | 6559 | 930 | 117 |
| 2020 | 8500 | 7070 | 875 | 145 |
| 2021 | 9257 | 7927 | 1186 | 116 |
| 2022 | 9986 | 8419 | 987 | 142 |
| 2023 | 9829 | 8414 | 1023 | 122 |

Table A3-3: Crude mortality rates (per 100 000 population) due to leading causes, females, Canada, from 2000 to 2023 (with 95% confidence intervals)

| Year | Falls | Poisonings | Land transport incidents | Suffocation | Drowning | Smoke/fire/ flames | Struck by/against | Suicide | Homicide |
| --- | --- | --- | --- | --- | --- | --- | --- | --- | --- |
| 2000 | 4.7 [4.4, 5.1] | 1.8 [1.6, 2.0] | 5.9 [5.5, 6.3] | 1.2 [1.0, 1.4] | 0.4 [0.3, 0.5] | 0.6 [0.5, 0.7] | 0.1 [0.0, 0.1] | 5.3 [4.9, 5.6] | 0.9 [0.7, 1.0] |
| 2001 | 5.4 [5.0, 5.8] | 1.9 [1.6, 2.1] | 5.5 [5.1, 5.8] | 0.9 [0.7, 1.0] | 0.3 [0.2, 0.4] | 0.7 [0.5, 0.8] | 0.1 [0.0, 0.1] | 5.3 [4.9, 5.7] | 0.9 [0.8, 1.0] |
| 2002 | 5.4 [5.0, 5.7] | 2.1 [1.8, 2.3] | 6.0 [5.6, 6.3] | 1.0 [0.9, 1.2] | 0.5 [0.3, 0.6] | 0.5 [0.4, 0.6] | 0.0 [0.0, 0.1] | 5.1 [4.8, 5.5] | 1.0 [0.8, 1.2] |
| 2003 | 6.0 [5.6, 6.4] | 1.7 [1.5, 1.9] | 5.5 [5.1, 5.9] | 1.2 [1.0, 1.3] | 0.4 [0.3, 0.5] | 0.5 [0.4, 0.6] | 0.0 [0.0, 0.1] | 5.4 [5.1, 5.8] | 0.8 [0.7, 0.9] |
| 2004 | 7.1 [6.7, 7.5] | 1.7 [1.5, 1.9] | 5.2 [4.8, 5.5] | 1.0 [0.9, 1.2] | 0.3 [0.3, 0.4] | 0.6 [0.5, 0.7] | 0.1 [0.0, 0.1] | 5.5 [5.1, 5.8] | 1.0 [0.8, 1.2] |
| 2005 | 6.8 [6.4, 7.2] | 2.0 [1.8, 2.2] | 5.8 [5.4, 6.2] | 1.1 [0.9, 1.2] | 0.4 [0.3, 0.5] | 0.5 [0.4, 0.6] | 0.0 [0.0, 0.1] | 5.5 [5.1, 5.9] | 1.0 [0.8, 1.1] |
| 2006 | 8.2 [7.7, 8.6] | 2.2 [1.9, 2.4] | 5.8 [5.4, 6.2] | 1.1 [1.0, 1.3] | 0.3 [0.3, 0.4] | 0.4 [0.3, 0.5] | 0.1 [0.0, 0.1] | 5.1 [4.7, 5.4] | 0.9 [0.8, 1.0] |
| 2007 | 8.6 [8.1, 9.0] | 2.6 [2.4, 2.9] | 5.1 [4.7, 5.4] | 1.2 [1.0, 1.3] | 0.4 [0.3, 0.5] | 0.5 [0.3, 0.6] | 0.1 [0.0, 0.1] | 5.4 [5.0, 5.7] | 0.9 [0.7, 1.0] |
| 2008 | 9.7 [9.2, 10.2] | 3.0 [2.7, 3.3] | 4.6 [4.3, 5.0] | 1.1 [0.9, 1.3] | 0.4 [0.3, 0.4] | 0.7 [0.5, 0.8] | 0.1 [0.0, 0.1] | 5.6 [5.2, 5.9] | 0.8 [0.6, 0.9] |
| 2009 | 11.1 [10.6, 11.6] | 2.9 [2.6, 3.1] | 4.1 [3.8, 4.4] | 1.2 [1.1, 1.4] | 0.3 [0.2, 0.4] | 0.5 [0.4, 0.6] | 0.1 [0.1, 0.1] | 5.4 [5.0, 5.7] | 0.9 [0.7, 1.0] |
| 2010 | 12.5 [12.0, 13.0] | 3.1 [2.8, 3.3] | 4.4 [4.1, 4.7] | 1.1 [0.9, 1.3] | 0.4 [0.3, 0.5] | 0.5 [0.4, 0.6] | 0.1 [0.0, 0.1] | 5.7 [5.3, 6.0] | 0.8 [0.7, 1.0] |
| 2011 | 13.0 [12.5, 13.6] | 3.3 [3.0, 3.5] | 3.7 [3.4, 4.0] | 1.1 [1.0, 1.3] | 0.4 [0.3, 0.4] | 0.4 [0.3, 0.5] | 0.1 [0.0, 0.1] | 5.7 [5.4, 6.1] | 1.0 [0.8, 1.1] |
| 2012 | 14.1 [13.5, 14.6] | 3.4 [3.2, 3.7] | 4.1 [3.8, 4.4] | 1.2 [1.0, 1.3] | 0.4 [0.3, 0.5] | 0.5 [0.4, 0.6] | 0.1 [0.0, 0.1] | 5.5 [5.2, 5.9] | 0.8 [0.6, 0.9] |
| 2013 | 14.1 [13.6, 14.7] | 3.3 [3.0, 3.6] | 4.0 [3.7, 4.3] | 1.0 [0.9, 1.2] | 0.4 [0.3, 0.5] | 0.6 [0.5, 0.7] | 0.1 [0.1, 0.2] | 5.7 [5.4, 6.1] | 0.8 [0.6, 0.9] |
| 2014 | 14.6 [14.0, 15.1] | 3.7 [3.4, 3.9] | 3.4 [3.1, 3.7] | 1.0 [0.9, 1.2] | 0.3 [0.3, 0.4] | 0.6 [0.5, 0.7] | 0.1 [0.1, 0.2] | 6.2 [5.8, 6.5] | 0.7 [0.6, 0.8] |
| 2015 | 14.2 [13.7, 14.8] | 4.1 [3.8, 4.4] | 3.4 [3.2, 3.7] | 1.0 [0.9, 1.2] | 0.4 [0.3, 0.5] | 0.5 [0.4, 0.6] | 0.1 [0.0, 0.1] | 6.4 [6.1, 6.8] | 0.9 [0.7, 1.0] |
| 2016 | 14.2 [13.7, 14.8] | 5.6 [5.2, 5.9] | 3.7 [3.4, 4.0] | 1.0 [0.8, 1.1] | 0.4 [0.3, 0.5] | 0.4 [0.3, 0.5] | 0.0 [0.0, 0.1] | 6.4 [6.0, 6.8] | 0.6 [0.5, 0.7] |
| 2017 | 14.8 [14.2, 15.4] | 6.7 [6.3, 7.1] | 3.4 [3.1, 3.7] | 1.0 [0.9, 1.2] | 0.4 [0.3, 0.5] | 0.4 [0.3, 0.5] | 0.1 [0.1, 0.1] | 6.2 [5.8, 6.6] | 0.8 [0.7, 0.9] |
| 2018 | 16.8 [16.2, 17.3] | 7.2 [6.8, 7.5] | 3.4 [3.1, 3.7] | 1.1 [1.0, 1.3] | 0.5 [0.4, 0.5] | 0.4 [0.3, 0.5] | 0.1 [0.0, 0.1] | 6.1 [5.8, 6.5] | 0.7 [0.6, 0.8] |
| 2019 | 17.8 [17.2, 18.4] | 6.7 [6.3, 7.1] | 3.3 [3.1, 3.6] | 1.1 [1.0, 1.3] | 0.4 [0.3, 0.5] | 0.4 [0.3, 0.5] | 0.0 [0.0, 0.1] | 5.9 [5.5, 6.2] | 0.7 [0.5, 0.8] |
| 2020 | 19.1 [18.5, 19.7] | 8.5 [8.1, 9.0] | 2.7 [2.5, 3.0] | 1.1 [1.0, 1.3] | 0.5 [0.4, 0.6] | 0.4 [0.3, 0.4] | 0.1 [0.0, 0.1] | 6.1 [5.7, 6.4] | 0.7 [0.5, 0.8] |
| 2021 | 20.0 [19.4, 20.7] | 10.4 [9.9, 10.8] | 2.7 [2.5, 2.9] | 1.1 [1.0, 1.3] | 0.4 [0.3, 0.5] | 0.4 [0.3, 0.5] | 0.1 [0.0, 0.1] | 5.8 [5.4, 6.1] | 0.6 [0.5, 0.7] |
| 2022 | 23.1 [22.4, 23.7] | 9.7 [9.2, 10.1] | 2.9 [2.7, 3.2] | 1.4 [1.2, 1.6] | 0.5 [0.4, 0.6] | 0.4 [0.3, 0.5] | 0.1 [0.0, 0.1] | 6.7 [6.3, 7.1] | 0.6 [0.5, 0.7] |
| 2023 | 21.9 [21.3, 22.6] | 10.1 [9.7, 10.6] | 2.7 [2.5, 2.9] | 1.3 [1.2, 1.5] | 0.4 [0.3, 0.5] | 0.3 [0.3, 0.4] | 0.1 [0.0, 0.1] | 6.0 [5.7, 6.3] | 0.4 [0.4, 0.5] |

Table A3-4: Crude mortality rates (per 100 000 population) from all causes, all unintentional causes, other unintentional causes and undetermined causes, females, Canada, from 2000 to 2023 (with 95% confidence intervals)

| Year | All causes | All unintentional causes | Other unintentional causes | Undetermined causes |
| --- | --- | --- | --- | --- |
| 2000 | 28.2 [27.4, 29.1] | 21.4 [20.7, 22.1] | 6.7 [6.3, 7.1] | 0.7 [0.6, 0.8] |
| 2001 | 27.7 [26.9, 28.6] | 20.6 [19.9, 21.3] | 6.0 [5.6, 6.4] | 0.9 [0.8, 1.1] |
| 2002 | 29.7 [28.9, 30.6] | 22.6 [21.9, 23.4] | 7.2 [6.8, 7.6] | 0.9 [0.8, 1.1] |
| 2003 | 30.2 [29.4, 31.1] | 22.6 [21.9, 23.4] | 7.4 [7.0, 7.8] | 1.3 [1.2, 1.5] |
| 2004 | 30.1 [29.2, 30.9] | 22.2 [21.4, 22.9] | 6.1 [5.8, 6.5] | 1.5 [1.3, 1.6] |
| 2005 | 30.8 [29.9, 31.6] | 22.8 [22.1, 23.6] | 6.2 [5.8, 6.6] | 1.4 [1.3, 1.6] |
| 2006 | 31.3 [30.5, 32.2] | 23.9 [23.1, 24.6] | 5.8 [5.4, 6.2] | 1.5 [1.3, 1.7] |
| 2007 | 31.5 [30.7, 32.4] | 23.9 [23.1, 24.6] | 5.5 [5.2, 5.9] | 1.4 [1.2, 1.6] |
| 2008 | 32.3 [31.5, 33.2] | 24.6 [23.9, 25.4] | 5.1 [4.7, 5.4] | 1.4 [1.2, 1.6] |
| 2009 | 32.4 [31.6, 33.3] | 24.6 [23.9, 25.4] | 4.4 [4.1, 4.7] | 1.5 [1.3, 1.7] |
| 2010 | 34.7 [33.8, 35.6] | 26.4 [25.6, 27.1] | 4.4 [4.1, 4.7] | 1.8 [1.6, 2.0] |
| 2011 | 34.3 [33.5, 35.2] | 26.2 [25.5, 27.0] | 4.2 [3.9, 4.5] | 1.4 [1.2, 1.6] |
| 2012 | 35.7 [34.8, 36.6] | 27.7 [26.9, 28.5] | 4.0 [3.7, 4.3] | 1.7 [1.6, 1.9] |
| 2013 | 35.8 [34.9, 36.7] | 27.7 [26.9, 28.5] | 4.1 [3.8, 4.4] | 1.6 [1.4, 1.8] |
| 2014 | 36.4 [35.6, 37.3] | 28.1 [27.3, 28.8] | 4.3 [4.0, 4.7] | 1.5 [1.3, 1.7] |
| 2015 | 36.5 [35.6, 37.3] | 28.0 [27.2, 28.8] | 4.3 [4.0, 4.6] | 1.2 [1.0, 1.3] |
| 2016 | 37.5 [36.6, 38.3] | 29.9 [29.1, 30.7] | 4.6 [4.3, 4.9] | 0.6 [0.5, 0.7] |
| 2017 | 39.2 [38.3, 40.1] | 31.5 [30.7, 32.3] | 4.6 [4.3, 4.9] | 0.7 [0.6, 0.8] |
| 2018 | 41.5 [40.6, 42.4] | 34.2 [33.4, 35.0] | 4.9 [4.5, 5.2] | 0.5 [0.4, 0.6] |
| 2019 | 41.9 [40.9, 42.8] | 34.7 [33.9, 35.5] | 4.9 [4.6, 5.2] | 0.6 [0.5, 0.7] |
| 2020 | 44.5 [43.6, 45.4] | 37.0 [36.2, 37.9] | 4.6 [4.3, 4.9] | 0.8 [0.6, 0.9] |
| 2021 | 48.2 [47.2, 49.2] | 41.3 [40.4, 42.2] | 6.2 [5.8, 6.5] | 0.6 [0.5, 0.7] |
| 2022 | 51.1 [50.1, 52.1] | 43.1 [42.2, 44.0] | 5.0 [4.7, 5.4] | 0.7 [0.6, 0.8] |
| 2023 | 49.0 [48.0, 50.0] | 41.9 [41.0, 42.8] | 5.1 [4.8, 5.4] | 0.6 [0.5, 0.7] |

Table A3-5: Age-standardized mortality rates (per 100 000 population) due to leading causes, females, Canada, from 2000 to 2023 (with 95% confidence intervals)

| Year | Falls | Poisonings | Land transport incidents | Suffocation | Drowning | Smoke/fire/ flames | Struck by/against | Suicide | Homicide |
| --- | --- | --- | --- | --- | --- | --- | --- | --- | --- |
| 2000 | 5.8 [5.4, 6.3] | 1.8 [1.6, 2.1] | 6.1 [5.7, 6.5] | 1.4 [1.2, 1.6] | 0.4 [0.3, 0.5] | 0.7 [0.5, 0.8] | 0.1 [0.0, 0.1] | 5.4 [5.0, 5.8] | 0.8 [0.7, 1.0] |
| 2001 | 6.5 [6.1, 7.0] | 1.9 [1.7, 2.1] | 5.6 [5.2, 6.0] | 1.0 [0.9, 1.2] | 0.3 [0.3, 0.5] | 0.7 [0.6, 0.9] | 0.1 [0.0, 0.1] | 5.4 [5.1, 5.8] | 0.9 [0.7, 1.0] |
| 2002 | 6.3 [5.9, 6.8] | 2.0 [1.8, 2.3] | 6.1 [5.7, 6.6] | 1.2 [1.0, 1.4] | 0.4 [0.3, 0.6] | 0.6 [0.4, 0.7] | 0.0 [0.0, 0.1] | 5.1 [4.8, 5.5] | 1.0 [0.8, 1.2] |
| 2003 | 6.9 [6.5, 7.4] | 1.7 [1.5, 1.9] | 5.6 [5.2, 6.0] | 1.3 [1.1, 1.5] | 0.4 [0.3, 0.5] | 0.5 [0.4, 0.6] | 0.0 [0.0, 0.1] | 5.5 [5.1, 5.9] | 0.8 [0.7, 0.9] |
| 2004 | 8.0 [7.6, 8.5] | 1.7 [1.5, 1.9] | 5.3 [5.0, 5.7] | 1.1 [1.0, 1.3] | 0.3 [0.3, 0.4] | 0.6 [0.5, 0.8] | 0.1 [0.0, 0.2] | 5.5 [5.2, 5.9] | 1.0 [0.8, 1.2] |
| 2005 | 7.6 [7.1, 8.0] | 2.0 [1.7, 2.2] | 5.9 [5.5, 6.3] | 1.2 [1.0, 1.4] | 0.4 [0.3, 0.5] | 0.5 [0.4, 0.6] | 0.0 [0.0, 0.1] | 5.5 [5.1, 5.9] | 1.0 [0.9, 1.2] |
| 2006 | 8.8 [8.4, 9.3] | 2.1 [1.9, 2.3] | 5.8 [5.5, 6.2] | 1.2 [1.1, 1.4] | 0.3 [0.3, 0.4] | 0.4 [0.3, 0.5] | 0.1 [0.0, 0.1] | 5.1 [4.8, 5.5] | 0.9 [0.7, 1.0] |
| 2007 | 9.0 [8.5, 9.5] | 2.6 [2.3, 2.9] | 5.1 [4.8, 5.5] | 1.3 [1.1, 1.5] | 0.4 [0.3, 0.5] | 0.4 [0.4, 0.6] | 0.1 [0.0, 0.1] | 5.4 [5.0, 5.7] | 0.9 [0.7, 1.0] |
| 2008 | 10.0 [9.5, 10.5] | 2.9 [2.7, 3.2] | 4.7 [4.3, 5.0] | 1.2 [1.0, 1.4] | 0.3 [0.3, 0.4] | 0.7 [0.6, 0.8] | 0.1 [0.0, 0.1] | 5.5 [5.1, 5.8] | 0.7 [0.6, 0.9] |
| 2009 | 11.2 [10.7, 11.7] | 2.8 [2.5, 3.0] | 4.1 [3.8, 4.4] | 1.3 [1.1, 1.5] | 0.3 [0.2, 0.4] | 0.5 [0.4, 0.7] | 0.1 [0.1, 0.2] | 5.3 [4.9, 5.6] | 0.9 [0.7, 1.0] |
| 2010 | 12.3 [11.8, 12.9] | 3.0 [2.7, 3.2] | 4.3 [4.0, 4.7] | 1.1 [1.0, 1.3] | 0.4 [0.3, 0.5] | 0.5 [0.4, 0.6] | 0.1 [0.0, 0.1] | 5.6 [5.3, 6.0] | 0.8 [0.7, 0.9] |
| 2011 | 12.5 [12.0, 13.0] | 3.2 [2.9, 3.4] | 3.8 [3.5, 4.1] | 1.2 [1.0, 1.3] | 0.4 [0.3, 0.5] | 0.5 [0.4, 0.6] | 0.1 [0.0, 0.1] | 5.6 [5.3, 6.0] | 0.9 [0.8, 1.1] |
| 2012 | 13.1 [12.6, 13.7] | 3.4 [3.1, 3.6] | 4.1 [3.8, 4.4] | 1.1 [1.0, 1.3] | 0.4 [0.3, 0.5] | 0.5 [0.4, 0.6] | 0.1 [0.0, 0.1] | 5.4 [5.1, 5.8] | 0.8 [0.6, 0.9] |
| 2013 | 13.0 [12.5, 13.6] | 3.2 [3.0, 3.5] | 4.0 [3.7, 4.3] | 1.0 [0.9, 1.2] | 0.4 [0.3, 0.5] | 0.6 [0.5, 0.7] | 0.1 [0.1, 0.2] | 5.6 [5.3, 6.0] | 0.8 [0.6, 0.9] |
| 2014 | 13.2 [12.7, 13.7] | 3.6 [3.3, 3.9] | 3.4 [3.1, 3.7] | 1.0 [0.8, 1.1] | 0.3 [0.3, 0.4] | 0.6 [0.5, 0.7] | 0.1 [0.1, 0.2] | 6.1 [5.8, 6.5] | 0.7 [0.6, 0.8] |
| 2015 | 12.7 [12.2, 13.2] | 4.0 [3.7, 4.3] | 3.4 [3.1, 3.7] | 1.0 [0.8, 1.1] | 0.4 [0.3, 0.5] | 0.5 [0.4, 0.6] | 0.1 [0.0, 0.1] | 6.4 [6.0, 6.8] | 0.8 [0.7, 1.0] |
| 2016 | 12.5 [12.1, 13.0] | 5.5 [5.1, 5.8] | 3.7 [3.4, 4.0] | 0.9 [0.8, 1.1] | 0.4 [0.3, 0.5] | 0.4 [0.3, 0.5] | 0.0 [0.0, 0.1] | 6.4 [6.0, 6.8] | 0.6 [0.5, 0.7] |
| 2017 | 12.8 [12.3, 13.3] | 6.6 [6.3, 7.0] | 3.4 [3.1, 3.7] | 1.0 [0.8, 1.1] | 0.4 [0.3, 0.5] | 0.4 [0.3, 0.5] | 0.1 [0.1, 0.1] | 6.1 [5.8, 6.5] | 0.8 [0.7, 1.0] |
| 2018 | 14.4 [13.9, 14.9] | 7.2 [6.8, 7.5] | 3.4 [3.1, 3.6] | 1.0 [0.9, 1.2] | 0.4 [0.4, 0.5] | 0.4 [0.3, 0.5] | 0.1 [0.0, 0.1] | 6.1 [5.7, 6.5] | 0.7 [0.6, 0.8] |
| 2019 | 15.1 [14.6, 15.6] | 6.7 [6.3, 7.1] | 3.3 [3.0, 3.5] | 1.0 [0.9, 1.2] | 0.4 [0.3, 0.5] | 0.4 [0.3, 0.5] | 0.0 [0.0, 0.1] | 5.9 [5.5, 6.2] | 0.7 [0.5, 0.8] |
| 2020 | 16.1 [15.5, 16.6] | 8.6 [8.2, 9.0] | 2.7 [2.5, 3.0] | 1.0 [0.9, 1.2] | 0.5 [0.4, 0.6] | 0.4 [0.3, 0.4] | 0.1 [0.0, 0.1] | 6.1 [5.7, 6.4] | 0.7 [0.6, 0.8] |
| 2021 | 16.4 [15.9, 16.9] | 10.5 [10.0, 10.9] | 2.7 [2.5, 2.9] | 1.0 [0.9, 1.2] | 0.4 [0.3, 0.5] | 0.4 [0.3, 0.5] | 0.1 [0.0, 0.1] | 5.8 [5.4, 6.1] | 0.6 [0.5, 0.7] |
| 2022 | 18.8 [18.3, 19.4] | 9.7 [9.3, 10.2] | 2.9 [2.6, 3.1] | 1.2 [1.1, 1.4] | 0.5 [0.4, 0.6] | 0.4 [0.3, 0.5] | 0.0 [0.0, 0.1] | 6.7 [6.4, 7.1] | 0.6 [0.5, 0.7] |
| 2023 | 17.9 [17.4, 18.4] | 10.2 [9.7, 10.6] | 2.6 [2.4, 2.8] | 1.1 [1.0, 1.3] | 0.4 [0.3, 0.5] | 0.3 [0.3, 0.4] | 0.1 [0.0, 0.1] | 6.0 [5.7, 6.4] | 0.4 [0.4, 0.5] |

Table A3-6: Age-standardized mortality rates (per 100 000 population) from all causes, all unintentional causes, other unintentional causes and undetermined causes, females, Canada, from 2000 to 2023 (with 95% confidence intervals)

| Year | All causes | All unintentional causes | Other unintentional causes | Undetermined causes |
| --- | --- | --- | --- | --- |
| 2000 | 30.9 [30.0, 31.8] | 24.6 [23.7, 25.5] | 8.3 [7.8, 8.8] | 0.7 [0.6, 0.9] |
| 2001 | 30.0 [29.1, 31.0] | 23.4 [22.6, 24.2] | 7.2 [6.7, 7.7] | 0.9 [0.8, 1.1] |
| 2002 | 31.7 [30.8, 32.6] | 25.2 [24.4, 26.1] | 8.5 [8.0, 9.0] | 1.0 [0.8, 1.2] |
| 2003 | 32.0 [31.1, 32.9] | 25.0 [24.2, 25.8] | 8.6 [8.1, 9.1] | 1.4 [1.2, 1.6] |
| 2004 | 31.5 [30.6, 32.4] | 24.2 [23.4, 25.0] | 6.9 [6.5, 7.4] | 1.4 [1.3, 1.6] |
| 2005 | 31.8 [30.9, 32.7] | 24.4 [23.6, 25.2] | 6.9 [6.4, 7.3] | 1.4 [1.2, 1.6] |
| 2006 | 32.1 [31.2, 33.0] | 25.0 [24.3, 25.8] | 6.2 [5.8, 6.6] | 1.4 [1.3, 1.6] |
| 2007 | 32.0 [31.1, 32.8] | 24.7 [23.9, 25.5] | 5.8 [5.4, 6.2] | 1.3 [1.1, 1.5] |
| 2008 | 32.3 [31.5, 33.2] | 25.1 [24.3, 25.9] | 5.2 [4.9, 5.6] | 1.4 [1.2, 1.5] |
| 2009 | 32.1 [31.3, 33.0] | 24.7 [24.0, 25.5] | 4.4 [4.1, 4.8] | 1.5 [1.3, 1.7] |
| 2010 | 34.2 [33.3, 35.1] | 26.0 [25.2, 26.8] | 4.3 [4.0, 4.6] | 1.8 [1.6, 2.0] |
| 2011 | 33.5 [32.7, 34.4] | 25.5 [24.7, 26.2] | 4.0 [3.7, 4.3] | 1.4 [1.2, 1.6] |
| 2012 | 34.5 [33.6, 35.3] | 26.4 [25.6, 27.1] | 3.7 [3.4, 4.0] | 1.7 [1.5, 1.9] |
| 2013 | 34.5 [33.7, 35.4] | 26.2 [25.5, 26.9] | 3.8 [3.5, 4.0] | 1.6 [1.4, 1.8] |
| 2014 | 34.9 [34.1, 35.8] | 26.1 [25.4, 26.9] | 3.9 [3.6, 4.2] | 1.5 [1.3, 1.7] |
| 2015 | 34.9 [34.1, 35.8] | 25.9 [25.2, 26.6] | 3.8 [3.5, 4.1] | 1.2 [1.0, 1.3] |
| 2016 | 35.8 [34.9, 36.6] | 27.5 [26.7, 28.2] | 4.0 [3.7, 4.3] | 0.6 [0.5, 0.7] |
| 2017 | 37.3 [36.4, 38.2] | 28.7 [28.0, 29.5] | 4.0 [3.7, 4.3] | 0.7 [0.6, 0.8] |
| 2018 | 39.4 [38.5, 40.2] | 31.1 [30.3, 31.8] | 4.3 [4.0, 4.5] | 0.5 [0.4, 0.6] |
| 2019 | 39.4 [38.5, 40.3] | 31.1 [30.4, 31.9] | 4.2 [4.0, 4.5] | 0.6 [0.5, 0.7] |
| 2020 | 41.8 [40.9, 42.7] | 33.2 [32.4, 34.0] | 3.9 [3.6, 4.2] | 0.7 [0.6, 0.9] |
| 2021 | 44.8 [43.9, 45.7] | 36.7 [35.9, 37.5] | 5.3 [5.0, 5.6] | 0.6 [0.5, 0.7] |
| 2022 | 47.1 [46.2, 48.0] | 37.8 [37.0, 38.6] | 4.2 [4.0, 4.5] | 0.7 [0.6, 0.9] |
| 2023 | 45.0 [44.1, 45.9] | 36.9 [36.1, 37.7] | 4.3 [4.0, 4.6] | 0.6 [0.5, 0.7] |

Table A4-1: Number of deaths due to leading causes, aged 0 to 9 years, Canada, from 2000 to 2023

| Year | Falls | Poisonings | Land transport incidents | Suffocation | Drowning | Smoke/fire/flames | Struck by/against | Suicide | Homicide |
| --- | --- | --- | --- | --- | --- | --- | --- | --- | --- |
| 2000 | 6 | 1 | 91 | 36 | 35 | 21 | 1 | 0 | 31 |
| 2001 | 3 | 2 | 105 | 33 | 31 | 31 | 5 | 0 | 30 |
| 2002 | 8 | 5 | 86 | 38 | 39 | 15 | 4 | 0 | 34 |
| 2003 | 4 | 5 | 71 | 35 | 29 | 17 | 2 | 0 | 21 |
| 2004 | 13 | 2 | 58 | 25 | 36 | 17 | 2 | 0 | 26 |
| 2005 | 9 | 3 | 64 | 23 | 38 | 12 | 8 | 1 | 28 |
| 2006 | 5 | 3 | 58 | 37 | 35 | 12 | 3 | 0 | 28 |
| 2007 | 7 | 5 | 71 | 36 | 24 | 10 | 2 | 0 | 20 |
| 2008 | 5 | 1 | 52 | 34 | 32 | 27 | 0 | 0 | 24 |
| 2009 | 5 | 2 | 41 | 27 | 24 | 12 | 4 | 0 | 32 |
| 2010 | 4 | 4 | 52 | 38 | 35 | 10 | 3 | 0 | 29 |
| 2011 | 4 | 0 | 42 | 37 | 28 | 15 | 6 | 2 | 30 |
| 2012 | 2 | 0 | 58 | 38 | 34 | 10 | 7 | 0 | 22 |
| 2013 | 3 | 2 | 54 | 32 | 31 | 10 | 7 | 0 | 18 |
| 2014 | 4 | 1 | 37 | 35 | 21 | 8 | 2 | 0 | 23 |
| 2015 | 5 | 5 | 49 | 35 | 19 | 18 | 7 | 0 | 20 |
| 2016 | 4 | 5 | 43 | 31 | 26 | 15 | 3 | 1 | 7 |
| 2017 | 6 | 3 | 58 | 39 | 27 | 11 | 5 | 0 | 13 |
| 2018 | 2 | 4 | 41 | 32 | 22 | 11 | 4 | 2 | 9 |
| 2019 | 3 | 2 | 34 | 40 | 27 | 14 | 3 | 1 | 18 |
| 2020 | 4 | 4 | 39 | 28 | 40 | 4 | 5 | suppressed | 23 |
| 2021 | 3 | 3 | 35 | 18 | 20 | 14 | 0 | suppressed | 11 |
| 2022 | 4 | 3 | 32 | 20 | 33 | 21 | 3 | suppressed | 10 |
| 2023 | 2 | 9 | 29 | 21 | 36 | 6 | 4 | suppressed | 7 |

Table A4-2: Number of deaths from all causes, all unintentional causes, other unintentional causes and undetermined causes, aged 0 to 9 years, Canada, from 2000 to 2023

| Year | All causes | All unintentional causes | Other unintentional causes | Undetermined causes |
| --- | --- | --- | --- | --- |
| 2000 | 242 | 201 | 10 | 10 |
| 2001 | 257 | 217 | 7 | 10 |
| 2002 | 250 | 207 | 12 | 9 |
| 2003 | 203 | 180 | 17 | 2 |
| 2004 | 198 | 161 | 8 | 11 |
| 2005 | 201 | 169 | 12 | 3 |
| 2006 | 195 | 163 | 10 | 4 |
| 2007 | 192 | 170 | 15 | 2 |
| 2008 | 188 | 158 | 7 | 6 |
| 2009 | 158 | 125 | 10 | 1 |
| 2010 | 192 | 155 | 9 | 8 |
| 2011 | 184 | 141 | 9 | 11 |
| 2012 | 181 | 153 | 4 | 6 |
| 2013 | 180 | 152 | 13 | 10 |
| 2014 | 140 | 113 | 5 | 4 |
| 2015 | 177 | 146 | 8 | 11 |
| 2016 | 151 | 138 | 11 | 5 |
| 2017 | 179 | 152 | 3 | 14 |
| 2018 | 145 | 127 | 11 | 7 |
| 2019 | 165 | 135 | 12 | 11 |
| 2020 | 167 | 133 | 9 | 10 |
| 2021 | 128 | 104 | 11 | 12 |
| 2022 |  | 123 | 7 | 5 |
| 2023 | 131 | 117 | 10 | 6 |

Table A4-3: Crude mortality rates (per 100 000 population) due to leading causes, aged 0 to 9 years, Canada, from 2000 to 2023 (with 95% confidence intervals)

| Year | Falls | Poisonings | Land transport incidents | Suffocation | Drowning | Smoke/fire/ flames | Struck by/against | Suicide | Homicide |
| --- | --- | --- | --- | --- | --- | --- | --- | --- | --- |
| 2000 | 0.2 [0.0, 0.3] | 0.0 [0.0, 0.1] | 2.4 [1.9, 2.9] | 0.9 [0.6, 1.2] | 0.9 [0.6, 1.2] | 0.5 [0.3, 0.8] | 0.0 [0.0, 0.1] | 0.0 [0.0, 0.1] | 0.8 [0.5, 1.1] |
| 2001 | 0.1 [0.0, 0.2] | 0.1 [0.0, 0.2] | 2.8 [2.3, 3.3] | 0.9 [0.6, 1.2] | 0.8 [0.5, 1.1] | 0.8 [0.5, 1.1] | 0.1 [0.0, 0.2] | 0.0 [0.0, 0.1] | 0.8 [0.5, 1.1] |
| 2002 | 0.2 [0.1, 0.4] | 0.1 [0.0, 0.3] | 2.3 [1.8, 2.8] | 1.0 [0.7, 1.3] | 1.1 [0.7, 1.4] | 0.4 [0.2, 0.6] | 0.1 [0.0, 0.3] | 0.0 [0.0, 0.1] | 0.9 [0.6, 1.2] |
| 2003 | 0.1 [0.0, 0.3] | 0.1 [0.0, 0.3] | 1.9 [1.5, 2.4] | 1.0 [0.6, 1.3] | 0.8 [0.5, 1.1] | 0.5 [0.2, 0.7] | 0.1 [0.0, 0.2] | 0.0 [0.0, 0.1] | 0.6 [0.3, 0.8] |
| 2004 | 0.4 [0.2, 0.6] | 0.1 [0.0, 0.2] | 1.6 [1.2, 2.0] | 0.7 [0.4, 1.0] | 1.0 [0.7, 1.3] | 0.5 [0.2, 0.7] | 0.1 [0.0, 0.2] | 0.0 [0.0, 0.1] | 0.7 [0.4, 1.0] |
| 2005 | 0.3 [0.1, 0.4] | 0.1 [0.0, 0.2] | 1.8 [1.4, 2.2] | 0.6 [0.4, 0.9] | 1.1 [0.7, 1.4] | 0.3 [0.1, 0.5] | 0.2 [0.1, 0.4] | 0.0 [0.0, 0.2] | 0.8 [0.5, 1.1] |
| 2006 | 0.1 [0.0, 0.3] | 0.1 [0.0, 0.2] | 1.6 [1.2, 2.1] | 1.0 [0.7, 1.4] | 1.0 [0.7, 1.3] | 0.3 [0.1, 0.5] | 0.1 [0.0, 0.2] | 0.0 [0.0, 0.1] | 0.8 [0.5, 1.1] |
| 2007 | 0.2 [0.1, 0.3] | 0.1 [0.0, 0.3] | 2.0 [1.5, 2.5] | 1.0 [0.7, 1.3] | 0.7 [0.4, 0.9] | 0.3 [0.1, 0.5] | 0.1 [0.0, 0.2] | 0.0 [0.0, 0.1] | 0.6 [0.3, 0.8] |
| 2008 | 0.1 [0.0, 0.3] | 0.0 [0.0, 0.2] | 1.4 [1.1, 1.8] | 0.9 [0.6, 1.3] | 0.9 [0.6, 1.2] | 0.8 [0.5, 1.0] | 0.0 [0.0, 0.1] | 0.0 [0.0, 0.1] | 0.7 [0.4, 0.9] |
| 2009 | 0.1 [0.0, 0.3] | 0.1 [0.0, 0.2] | 1.1 [0.8, 1.5] | 0.7 [0.5, 1.0] | 0.7 [0.4, 0.9] | 0.3 [0.1, 0.5] | 0.1 [0.0, 0.3] | 0.0 [0.0, 0.1] | 0.9 [0.6, 1.2] |
| 2010 | 0.1 [0.0, 0.3] | 0.1 [0.0, 0.3] | 1.4 [1.0, 1.8] | 1.0 [0.7, 1.4] | 1.0 [0.6, 1.3] | 0.3 [0.1, 0.4] | 0.1 [0.0, 0.2] | 0.0 [0.0, 0.1] | 0.8 [0.5, 1.1] |
| 2011 | 0.1 [0.0, 0.3] | 0.0 [0.0, 0.1] | 1.1 [0.8, 1.5] | 1.0 [0.7, 1.3] | 0.8 [0.5, 1.0] | 0.4 [0.2, 0.6] | 0.2 [0.0, 0.3] | 0.1 [0.0, 0.2] | 0.8 [0.5, 1.1] |
| 2012 | 0.1 [0.0, 0.2] | 0.0 [0.0, 0.1] | 1.5 [1.1, 1.9] | 1.0 [0.7, 1.3] | 0.9 [0.6, 1.2] | 0.3 [0.1, 0.4] | 0.2 [0.0, 0.3] | 0.0 [0.0, 0.1] | 0.6 [0.3, 0.8] |
| 2013 | 0.1 [0.0, 0.2] | 0.1 [0.0, 0.2] | 1.4 [1.0, 1.8] | 0.8 [0.5, 1.1] | 0.8 [0.5, 1.1] | 0.3 [0.1, 0.4] | 0.2 [0.0, 0.3] | 0.0 [0.0, 0.1] | 0.5 [0.3, 0.7] |
| 2014 | 0.1 [0.0, 0.3] | 0.0 [0.0, 0.1] | 1.0 [0.6, 1.3] | 0.9 [0.6, 1.2] | 0.5 [0.3, 0.8] | 0.2 [0.1, 0.4] | 0.1 [0.0, 0.2] | 0.0 [0.0, 0.1] | 0.6 [0.4, 0.8] |
| 2015 | 0.1 [0.0, 0.2] | 0.1 [0.0, 0.2] | 1.3 [0.9, 1.6] | 0.9 [0.6, 1.2] | 0.5 [0.3, 0.7] | 0.5 [0.2, 0.7] | 0.2 [0.0, 0.3] | 0.0 [0.0, 0.1] | 0.5 [0.3, 0.7] |
| 2016 | 0.1 [0.0, 0.3] | 0.1 [0.0, 0.2] | 1.1 [0.8, 1.4] | 0.8 [0.5, 1.1] | 0.7 [0.4, 0.9] | 0.4 [0.2, 0.6] | 0.1 [0.0, 0.2] | 0.0 [0.0, 0.1] | 0.2 [0.0, 0.3] |
| 2017 | 0.2 [0.0, 0.3] | 0.1 [0.0, 0.2] | 1.5 [1.1, 1.8] | 1.0 [0.7, 1.3] | 0.7 [0.4, 0.9] | 0.3 [0.1, 0.4] | 0.1 [0.0, 0.2] | 0.0 [0.0, 0.1] | 0.3 [0.1, 0.5] |
| 2018 | 0.1 [0.0, 0.2] | 0.1 [0.0, 0.3] | 1.0 [0.7, 1.3] | 0.8 [0.5, 1.1] | 0.6 [0.3, 0.8] | 0.3 [0.1, 0.4] | 0.1 [0.0, 0.3] | 0.1 [0.0, 0.2] | 0.2 [0.1, 0.4] |
| 2019 | 0.1 [0.0, 0.2] | 0.0 [0.0, 0.2] | 0.8 [0.6, 1.1] | 1.0 [0.7, 1.3] | 0.7 [0.4, 0.9] | 0.3 [0.2, 0.5] | 0.1 [0.0, 0.2] | 0.0 [0.0, 0.1] | 0.4 [0.2, 0.7] |
| 2020 | 0.1 [0.0, 0.3] | 0.1 [0.0, 0.3] | 1.0 [0.7, 1.3] | 0.7 [0.4, 1.0] | 1.0 [0.7, 1.3] | 0.1 [0.0, 0.3] | 0.1 [0.0, 0.2] | Suppressed | 0.6 [0.3, 0.8] |
| 2021 | 0.1 [0.0, 0.2] | 0.1 [0.0, 0.2] | 0.9 [0.6, 1.2] | 0.5 [0.2, 0.7] | 0.5 [0.3, 0.7] | 0.4 [0.2, 0.5] | 0.0 [0.0, 0.1] | Suppressed | 0.3 [0.1, 0.4] |
| 2022 | 0.1 [0.0, 0.3] | 0.1 [0.0, 0.2] | 0.8 [0.5, 1.1] | 0.5 [0.3, 0.7] | 0.8 [0.5, 1.1] | 0.5 [0.3, 0.8] | 0.1 [0.0, 0.2] | Suppressed | 0.3 [0.1, 0.4] |
| 2023 | 0.1 [0.0, 0.2] | 0.2 [0.1, 0.4] | 0.7 [0.5, 1.0] | 0.5 [0.3, 0.8] | 0.9 [0.6, 1.2] | 0.2 [0.0, 0.3] | 0.1 [0.0, 0.3] | Suppressed | 0.2 [0.0, 0.3] |

Table A4-4: Crude mortality rates (per 100 000 population) from all causes, all unintentional causes, other unintentional causes and undetermined causes, aged 0 to 9 years, Canada, from 2000 to 2023 (with 95% confidence intervals)

| Year | All causes | All unintentional causes | Other unintentional causes | Undetermined causes |
| --- | --- | --- | --- | --- |
| 2000 | 6.3 [5.5, 7.1] | 5.3 [4.5, 6.0] | 0.3 [0.1, 0.4] | 0.3 [0.1, 0.4] |
| 2001 | 6.8 [6.0, 7.6] | 5.8 [5.0, 6.5] | 0.2 [0.0, 0.3] | 0.3 [0.1, 0.4] |
| 2002 | 6.7 [5.9, 7.6] | 5.6 [4.8, 6.3] | 0.3 [0.1, 0.5] | 0.2 [0.1, 0.4] |
| 2003 | 5.6 [4.8, 6.3] | 4.9 [4.2, 5.6] | 0.5 [0.2, 0.7] | 0.1 [0.0, 0.2] |
| 2004 | 5.5 [4.7, 6.2] | 4.5 [3.8, 5.1] | 0.2 [0.1, 0.4] | 0.3 [0.1, 0.5] |
| 2005 | 5.6 [4.8, 6.4] | 4.7 [4.0, 5.4] | 0.3 [0.1, 0.5] | 0.1 [0.0, 0.2] |
| 2006 | 5.5 [4.7, 6.3] | 4.6 [3.9, 5.3] | 0.3 [0.1, 0.5] | 0.1 [0.0, 0.3] |
| 2007 | 5.4 [4.6, 6.2] | 4.8 [4.1, 5.5] | 0.4 [0.2, 0.6] | 0.1 [0.0, 0.2] |
| 2008 | 5.2 [4.5, 6.0] | 4.4 [3.7, 5.1] | 0.2 [0.1, 0.3] | 0.2 [0.0, 0.3] |
| 2009 | 4.4 [3.7, 5.0] | 3.4 [2.8, 4.0] | 0.3 [0.1, 0.4] | 0.0 [0.0, 0.2] |
| 2010 | 5.2 [4.5, 6.0] | 4.2 [3.6, 4.9] | 0.2 [0.1, 0.4] | 0.2 [0.1, 0.4] |
| 2011 | 5.0 [4.2, 5.7] | 3.8 [3.2, 4.4] | 0.2 [0.1, 0.4] | 0.3 [0.1, 0.5] |
| 2012 | 4.8 [4.1, 5.5] | 4.1 [3.4, 4.7] | 0.1 [0.0, 0.3] | 0.2 [0.0, 0.3] |
| 2013 | 4.7 [4.0, 5.4] | 4.0 [3.3, 4.6] | 0.3 [0.2, 0.5] | 0.3 [0.1, 0.4] |
| 2014 | 3.6 [3.0, 4.2] | 2.9 [2.4, 3.5] | 0.1 [0.0, 0.2] | 0.1 [0.0, 0.3] |
| 2015 | 4.5 [3.9, 5.2] | 3.7 [3.1, 4.4] | 0.2 [0.1, 0.3] | 0.3 [0.1, 0.4] |
| 2016 | 3.8 [3.2, 4.4] | 3.5 [2.9, 4.1] | 0.3 [0.1, 0.4] | 0.1 [0.0, 0.2] |
| 2017 | 4.5 [3.8, 5.2] | 3.8 [3.2, 4.4] | 0.1 [0.0, 0.2] | 0.4 [0.2, 0.5] |
| 2018 | 3.6 [3.0, 4.2] | 3.2 [2.6, 3.7] | 0.3 [0.1, 0.4] | 0.2 [0.0, 0.3] |
| 2019 | 4.1 [3.5, 4.7] | 3.4 [2.8, 3.9] | 0.3 [0.1, 0.5] | 0.3 [0.1, 0.4] |
| 2020 | 4.2 [3.5, 4.8] | 3.3 [2.8, 3.9] | 0.2 [0.1, 0.4] | 0.2 [0.1, 0.4] |
| 2021 | 3.2 [2.7, 3.8] | 2.6 [2.1, 3.1] | 0.3 [0.1, 0.4] | 0.3 [0.1, 0.5] |
| 2022 | 0.0 [0.0, 0.0] | 3.1 [2.5, 3.6] | 0.2 [0.0, 0.3] | 0.1 [0.0, 0.2] |
| 2023 | 3.3 [2.7, 3.8] | 2.9 [2.4, 3.5] | 0.3 [0.1, 0.4] | 0.2 [0.0, 0.3] |

Table A5-1: Number of deaths due to leading causes, aged 10 to 19 years, Canada, from 2000 to 2023

| Year | Falls | Poisonings | Land transport incidents | Suffocation | Drowning | Smoke/fire/flames | Struck by/against | Suicide | Homicide |
| --- | --- | --- | --- | --- | --- | --- | --- | --- | --- |
| 2000 | 19 | 29 | 460 | 20 | 52 | 14 | 6 | 273 | 47 |
| 2001 | 11 | 25 | 424 | 19 | 46 | 13 | 8 | 237 | 52 |
| 2002 | 15 | 29 | 439 | 18 | 46 | 13 | 4 | 257 | 48 |
| 2003 | 14 | 20 | 423 | 10 | 43 | 8 | 3 | 244 | 47 |
| 2004 | 8 | 25 | 374 | 24 | 32 | 15 | 6 | 238 | 46 |
| 2005 | 12 | 23 | 424 | 13 | 42 | 11 | 5 | 256 | 61 |
| 2006 | 11 | 32 | 440 | 13 | 27 | 15 | 6 | 188 | 70 |
| 2007 | 18 | 29 | 375 | 7 | 43 | 6 | 3 | 219 | 74 |
| 2008 | 14 | 46 | 326 | 6 | 37 | 15 | 8 | 235 | 66 |
| 2009 | 12 | 36 | 293 | 3 | 28 | 11 | 5 | 229 | 74 |
| 2010 | 12 | 41 | 318 | 5 | 34 | 9 | 3 | 232 | 50 |
| 2011 | 6 | 36 | 262 | 9 | 30 | 4 | 5 | 237 | 53 |
| 2012 | 13 | 47 | 275 | 7 | 22 | 7 | 4 | 264 | 32 |
| 2013 | 11 | 35 | 216 | 9 | 28 | 11 | 2 | 203 | 41 |
| 2014 | 10 | 33 | 187 | 6 | 29 | 12 | 3 | 245 | 33 |
| 2015 | 11 | 50 | 176 | 11 | 20 | 16 | 1 | 240 | 29 |
| 2016 | 8 | 67 | 176 | 6 | 28 | 6 | 6 | 248 | 43 |
| 2017 | 7 | 105 | 155 | 7 | 31 | 5 | 4 | 269 | 53 |
| 2018 | 9 | 78 | 159 | 4 | 31 | 9 | 2 | 279 | 42 |
| 2019 | 8 | 79 | 145 | 7 | 17 | 5 | 3 | 259 | 46 |
| 2020 | 6 | 114 | 136 | 3 | 30 | 3 | 1 | 234 | 57 |
| 2021 | 6 | 95 | 143 | 5 | 18 | 12 | 1 | 259 | 43 |
| 2022 | 8 | 118 | 144 | 7 | 17 | 10 | 2 | 276 | 39 |
| 2023 | 4 | 102 | 143 | 5 | 26 | 2 | 3 | 239 | 29 |

Table A5-2: Number of deaths from all causes, all unintentional causes, other unintentional causes and undetermined causes, aged 10 to 19 years, Canada, from 2000 to 2023

| Year | All causes | All unintentional causes | Other unintentional causes | Undetermined causes |
| --- | --- | --- | --- | --- |
| 2000 | 969 | 634 | 34 | 15 |
| 2001 | 889 | 582 | 36 | 18 |
| 2002 | 912 | 591 | 27 | 16 |
| 2003 | 866 | 559 | 38 | 16 |
| 2004 | 812 | 507 | 23 | 21 |
| 2005 | 900 | 558 | 28 | 25 |
| 2006 | 839 | 563 | 19 | 18 |
| 2007 | 825 | 502 | 21 | 30 |
| 2008 | 798 | 480 | 28 | 17 |
| 2009 | 740 | 414 | 26 | 23 |
| 2010 | 758 | 451 | 29 | 25 |
| 2011 | 695 | 377 | 25 | 28 |
| 2012 | 713 | 399 | 24 | 18 |
| 2013 | 588 | 326 | 14 | 18 |
| 2014 | 604 | 301 | 21 | 25 |
| 2015 | 592 | 301 | 16 | 22 |
| 2016 | 608 | 306 | 9 | 11 |
| 2017 | 669 | 336 | 22 | 11 |
| 2018 | 636 | 310 | 18 | 5 |
| 2019 | 606 | 291 | 27 | 10 |
| 2020 | 616 | 309 | 16 | 16 |
| 2021 | 608 | 297 | 17 | 9 |
| 2022 | 654 | 324 | 18 | 15 |
| 2023 | 576 | 297 | 12 | 11 |

Table A5-3: Crude mortality rates (per 100 000 population) due to leading causes, aged 10 to 19 years, Canada, from 2000 to 2023 (with 95% confidence intervals)

| Year | Falls | Poisonings | Land transport incidents | Suffocation | Drowning | Smoke/fire/ flames | Struck by/against | Suicide | Homicide |
| --- | --- | --- | --- | --- | --- | --- | --- | --- | --- |
| 2000 | 0.5 [0.3, 0.7] | 0.7 [0.4, 1.0] | 11.1 [10.1, 12.1] | 0.5 [0.3, 0.7] | 1.3 [0.9, 1.6] | 0.3 [0.2, 0.5] | 0.1 [0.0, 0.3] | 6.6 [5.8, 7.4] | 1.1 [0.8, 1.5] |
| 2001 | 0.3 [0.1, 0.4] | 0.6 [0.4, 0.8] | 10.1 [9.1, 11.1] | 0.5 [0.2, 0.7] | 1.1 [0.8, 1.4] | 0.3 [0.1, 0.5] | 0.2 [0.1, 0.3] | 5.6 [4.9, 6.4] | 1.2 [0.9, 1.6] |
| 2002 | 0.4 [0.2, 0.5] | 0.7 [0.4, 0.9] | 10.3 [9.4, 11.3] | 0.4 [0.2, 0.6] | 1.1 [0.8, 1.4] | 0.3 [0.1, 0.5] | 0.1 [0.0, 0.2] | 6.1 [5.3, 6.8] | 1.1 [0.8, 1.5] |
| 2003 | 0.3 [0.2, 0.5] | 0.5 [0.3, 0.7] | 9.9 [9.0, 10.9] | 0.2 [0.1, 0.4] | 1.0 [0.7, 1.3] | 0.2 [0.1, 0.3] | 0.1 [0.0, 0.2] | 5.7 [5.0, 6.4] | 1.1 [0.8, 1.4] |
| 2004 | 0.2 [0.1, 0.3] | 0.6 [0.4, 0.8] | 8.7 [7.8, 9.6] | 0.6 [0.3, 0.8] | 0.7 [0.5, 1.0] | 0.4 [0.2, 0.5] | 0.1 [0.0, 0.3] | 5.6 [4.8, 6.3] | 1.1 [0.8, 1.4] |
| 2005 | 0.3 [0.1, 0.4] | 0.5 [0.3, 0.8] | 9.9 [8.9, 10.8] | 0.3 [0.1, 0.5] | 1.0 [0.7, 1.3] | 0.3 [0.1, 0.4] | 0.1 [0.0, 0.2] | 6.0 [5.2, 6.7] | 1.4 [1.1, 1.8] |
| 2006 | 0.3 [0.1, 0.4] | 0.7 [0.5, 1.0] | 10.2 [9.3, 11.2] | 0.3 [0.1, 0.5] | 0.6 [0.4, 0.9] | 0.3 [0.2, 0.5] | 0.1 [0.0, 0.3] | 4.4 [3.7, 5.0] | 1.6 [1.2, 2.0] |
| 2007 | 0.4 [0.2, 0.6] | 0.7 [0.4, 0.9] | 8.7 [7.8, 9.6] | 0.2 [0.0, 0.3] | 1.0 [0.7, 1.3] | 0.1 [0.0, 0.3] | 0.1 [0.0, 0.2] | 5.1 [4.4, 5.8] | 1.7 [1.3, 2.1] |
| 2008 | 0.3 [0.2, 0.5] | 1.1 [0.8, 1.4] | 7.6 [6.8, 8.4] | 0.1 [0.0, 0.3] | 0.9 [0.6, 1.1] | 0.4 [0.2, 0.5] | 0.2 [0.1, 0.3] | 5.5 [4.8, 6.2] | 1.5 [1.2, 1.9] |
| 2009 | 0.3 [0.1, 0.4] | 0.8 [0.6, 1.1] | 6.9 [6.1, 7.7] | 0.1 [0.0, 0.2] | 0.7 [0.4, 0.9] | 0.3 [0.1, 0.4] | 0.1 [0.0, 0.2] | 5.4 [4.7, 6.1] | 1.7 [1.3, 2.1] |
| 2010 | 0.3 [0.1, 0.4] | 1.0 [0.7, 1.3] | 7.6 [6.7, 8.4] | 0.1 [0.0, 0.2] | 0.8 [0.5, 1.1] | 0.2 [0.1, 0.4] | 0.1 [0.0, 0.2] | 5.5 [4.8, 6.2] | 1.2 [0.9, 1.5] |
| 2011 | 0.1 [0.0, 0.3] | 0.9 [0.6, 1.1] | 6.3 [5.5, 7.1] | 0.2 [0.1, 0.4] | 0.7 [0.5, 1.0] | 0.1 [0.0, 0.2] | 0.1 [0.0, 0.2] | 5.7 [5.0, 6.4] | 1.3 [0.9, 1.6] |
| 2012 | 0.3 [0.1, 0.5] | 1.1 [0.8, 1.5] | 6.7 [5.9, 7.5] | 0.2 [0.0, 0.3] | 0.5 [0.3, 0.8] | 0.2 [0.0, 0.3] | 0.1 [0.0, 0.3] | 6.4 [5.7, 7.2] | 0.8 [0.5, 1.1] |
| 2013 | 0.3 [0.1, 0.4] | 0.9 [0.6, 1.2] | 5.3 [4.6, 6.1] | 0.2 [0.1, 0.4] | 0.7 [0.4, 0.9] | 0.3 [0.1, 0.4] | 0.0 [0.0, 0.2] | 5.0 [4.3, 5.7] | 1.0 [0.7, 1.3] |
| 2014 | 0.2 [0.1, 0.4] | 0.8 [0.5, 1.1] | 4.7 [4.0, 5.3] | 0.1 [0.0, 0.3] | 0.7 [0.5, 1.0] | 0.3 [0.1, 0.5] | 0.1 [0.0, 0.2] | 6.1 [5.3, 6.9] | 0.8 [0.5, 1.1] |
| 2015 | 0.3 [0.1, 0.4] | 1.3 [0.9, 1.6] | 4.4 [3.8, 5.1] | 0.3 [0.1, 0.4] | 0.5 [0.3, 0.7] | 0.4 [0.2, 0.6] | 0.0 [0.0, 0.1] | 6.0 [5.2, 6.8] | 0.7 [0.5, 1.0] |
| 2016 | 0.2 [0.1, 0.3] | 1.7 [1.3, 2.1] | 4.4 [3.7, 5.0] | 0.1 [0.0, 0.3] | 0.7 [0.4, 1.0] | 0.1 [0.0, 0.3] | 0.1 [0.0, 0.3] | 6.2 [5.4, 7.0] | 1.1 [0.8, 1.4] |
| 2017 | 0.2 [0.0, 0.3] | 2.6 [2.1, 3.1] | 3.8 [3.2, 4.4] | 0.2 [0.0, 0.3] | 0.8 [0.5, 1.0] | 0.1 [0.0, 0.2] | 0.1 [0.0, 0.3] | 6.7 [5.9, 7.5] | 1.3 [1.0, 1.7] |
| 2018 | 0.2 [0.1, 0.4] | 1.9 [1.5, 2.3] | 3.9 [3.3, 4.5] | 0.1 [0.0, 0.3] | 0.8 [0.5, 1.0] | 0.2 [0.1, 0.4] | 0.0 [0.0, 0.2] | 6.8 [6.0, 7.6] | 1.0 [0.7, 1.3] |
| 2019 | 0.2 [0.1, 0.3] | 1.9 [1.5, 2.3] | 3.5 [2.9, 4.1] | 0.2 [0.0, 0.3] | 0.4 [0.2, 0.6] | 0.1 [0.0, 0.2] | 0.1 [0.0, 0.2] | 6.3 [5.5, 7.0] | 1.1 [0.8, 1.4] |
| 2020 | 0.1 [0.0, 0.3] | 2.7 [2.2, 3.2] | 3.3 [2.7, 3.8] | 0.1 [0.0, 0.2] | 0.7 [0.5, 1.0] | 0.1 [0.0, 0.2] | 0.0 [0.0, 0.1] | 5.6 [4.9, 6.3] | 1.4 [1.0, 1.7] |
| 2021 | 0.1 [0.0, 0.3] | 2.3 [1.8, 2.7] | 3.4 [2.9, 4.0] | 0.1 [0.0, 0.2] | 0.4 [0.2, 0.6] | 0.3 [0.1, 0.5] | 0.0 [0.0, 0.1] | 6.2 [5.4, 7.0] | 1.0 [0.7, 1.3] |
| 2022 | 0.2 [0.1, 0.3] | 2.8 [2.3, 3.2] | 3.4 [2.8, 3.9] | 0.2 [0.0, 0.3] | 0.4 [0.2, 0.6] | 0.2 [0.1, 0.4] | 0.0 [0.0, 0.2] | 6.4 [5.7, 7.2] | 0.9 [0.6, 1.2] |
| 2023 | 0.1 [0.0, 0.2] | 2.3 [1.9, 2.8] | 3.2 [2.7, 3.8] | 0.1 [0.0, 0.2] | 0.6 [0.4, 0.8] | 0.0 [0.0, 0.2] | 0.1 [0.0, 0.2] | 5.4 [4.7, 6.1] | 0.7 [0.4, 0.9] |

Table A5-4: Crude mortality rates (per 100 000 population) from all causes, all unintentional causes, other unintentional causes and undetermined causes, aged 10 to 19 years, Canada, from 2000 to 2023 (with 95% confidence intervals)

| Year | All causes | All unintentional causes | Other unintentional causes | Undetermined causes |
| --- | --- | --- | --- | --- |
| 2000 | 23.3 [21.9, 24.8] | 15.3 [14.1, 16.5] | 0.8 [0.5, 1.1] | 0.4 [0.2, 0.5] |
| 2001 | 21.2 [19.8, 22.6] | 13.9 [12.7, 15] | 0.9 [0.6, 1.1] | 0.4 [0.2, 0.6] |
| 2002 | 21.5 [20.1, 22.9] | 13.9 [12.8, 15.1] | 0.6 [0.4, 0.9] | 0.4 [0.2, 0.6] |
| 2003 | 20.3 [18.9, 21.6] | 13.1 [12.0, 14.2] | 0.9 [0.6, 1.2] | 0.4 [0.2, 0.6] |
| 2004 | 18.9 [17.6, 20.3] | 11.8 [10.8, 12.9] | 0.5 [0.3, 0.8] | 0.5 [0.3, 0.7] |
| 2005 | 20.9 [19.6, 22.3] | 13.0 [11.9, 14.1] | 0.7 [0.4, 0.9] | 0.6 [0.4, 0.8] |
| 2006 | 19.5 [18.2, 20.8] | 13.1 [12.0, 14.1] | 0.4 [0.2, 0.6] | 0.4 [0.2, 0.6] |
| 2007 | 19.2 [17.9, 20.5] | 11.7 [10.7, 12.7] | 0.5 [0.3, 0.7] | 0.7 [0.4, 0.9] |
| 2008 | 18.6 [17.3, 19.9] | 11.2 [10.2, 12.2] | 0.7 [0.4, 0.9] | 0.4 [0.2, 0.6] |
| 2009 | 17.4 [16.2, 18.7] | 9.7 [8.8, 10.7] | 0.6 [0.4, 0.8] | 0.5 [0.3, 0.8] |
| 2010 | 18.0 [16.7, 19.3] | 10.7 [9.7, 11.7] | 0.7 [0.4, 0.9] | 0.6 [0.4, 0.8] |
| 2011 | 16.7 [15.5, 18.0] | 9.1 [8.2, 10] | 0.6 [0.4, 0.8] | 0.7 [0.4, 0.9] |
| 2012 | 17.4 [16.1, 18.7] | 9.7 [8.8, 10.7] | 0.6 [0.4, 0.8] | 0.4 [0.2, 0.6] |
| 2013 | 14.5 [13.4, 15.7] | 8.1 [7.2, 8.9] | 0.3 [0.2, 0.5] | 0.4 [0.2, 0.7] |
| 2014 | 15.0 [13.8, 16.2] | 7.5 [6.6, 8.3] | 0.5 [0.3, 0.7] | 0.6 [0.4, 0.9] |
| 2015 | 14.8 [13.6, 16.0] | 7.5 [6.7, 8.4] | 0.4 [0.2, 0.6] | 0.6 [0.3, 0.8] |
| 2016 | 15.2 [14.0, 16.4] | 7.6 [6.8, 8.5] | 0.2 [0.1, 0.4] | 0.3 [0.1, 0.4] |
| 2017 | 16.6 [15.3, 17.8] | 8.3 [7.4, 9.2] | 0.5 [0.3, 0.8] | 0.3 [0.1, 0.4] |
| 2018 | 15.6 [14.4, 16.8] | 7.6 [6.7, 8.4] | 0.4 [0.2, 0.6] | 0.1 [0.0, 0.2] |
| 2019 | 14.6 [13.5, 15.8] | 7.0 [6.2, 7.8] | 0.7 [0.4, 0.9] | 0.2 [0.1, 0.4] |
| 2020 | 14.8 [13.6, 15.9] | 7.4 [6.6, 8.2] | 0.4 [0.2, 0.6] | 0.4 [0.2, 0.6] |
| 2021 | 14.6 [13.4, 15.7] | 7.1 [6.3, 7.9] | 0.4 [0.2, 0.6] | 0.2 [0.1, 0.4] |
| 2022 | 15.3 [14.1, 16.4] | 7.6 [6.7, 8.4] | 0.4 [0.2, 0.6] | 0.3 [0.2, 0.5] |
| 2023 | 13.0 [12.0, 14.1] | 6.7 [6.0, 7.5] | 0.3 [0.1, 0.4] | 0.2 [0.1, 0.4] |

Table A6-1: Number of deaths due to leading causes, aged 20 to 44 years, Canada, from 2000 to 2023

| Year | Falls | Poisonings | Land transport incidents | Suffocation | Drowning | Smoke/fire/flames | Struck by/against | Suicide | Homicide |
| --- | --- | --- | --- | --- | --- | --- | --- | --- | --- |
| 2000 | 87 | 558 | 1240 | 66 | 134 | 59 | 45 | 1861 | 281 |
| 2001 | 97 | 565 | 1229 | 71 | 139 | 68 | 39 | 1826 | 291 |
| 2002 | 120 | 574 | 1247 | 79 | 138 | 80 | 28 | 1748 | 268 |
| 2003 | 109 | 485 | 1284 | 53 | 147 | 52 | 29 | 1757 | 277 |
| 2004 | 89 | 482 | 1245 | 54 | 122 | 67 | 31 | 1670 | 312 |
| 2005 | 94 | 554 | 1317 | 41 | 124 | 42 | 36 | 1691 | 361 |
| 2006 | 86 | 560 | 1303 | 54 | 129 | 54 | 25 | 1504 | 319 |
| 2007 | 92 | 657 | 1277 | 58 | 115 | 70 | 36 | 1558 | 319 |
| 2008 | 82 | 607 | 1093 | 47 | 126 | 65 | 26 | 1539 | 333 |
| 2009 | 90 | 700 | 969 | 38 | 103 | 44 | 19 | 1612 | 326 |
| 2010 | 89 | 690 | 983 | 36 | 133 | 50 | 27 | 1571 | 284 |
| 2011 | 99 | 810 | 902 | 47 | 130 | 39 | 25 | 1576 | 301 |
| 2012 | 97 | 814 | 936 | 34 | 136 | 38 | 29 | 1526 | 282 |
| 2013 | 87 | 866 | 876 | 34 | 107 | 58 | 28 | 1512 | 250 |
| 2014 | 95 | 1009 | 791 | 38 | 102 | 36 | 28 | 1603 | 240 |
| 2015 | 90 | 1207 | 806 | 34 | 116 | 37 | 20 | 1694 | 270 |
| 2016 | 86 | 1850 | 853 | 53 | 106 | 36 | 13 | 1715 | 264 |
| 2017 | 90 | 2615 | 832 | 46 | 116 | 36 | 21 | 1759 | 305 |
| 2018 | 79 | 2694 | 822 | 36 | 116 | 32 | 23 | 1787 | 306 |
| 2019 | 99 | 2361 | 706 | 33 | 113 | 38 | 21 | 1869 | 289 |
| 2020 | 104 | 3645 | 737 | 42 | 168 | 31 | 27 | 1910 | 324 |
| 2021 | 80 | 4252 | 729 | 50 | 147 | 48 | 21 | 1921 | 296 |
| 2022 | 90 | 3922 | 782 | 36 | 117 | 37 | 13 | 2098 | 278 |
| 2023 | 81 | 3950 | 682 | 29 | 91 | 45 | 12 | 1990 | 206 |

Table A6-2: Number of deaths from all causes, all unintentional causes, other unintentional causes and undetermined causes, aged 20 to 44 years, Canada, from 2000 to 2023

| Year | All causes | All unintentional causes | Other unintentional causes | Undetermined causes |
| --- | --- | --- | --- | --- |
| 2000 | 4716 | 2390 | 201 | 184 |
| 2001 | 4765 | 2431 | 223 | 217 |
| 2002 | 4683 | 2476 | 210 | 191 |
| 2003 | 4729 | 2368 | 209 | 327 |
| 2004 | 4523 | 2261 | 171 | 280 |
| 2005 | 4790 | 2400 | 192 | 338 |
| 2006 | 4524 | 2408 | 197 | 293 |
| 2007 | 4668 | 2484 | 179 | 307 |
| 2008 | 4430 | 2241 | 195 | 317 |
| 2009 | 4334 | 2112 | 149 | 284 |
| 2010 | 4333 | 2172 | 164 | 306 |
| 2011 | 4348 | 2195 | 143 | 276 |
| 2012 | 4399 | 2247 | 163 | 344 |
| 2013 | 4304 | 2198 | 142 | 344 |
| 2014 | 4488 | 2233 | 134 | 412 |
| 2015 | 4737 | 2418 | 108 | 355 |
| 2016 | 5217 | 3125 | 128 | 113 |
| 2017 | 6064 | 3881 | 125 | 119 |
| 2018 | 6143 | 3944 | 142 | 106 |
| 2019 | 5834 | 3543 | 172 | 133 |
| 2020 | 7318 | 4917 | 163 | 167 |
| 2021 | 7891 | 5494 | 167 | 180 |
| 2022 | 7693 | 5160 | 163 | 157 |
| 2023 | 7402 | 5055 | 165 | 151 |

Table A6-3: Crude mortality rates (per 100 000 population) due to leading causes, aged 20 to 44 years, Canada, from 2000 to 2023 (with 95% confidence intervals)

| Year | Falls | Poisonings | Land transport incidents | Suffocation | Drowning | Smoke/fire/ flames | Struck by/against | Suicide | Homicide |
| --- | --- | --- | --- | --- | --- | --- | --- | --- | --- |
| 2000 | 0.7 [0.6, 0.9] | 4.8 [4.4, 5.2] | 10.6 [10, 11.2] | 0.6 [0.4, 0.7] | 1.1 [1.0, 1.3] | 0.5 [0.4, 0.6] | 0.4 [0.3, 0.5] | 15.9 [15.2, 16.6] | 2.4 [2.1, 2.7] |
| 2001 | 0.8 [0.7, 1.0] | 4.8 [4.4, 5.2] | 10.5 [9.9, 11.1] | 0.6 [0.5, 0.7] | 1.2 [1.0, 1.4] | 0.6 [0.4, 0.7] | 0.3 [0.2, 0.4] | 15.6 [14.9, 16.3] | 2.5 [2.2, 2.8] |
| 2002 | 1.0 [0.8, 1.2] | 4.9 [4.5, 5.3] | 10.6 [10.0, 11.2] | 0.7 [0.5, 0.8] | 1.2 [1.0, 1.4] | 0.7 [0.5, 0.8] | 0.2 [0.2, 0.3] | 14.9 [14.2, 15.6] | 2.3 [2.0, 2.6] |
| 2003 | 0.9 [0.8, 1.1] | 4.1 [3.8, 4.5] | 11.0 [10.4, 11.6] | 0.5 [0.3, 0.6] | 1.3 [1.1, 1.5] | 0.4 [0.3, 0.6] | 0.2 [0.2, 0.3] | 15.0 [14.3, 15.7] | 2.4 [2.1, 2.6] |
| 2004 | 0.8 [0.6, 0.9] | 4.1 [3.8, 4.5] | 10.6 [10.1, 11.2] | 0.5 [0.3, 0.6] | 1.0 [0.9, 1.2] | 0.6 [0.4, 0.7] | 0.3 [0.2, 0.4] | 14.3 [13.6, 15.0] | 2.7 [2.4, 3.0] |
| 2005 | 0.8 [0.6, 1.0] | 4.7 [4.4, 5.1] | 11.3 [10.7, 11.9] | 0.4 [0.2, 0.5] | 1.1 [0.9, 1.2] | 0.4 [0.3, 0.5] | 0.3 [0.2, 0.4] | 14.5 [13.8, 15.2] | 3.1 [2.8, 3.4] |
| 2006 | 0.7 [0.6, 0.9] | 4.8 [4.4, 5.2] | 11.2 [10.6, 11.8] | 0.5 [0.3, 0.6] | 1.1 [0.9, 1.3] | 0.5 [0.3, 0.6] | 0.2 [0.1, 0.3] | 12.9 [12.3, 13.6] | 2.7 [2.4, 3.0] |
| 2007 | 0.8 [0.6, 1.0] | 5.7 [5.2, 6.1] | 11.0 [10.4, 11.6] | 0.5 [0.4, 0.6] | 1.0 [0.8, 1.2] | 0.6 [0.5, 0.7] | 0.3 [0.2, 0.4] | 13.4 [12.7, 14.1] | 2.7 [2.4, 3.0] |
| 2008 | 0.7 [0.6, 0.9] | 5.2 [4.8, 5.7] | 9.4 [8.9, 10.0] | 0.4 [0.3, 0.5] | 1.1 [0.9, 1.3] | 0.6 [0.4, 0.7] | 0.2 [0.1, 0.3] | 13.3 [12.6, 13.9] | 2.9 [2.6, 3.2] |
| 2009 | 0.8 [0.6, 0.9] | 6.0 [5.6, 6.5] | 8.3 [7.8, 8.9] | 0.3 [0.2, 0.4] | 0.9 [0.7, 1.1] | 0.4 [0.3, 0.5] | 0.2 [0.1, 0.2] | 13.9 [13.2, 14.6] | 2.8 [2.5, 3.1] |
| 2010 | 0.8 [0.6, 0.9] | 5.9 [5.5, 6.4] | 8.4 [7.9, 9.0] | 0.3 [0.2, 0.4] | 1.1 [0.9, 1.3] | 0.4 [0.3, 0.5] | 0.2 [0.1, 0.3] | 13.5 [12.8, 14.2] | 2.4 [2.2, 2.7] |
| 2011 | 0.8 [0.7, 1.0] | 6.9 [6.4, 7.4] | 7.7 [7.2, 8.2] | 0.4 [0.3, 0.5] | 1.1 [0.9, 1.3] | 0.3 [0.2, 0.4] | 0.2 [0.1, 0.3] | 13.5 [12.8, 14.1] | 2.6 [2.3, 2.9] |
| 2012 | 0.8 [0.7, 1.0] | 6.9 [6.4, 7.4] | 7.9 [7.4, 8.4] | 0.3 [0.2, 0.4] | 1.2 [1.0, 1.3] | 0.3 [0.2, 0.4] | 0.2 [0.2, 0.3] | 12.9 [12.3, 13.6] | 2.4 [2.1, 2.7] |
| 2013 | 0.7 [0.6, 0.9] | 7.3 [6.8, 7.8] | 7.4 [6.9, 7.8] | 0.3 [0.2, 0.4] | 0.9 [0.7, 1.1] | 0.5 [0.4, 0.6] | 0.2 [0.1, 0.3] | 12.7 [12.1, 13.3] | 2.1 [1.8, 2.4] |
| 2014 | 0.8 [0.6, 1.0] | 8.4 [7.9, 8.9] | 6.6 [6.1, 7.1] | 0.3 [0.2, 0.4] | 0.9 [0.7, 1.0] | 0.3 [0.2, 0.4] | 0.2 [0.1, 0.3] | 13.4 [12.7, 14.0] | 2.0 [1.7, 2.3] |
| 2015 | 0.7 [0.6, 0.9] | 10.1 [9.5, 10.6] | 6.7 [6.3, 7.2] | 0.3 [0.2, 0.4] | 1.0 [0.8, 1.1] | 0.3 [0.2, 0.4] | 0.2 [0.1, 0.2] | 14.1 [13.4, 14.8] | 2.2 [2.0, 2.5] |
| 2016 | 0.7 [0.6, 0.9] | 15.3 [14.6, 16.0] | 7.1 [6.6, 7.5] | 0.4 [0.3, 0.6] | 0.9 [0.7, 1.0] | 0.3 [0.2, 0.4] | 0.1 [0.0, 0.2] | 14.2 [13.5, 14.9] | 2.2 [1.9, 2.4] |
| 2017 | 0.7 [0.6, 0.9] | 21.4 [20.5, 22.2] | 6.8 [6.3, 7.3] | 0.4 [0.3, 0.5] | 0.9 [0.8, 1.1] | 0.3 [0.2, 0.4] | 0.2 [0.1, 0.2] | 14.4 [13.7, 15.0] | 2.5 [2.2, 2.8] |
| 2018 | 0.6 [0.5, 0.8] | 21.6 [20.8, 22.4] | 6.6 [6.1, 7.0] | 0.3 [0.2, 0.4] | 0.9 [0.8, 1.1] | 0.3 [0.2, 0.3] | 0.2 [0.1, 0.3] | 14.3 [13.7, 15.0] | 2.5 [2.2, 2.7] |
| 2019 | 0.8 [0.6, 0.9] | 18.5 [17.8, 19.3] | 5.5 [5.1, 6.0] | 0.3 [0.2, 0.3] | 0.9 [0.7, 1.1] | 0.3 [0.2, 0.4] | 0.2 [0.1, 0.2] | 14.7 [14.0, 15.3] | 2.3 [2.0, 2.5] |
| 2020 | 0.8 [0.7, 1.0] | 28.3 [27.4, 29.2] | 5.7 [5.3, 6.1] | 0.3 [0.2, 0.4] | 1.3 [1.1, 1.5] | 0.2 [0.2, 0.3] | 0.2 [0.1, 0.3] | 14.8 [14.2, 15.5] | 2.5 [2.2, 2.8] |
| 2021 | 0.6 [0.5, 0.8] | 32.9 [31.9, 33.9] | 5.6 [5.2, 6.0] | 0.4 [0.3, 0.5] | 1.1 [1.0, 1.3] | 0.4 [0.3, 0.5] | 0.2 [0.1, 0.2] | 14.9 [14.2, 15.5] | 2.3 [2.0, 2.5] |
| 2022 | 0.7 [0.5, 0.8] | 29.5 [28.6, 30.4] | 5.9 [5.5, 6.3] | 0.3 [0.2, 0.4] | 0.9 [0.7, 1.0] | 0.3 [0.2, 0.4] | 0.1 [0.0, 0.2] | 15.8 [15.1, 16.5] | 2.1 [1.8, 2.3] |
| 2023 | 0.6 [0.5, 0.7] | 28.3 [27.4, 29.2] | 4.9 [4.5, 5.3] | 0.2 [0.1, 0.3] | 0.7 [0.5, 0.8] | 0.3 [0.2, 0.4] | 0.1 [0.0, 0.1] | 14.3 [13.6, 14.9] | 1.5 [1.3, 1.7] |

Table A6-4: Crude mortality rates (per 100 000 population) from all causes, all unintentional causes, other unintentional causes and undetermined causes, aged 20 to 44 years, Canada, from 2000 to 2023 (with 95% confidence intervals)

| Year | All causes | All unintentional causes | Other unintentional causes | Undetermined causes |
| --- | --- | --- | --- | --- |
| 2000 | 40.4 [39.2, 41.5] | 20.5 [19.6, 21.3] | 1.7 [1.5, 2.0] | 1.6 [1.3, 1.8] |
| 2001 | 40.7 [39.5, 41.8] | 20.7 [19.9, 21.6] | 1.9 [1.7, 2.2] | 1.9 [1.6, 2.1] |
| 2002 | 39.9 [38.8, 41.1] | 21.1 [20.3, 21.9] | 1.8 [1.5, 2.0] | 1.6 [1.4, 1.9] |
| 2003 | 40.4 [39.2, 41.5] | 20.2 [19.4, 21.0] | 1.8 [1.5, 2.0] | 2.8 [2.5, 3.1] |
| 2004 | 38.7 [37.6, 39.8] | 19.3 [18.5, 20.1] | 1.5 [1.2, 1.7] | 2.4 [2.1, 2.7] |
| 2005 | 41.0 [39.9, 42.2] | 20.6 [19.7, 21.4] | 1.6 [1.4, 1.9] | 2.9 [2.6, 3.2] |
| 2006 | 38.8 [37.7, 40.0] | 20.7 [19.9, 21.5] | 1.7 [1.5, 1.9] | 2.5 [2.2, 2.8] |
| 2007 | 40.2 [39.0, 41.3] | 21.4 [20.5, 22.2] | 1.5 [1.3, 1.8] | 2.6 [2.3, 2.9] |
| 2008 | 38.2 [37.1, 39.3] | 19.3 [18.5, 20.1] | 1.7 [1.4, 1.9] | 2.7 [2.4, 3.0] |
| 2009 | 37.3 [36.2, 38.5] | 18.2 [17.4, 19.0] | 1.3 [1.1, 1.5] | 2.4 [2.2, 2.7] |
| 2010 | 37.2 [36.1, 38.3] | 18.6 [17.9, 19.4] | 1.4 [1.2, 1.6] | 2.6 [2.3, 2.9] |
| 2011 | 37.1 [36.0, 38.2] | 18.8 [18.0, 19.5] | 1.2 [1.0, 1.4] | 2.4 [2.1, 2.6] |
| 2012 | 37.2 [36.1, 38.3] | 19.0 [18.2, 19.8] | 1.4 [1.2, 1.6] | 2.9 [2.6, 3.2] |
| 2013 | 36.1 [35.1, 37.2] | 18.5 [17.7, 19.2] | 1.2 [1.0, 1.4] | 2.9 [2.6, 3.2] |
| 2014 | 37.4 [36.3, 38.5] | 18.6 [17.9, 19.4] | 1.1 [0.9, 1.3] | 3.4 [3.1, 3.8] |
| 2015 | 39.5 [38.3, 40.6] | 20.1 [19.3, 20.9] | 0.9 [0.7, 1.1] | 3.0 [2.6, 3.3] |
| 2016 | 43.1 [42.0, 44.3] | 25.8 [24.9, 26.7] | 1.1 [0.9, 1.2] | 0.9 [0.8, 1.1] |
| 2017 | 49.5 [48.3, 50.8] | 31.7 [30.7, 32.7] | 1.0 [0.8, 1.2] | 1.0 [0.8, 1.1] |
| 2018 | 49.2 [48.0, 50.4] | 31.6 [30.6, 32.6] | 1.1 [1.0, 1.3] | 0.8 [0.7, 1.0] |
| 2019 | 45.8 [44.6, 47.0] | 27.8 [26.9, 28.7] | 1.4 [1.1, 1.6] | 1.0 [0.9, 1.2] |
| 2020 | 56.8 [55.5, 58.1] | 38.1 [37.1, 39.2] | 1.3 [1.1, 1.5] | 1.3 [1.1, 1.5] |
| 2021 | 61.0 [59.7, 62.4] | 42.5 [41.4, 43.6] | 1.3 [1.1, 1.5] | 1.4 [1.2, 1.6] |
| 2022 | 57.9 [56.6, 59.2] | 38.8 [37.8, 39.9] | 1.2 [1.0, 1.4] | 1.2 [1.0, 1.4] |
| 2023 | 53.0 [51.8, 54.2] | 36.2 [35.2, 37.2] | 1.2 [1.0, 1.4] | 1.1 [0.9, 1.3] |

Table A7-1: Number of deaths due to leading causes, aged 45 to 64 years, Canada, from 2000 to 2023

| Year | Falls | Poisonings | Land transport incidents | Suffocation | Drowning | Smoke/fire/flames | Struck by/against | Suicide | Homicide |
| --- | --- | --- | --- | --- | --- | --- | --- | --- | --- |
| 2000 | 183 | 306 | 638 | 80 | 80 | 58 | 37 | 1105 | 79 |
| 2001 | 188 | 300 | 605 | 74 | 91 | 58 | 48 | 1279 | 94 |
| 2002 | 223 | 329 | 714 | 82 | 95 | 59 | 40 | 1281 | 99 |
| 2003 | 226 | 305 | 656 | 86 | 78 | 51 | 25 | 1342 | 87 |
| 2004 | 250 | 355 | 668 | 84 | 101 | 67 | 40 | 1293 | 124 |
| 2005 | 268 | 392 | 757 | 100 | 83 | 79 | 37 | 1356 | 124 |
| 2006 | 273 | 460 | 735 | 81 | 115 | 66 | 34 | 1383 | 106 |
| 2007 | 262 | 560 | 772 | 81 | 93 | 58 | 33 | 1420 | 116 |
| 2008 | 303 | 640 | 745 | 87 | 102 | 80 | 49 | 1452 | 130 |
| 2009 | 333 | 640 | 684 | 92 | 114 | 73 | 26 | 1597 | 120 |
| 2010 | 315 | 709 | 676 | 91 | 107 | 76 | 27 | 1648 | 134 |
| 2011 | 355 | 726 | 600 | 82 | 93 | 70 | 22 | 1584 | 128 |
| 2012 | 331 | 797 | 647 | 105 | 100 | 62 | 33 | 1667 | 125 |
| 2013 | 397 | 796 | 606 | 94 | 106 | 73 | 39 | 1718 | 103 |
| 2014 | 393 | 889 | 612 | 99 | 88 | 71 | 36 | 1799 | 126 |
| 2015 | 344 | 1035 | 637 | 94 | 123 | 58 | 38 | 1854 | 142 |
| 2016 | 375 | 1436 | 614 | 88 | 98 | 80 | 33 | 1771 | 112 |
| 2017 | 371 | 1882 | 596 | 97 | 133 | 62 | 28 | 1754 | 138 |
| 2018 | 390 | 1916 | 615 | 99 | 104 | 73 | 45 | 1794 | 119 |
| 2019 | 391 | 1761 | 562 | 85 | 90 | 70 | 36 | 1751 | 146 |
| 2020 | 389 | 2595 | 560 | 105 | 100 | 59 | 28 | 1606 | 126 |
| 2021 | 416 | 2928 | 509 | 97 | 119 | 76 | 39 | 1571 | 112 |
| 2022 | 391 | 2928 | 599 | 96 | 94 | 69 | 20 | 1717 | 105 |
| 2023 | 378 | 2915 | 496 | 99 | 79 | 54 | 18 | 1711 | 75 |

Table A7-2: Number of deaths from all causes, all unintentional causes, other unintentional causes and undetermined causes, aged 45 to 64 years, Canada, from 2000 to 2023

| Year | All causes | All unintentional causes | Other unintentional causes | Undetermined causes |
| --- | --- | --- | --- | --- |
| 2000 | 2917 | 1599 | 217 | 134 |
| 2001 | 3066 | 1560 | 196 | 133 |
| 2002 | 3312 | 1757 | 215 | 175 |
| 2003 | 3322 | 1657 | 230 | 236 |
| 2004 | 3445 | 1762 | 197 | 266 |
| 2005 | 3723 | 1968 | 252 | 275 |
| 2006 | 3758 | 2005 | 241 | 264 |
| 2007 | 3910 | 2099 | 240 | 275 |
| 2008 | 4139 | 2268 | 262 | 289 |
| 2009 | 4267 | 2223 | 261 | 327 |
| 2010 | 4372 | 2257 | 256 | 333 |
| 2011 | 4245 | 2198 | 250 | 335 |
| 2012 | 4474 | 2332 | 257 | 350 |
| 2013 | 4582 | 2403 | 292 | 358 |
| 2014 | 4696 | 2449 | 261 | 322 |
| 2015 | 4821 | 2570 | 241 | 255 |
| 2016 | 4989 | 2984 | 260 | 122 |
| 2017 | 5453 | 3430 | 261 | 131 |
| 2018 | 5602 | 3587 | 345 | 102 |
| 2019 | 5283 | 3272 | 277 | 114 |
| 2020 | 5941 | 4075 | 239 | 134 |
| 2021 | 6402 | 4568 | 384 | 151 |
| 2022 | 6460 | 4491 | 294 | 147 |
| 2023 | 6228 | 4309 | 270 | 133 |

Table A7-3: Crude mortality rates (per 100 000 population) due to leading causes, aged 45 to 64 years, Canada, from 2000 to 2023 (with 95% confidence intervals)

| Year | Falls | Poisonings | Land transport incidents | Suffocation | Drowning | Smoke/fire/ flames | Struck by/against | Suicide | Homicide |
| --- | --- | --- | --- | --- | --- | --- | --- | --- | --- |
| 2000 | 2.6 [2.2, 2.9] | 4.3 [3.8, 4.7] | 8.9 [8.2, 9.6] | 1.1 [0.9, 1.4] | 1.1 [0.9, 1.4] | 0.8 [0.6, 1.0] | 0.5 [0.3, 0.7] | 15.4 [14.5, 16.3] | 1.1 [0.9, 1.3] |
| 2001 | 2.5 [2.2, 2.9] | 4.0 [3.6, 4.5] | 8.2 [7.5, 8.8] | 1.0 [0.8, 1.2] | 1.2 [1.0, 1.5] | 0.8 [0.6, 1.0] | 0.6 [0.5, 0.8] | 17.2 [16.3, 18.2] | 1.3 [1.0, 1.5] |
| 2002 | 2.9 [2.5, 3.3] | 4.3 [3.8, 4.7] | 9.3 [8.6, 10.0] | 1.1 [0.8, 1.3] | 1.2 [1.0, 1.5] | 0.8 [0.6, 1.0] | 0.5 [0.4, 0.7] | 16.7 [15.8, 17.6] | 1.3 [1.0, 1.5] |
| 2003 | 2.8 [2.5, 3.2] | 3.8 [3.4, 4.3] | 8.2 [7.6, 8.9] | 1.1 [0.9, 1.3] | 1.0 [0.8, 1.2] | 0.6 [0.5, 0.8] | 0.3 [0.2, 0.4] | 16.9 [16.0, 17.8] | 1.1 [0.9, 1.3] |
| 2004 | 3.0 [2.7, 3.4] | 4.3 [3.9, 4.8] | 8.1 [7.5, 8.7] | 1.0 [0.8, 1.2] | 1.2 [1.0, 1.5] | 0.8 [0.6, 1.0] | 0.5 [0.3, 0.6] | 15.7 [14.9, 16.6] | 1.5 [1.2, 1.8] |
| 2005 | 3.2 [2.8, 3.5] | 4.6 [4.2, 5.1] | 8.9 [8.3, 9.6] | 1.2 [0.9, 1.4] | 1.0 [0.8, 1.2] | 0.9 [0.7, 1.1] | 0.4 [0.3, 0.6] | 16.0 [15.1, 16.8] | 1.5 [1.2, 1.7] |
| 2006 | 3.1 [2.8, 3.5] | 5.3 [4.8, 5.7] | 8.4 [7.8, 9.0] | 0.9 [0.7, 1.1] | 1.3 [1.1, 1.6] | 0.8 [0.6, 0.9] | 0.4 [0.3, 0.5] | 15.8 [15.0, 16.6] | 1.2 [1.0, 1.4] |
| 2007 | 2.9 [2.6, 3.3] | 6.2 [5.7, 6.7] | 8.6 [8.0, 9.2] | 0.9 [0.7, 1.1] | 1.0 [0.8, 1.2] | 0.6 [0.5, 0.8] | 0.4 [0.2, 0.5] | 15.8 [15.0, 16.6] | 1.3 [1.1, 1.5] |
| 2008 | 3.3 [2.9, 3.6] | 6.9 [6.4, 7.5] | 8.1 [7.5, 8.6] | 0.9 [0.7, 1.1] | 1.1 [0.9, 1.3] | 0.9 [0.7, 1.1] | 0.5 [0.4, 0.7] | 15.7 [14.9, 16.5] | 1.4 [1.2, 1.6] |
| 2009 | 3.5 [3.1, 3.9] | 6.8 [6.2, 7.3] | 7.2 [6.7, 7.8] | 1.0 [0.8, 1.2] | 1.2 [1.0, 1.4] | 0.8 [0.6, 0.9] | 0.3 [0.2, 0.4] | 16.9 [16.0, 17.7] | 1.3 [1.0, 1.5] |
| 2010 | 3.3 [2.9, 3.6] | 7.3 [6.8, 7.9] | 7.0 [6.5, 7.5] | 0.9 [0.7, 1.1] | 1.1 [0.9, 1.3] | 0.8 [0.6, 1.0] | 0.3 [0.2, 0.4] | 17.0 [16.2, 17.8] | 1.4 [1.1, 1.6] |
| 2011 | 3.6 [3.2, 4.0] | 7.4 [6.9, 7.9] | 6.1 [5.6, 6.6] | 0.8 [0.7, 1.0] | 0.9 [0.8, 1.1] | 0.7 [0.5, 0.9] | 0.2 [0.1, 0.3] | 16.1 [15.3, 16.9] | 1.3 [1.1, 1.5] |
| 2012 | 3.3 [3.0, 3.7] | 8.1 [7.5, 8.6] | 6.5 [6.0, 7.0] | 1.1 [0.9, 1.3] | 1.0 [0.8, 1.2] | 0.6 [0.5, 0.8] | 0.3 [0.2, 0.4] | 16.9 [16.1, 17.7] | 1.3 [1.0, 1.5] |
| 2013 | 4.0 [3.6, 4.4] | 8.0 [7.4, 8.6] | 6.1 [5.6, 6.6] | 0.9 [0.8, 1.1] | 1.1 [0.9, 1.3] | 0.7 [0.6, 0.9] | 0.4 [0.3, 0.5] | 17.3 [16.4, 18.1] | 1.0 [0.8, 1.2] |
| 2014 | 3.9 [3.5, 4.3] | 8.9 [8.3, 9.5] | 6.1 [5.6, 6.6] | 1.0 [0.8, 1.2] | 0.9 [0.7, 1.1] | 0.7 [0.5, 0.9] | 0.4 [0.2, 0.5] | 17.9 [17.1, 18.8] | 1.3 [1.0, 1.5] |
| 2015 | 3.4 [3.1, 3.8] | 10.3 [9.6, 10.9] | 6.3 [5.8, 6.8] | 0.9 [0.7, 1.1] | 1.2 [1.0, 1.4] | 0.6 [0.4, 0.7] | 0.4 [0.3, 0.5] | 18.4 [17.5, 19.2] | 1.4 [1.2, 1.6] |
| 2016 | 3.7 [3.3, 4.1] | 14.2 [13.4, 14.9] | 6.1 [5.6, 6.5] | 0.9 [0.7, 1.0] | 1.0 [0.8, 1.2] | 0.8 [0.6, 1.0] | 0.3 [0.2, 0.4] | 17.5 [16.7, 18.3] | 1.1 [0.9, 1.3] |
| 2017 | 3.7 [3.3, 4.0] | 18.5 [17.7, 19.4] | 5.9 [5.4, 6.3] | 1.0 [0.8, 1.1] | 1.3 [1.1, 1.5] | 0.6 [0.5, 0.8] | 0.3 [0.2, 0.4] | 17.3 [16.5, 18.1] | 1.4 [1.1, 1.6] |
| 2018 | 3.8 [3.5, 4.2] | 18.8 [18.0, 19.7] | 6.0 [5.6, 6.5] | 1.0 [0.8, 1.2] | 1.0 [0.8, 1.2] | 0.7 [0.6, 0.9] | 0.4 [0.3, 0.6] | 17.6 [16.8, 18.5] | 1.2 [1.0, 1.4] |
| 2019 | 3.8 [3.5, 4.2] | 17.3 [16.5, 18.1] | 5.5 [5.1, 6.0] | 0.8 [0.7, 1.0] | 0.9 [0.7, 1.1] | 0.7 [0.5, 0.8] | 0.4 [0.2, 0.5] | 17.2 [16.4, 18.0] | 1.4 [1.2, 1.7] |
| 2020 | 3.8 [3.5, 4.2] | 25.6 [24.6, 26.6] | 5.5 [5.1, 6.0] | 1.0 [0.8, 1.2] | 1.0 [0.8, 1.2] | 0.6 [0.4, 0.7] | 0.3 [0.2, 0.4] | 15.8 [15.1, 16.6] | 1.2 [1.0, 1.5] |
| 2021 | 4.1 [3.7, 4.5] | 29.0 [27.9, 30.0] | 5.0 [4.6, 5.5] | 1.0 [0.8, 1.2] | 1.2 [1.0, 1.4] | 0.8 [0.6, 0.9] | 0.4 [0.3, 0.5] | 15.5 [14.8, 16.3] | 1.1 [0.9, 1.3] |
| 2022 | 3.9 [3.5, 4.3] | 29.0 [27.9, 30.0] | 5.9 [5.5, 6.4] | 1.0 [0.8, 1.1] | 0.9 [0.7, 1.1] | 0.7 [0.5, 0.8] | 0.2 [0.1, 0.3] | 17.0 [16.2, 17.8] | 1.0 [0.8, 1.2] |
| 2023 | 3.7 [3.4, 4.1] | 28.8 [27.8, 29.9] | 4.9 [4.5, 5.3] | 1.0 [0.8, 1.2] | 0.8 [0.6, 1.0] | 0.5 [0.4, 0.7] | 0.2 [0.1, 0.3] | 16.9 [16.1, 17.7] | 0.7 [0.6, 0.9] |

Table A7-4: Crude mortality rates (per 100 000 population) from all causes, all unintentional causes, other unintentional causes and undetermined causes, aged 45 to 64 years, Canada, from 2000 to 2023 (with 95% confidence intervals)

| Year | All causes | All unintentional causes | Other unintentional causes | Undetermined causes |
| --- | --- | --- | --- | --- |
| 2000 | 40.7 [39.2, 42.2] | 22.3 [21.2, 23.4] | 3.0 [2.6, 3.4] | 1.9 [1.6, 2.2] |
| 2001 | 41.4 [39.9, 42.8] | 21.0 [20.0, 22.1] | 2.6 [2.3, 3.0] | 1.8 [1.5, 2.1] |
| 2002 | 43.1 [41.6, 44.6] | 22.9 [21.8, 23.9] | 2.8 [2.4, 3.2] | 2.3 [1.9, 2.6] |
| 2003 | 41.8 [40.4, 43.2] | 20.8 [19.8, 21.8] | 2.9 [2.5, 3.3] | 3.0 [2.6, 3.3] |
| 2004 | 41.9 [40.5, 43.3] | 21.4 [20.4, 22.5] | 2.4 [2.1, 2.7] | 3.2 [2.8, 3.6] |
| 2005 | 43.9 [42.5, 45.3] | 23.2 [22.2, 24.2] | 3.0 [2.6, 3.3] | 3.2 [2.9, 3.6] |
| 2006 | 43.0 [41.6, 44.3] | 22.9 [21.9, 23.9] | 2.8 [2.4, 3.1] | 3.0 [2.7, 3.4] |
| 2007 | 43.5 [42.1, 44.8] | 23.3 [22.3, 24.3] | 2.7 [2.3, 3.0] | 3.1 [2.7, 3.4] |
| 2008 | 44.8 [43.4, 46.1] | 24.5 [23.5, 25.5] | 2.8 [2.5, 3.2] | 3.1 [2.8, 3.5] |
| 2009 | 45.0 [43.7, 46.4] | 23.5 [22.5, 24.4] | 2.8 [2.4, 3.1] | 3.5 [3.1, 3.8] |
| 2010 | 45.2 [43.8, 46.5] | 23.3 [22.4, 24.3] | 2.6 [2.3, 3.0] | 3.4 [3.1, 3.8] |
| 2011 | 43.3 [42.0, 44.6] | 22.4 [21.5, 23.3] | 2.5 [2.2, 2.9] | 3.4 [3.0, 3.8] |
| 2012 | 45.3 [43.9, 46.6] | 23.6 [22.6, 24.5] | 2.6 [2.3, 2.9] | 3.5 [3.2, 3.9] |
| 2013 | 46.0 [44.7, 47.4] | 24.1 [23.2, 25.1] | 2.9 [2.6, 3.3] | 3.6 [3.2, 4.0] |
| 2014 | 46.8 [45.5, 48.2] | 24.4 [23.5, 25.4] | 2.6 [2.3, 2.9] | 3.2 [2.9, 3.6] |
| 2015 | 47.8 [46.5, 49.2] | 25.5 [24.5, 26.5] | 2.4 [2.1, 2.7] | 2.5 [2.2, 2.8] |
| 2016 | 49.2 [47.8, 50.6] | 29.4 [28.4, 30.5] | 2.6 [2.3, 2.9] | 1.2 [1.0, 1.4] |
| 2017 | 53.7 [52.2, 55.1] | 33.8 [32.6, 34.9] | 2.6 [2.3, 2.9] | 1.3 [1.1, 1.5] |
| 2018 | 55.1 [53.6, 56.5] | 35.3 [34.1, 36.4] | 3.4 [3.0, 3.8] | 1.0 [0.8, 1.2] |
| 2019 | 52.0 [50.6, 53.4] | 32.2 [31.1, 33.3] | 2.7 [2.4, 3.0] | 1.1 [0.9, 1.3] |
| 2020 | 58.5 [57.1, 60.0] | 40.2 [38.9, 41.4] | 2.4 [2.1, 2.7] | 1.3 [1.1, 1.5] |
| 2021 | 63.3 [61.8, 64.9] | 45.2 [43.9, 46.5] | 3.8 [3.4, 4.2] | 1.5 [1.3, 1.7] |
| 2022 | 64.0 [62.4, 65.5] | 44.5 [43.2, 45.8] | 2.9 [2.6, 3.2] | 1.5 [1.2, 1.7] |
| 2023 | 61.6 [60.0, 63.1] | 42.6 [41.3, 43.9] | 2.7 [2.4, 3.0] | 1.3 [1.1, 1.5] |

Table A8-1: Number of deaths due to leading causes, aged 65 to 79 years, Canada, from 2000 to 2023

| Year | Falls | Poisonings | Land transport incidents | Suffocation | Drowning | Smoke/fire/flames | Struck by/against | Suicide | Homicide |
| --- | --- | --- | --- | --- | --- | --- | --- | --- | --- |
| 2000 | 388 | 46 | 394 | 98 | 36 | 45 | 19 | 322 | 32 |
| 2001 | 417 | 58 | 362 | 76 | 40 | 49 | 11 | 323 | 27 |
| 2002 | 409 | 61 | 358 | 106 | 39 | 35 | 10 | 328 | 31 |
| 2003 | 476 | 49 | 338 | 102 | 43 | 53 | 13 | 334 | 22 |
| 2004 | 476 | 61 | 367 | 76 | 38 | 43 | 8 | 318 | 35 |
| 2005 | 552 | 49 | 354 | 97 | 37 | 42 | 15 | 349 | 41 |
| 2006 | 590 | 62 | 354 | 94 | 40 | 31 | 21 | 369 | 22 |
| 2007 | 613 | 98 | 326 | 109 | 44 | 36 | 20 | 325 | 29 |
| 2008 | 635 | 92 | 308 | 103 | 35 | 59 | 12 | 360 | 27 |
| 2009 | 701 | 92 | 290 | 90 | 40 | 42 | 16 | 347 | 24 |
| 2010 | 773 | 89 | 333 | 91 | 38 | 45 | 18 | 377 | 26 |
| 2011 | 813 | 94 | 329 | 124 | 42 | 39 | 21 | 395 | 33 |
| 2012 | 776 | 102 | 324 | 102 | 62 | 39 | 13 | 401 | 31 |
| 2013 | 833 | 108 | 306 | 109 | 61 | 55 | 12 | 432 | 26 |
| 2014 | 906 | 115 | 346 | 95 | 62 | 45 | 16 | 466 | 26 |
| 2015 | 895 | 130 | 321 | 102 | 50 | 57 | 17 | 514 | 33 |
| 2016 | 984 | 170 | 346 | 101 | 43 | 57 | 22 | 537 | 28 |
| 2017 | 1003 | 207 | 296 | 127 | 63 | 47 | 15 | 503 | 41 |
| 2018 | 1106 | 228 | 342 | 105 | 63 | 58 | 20 | 561 | 32 |
| 2019 | 1183 | 241 | 383 | 117 | 59 | 55 | 16 | 552 | 31 |
| 2020 | 1323 | 291 | 302 | 145 | 71 | 62 | 27 | 578 | 37 |
| 2021 | 1394 | 367 | 302 | 157 | 57 | 63 | 15 | 568 | 25 |
| 2022 | 1619 | 443 | 354 | 171 | 81 | 77 | 21 | 673 | 46 |
| 2023 | 1569 | 447 | 315 | 169 | 88 | 63 | 25 | 618 | 22 |

Table A8-2: Number of deaths from all causes, all unintentional causes, other unintentional causes and undetermined causes, aged 65 to 79 years, Canada, from 2000 to 2023

| Year | All causes | All unintentional causes | Other unintentional causes | Undetermined causes |
| --- | --- | --- | --- | --- |
| 2000 | 1753 | 1351 | 325 | 48 |
| 2001 | 1726 | 1338 | 325 | 38 |
| 2002 | 1784 | 1381 | 363 | 44 |
| 2003 | 1851 | 1445 | 371 | 50 |
| 2004 | 1766 | 1373 | 304 | 40 |
| 2005 | 1906 | 1472 | 326 | 44 |
| 2006 | 1915 | 1485 | 293 | 39 |
| 2007 | 1937 | 1546 | 300 | 37 |
| 2008 | 1967 | 1534 | 290 | 46 |
| 2009 | 1944 | 1531 | 260 | 42 |
| 2010 | 2096 | 1638 | 251 | 55 |
| 2011 | 2175 | 1707 | 245 | 40 |
| 2012 | 2112 | 1624 | 206 | 56 |
| 2013 | 2263 | 1751 | 267 | 54 |
| 2014 | 2386 | 1846 | 261 | 48 |
| 2015 | 2464 | 1879 | 307 | 38 |
| 2016 | 2613 | 2014 | 291 | 34 |
| 2017 | 2630 | 2048 | 290 | 38 |
| 2018 | 2946 | 2329 | 407 | 24 |
| 2019 | 3039 | 2414 | 360 | 42 |
| 2020 | 3261 | 2597 | 376 | 49 |
| 2021 | 3585 | 2949 | 594 | 43 |
| 2022 | 3997 | 3225 | 459 | 53 |
| 2023 | 3817 | 3121 | 445 | 56 |

Table A8-3: Crude mortality rates (per 100 000 population) due to leading causes, aged 65 to 79 years, Canada, from 2000 to 2023 (with 95% confidence intervals)

| Year | Falls | Poisonings | Land transport incidents | Suffocation | Drowning | Smoke/fire/ flames | Struck by/against | Suicide | Homicide |
| --- | --- | --- | --- | --- | --- | --- | --- | --- | --- |
| 2000 | 13.1 [11.8, 14.5] | 1.6 [1.1, 2.0] | 13.3 [12.0, 14.7] | 3.3 [2.7, 4.0] | 1.2 [0.8, 1.6] | 1.5 [1.1, 2.0] | 0.6 [0.4, 0.9] | 10.9 [9.7, 12.1] | 1.1 [0.7, 1.5] |
| 2001 | 14.0 [12.6, 15.3] | 1.9 [1.4, 2.4] | 12.1 [10.9, 13.4] | 2.5 [2.0, 3.1] | 1.3 [0.9, 1.8] | 1.6 [1.2, 2.1] | 0.4 [0.2, 0.6] | 10.8 [9.7, 12.0] | 0.9 [0.6, 1.2] |
| 2002 | 13.6 [12.3, 14.9] | 2.0 [1.5, 2.5] | 11.9 [10.7, 13.2] | 3.5 [2.9, 4.2] | 1.3 [0.9, 1.7] | 1.2 [0.8, 1.6] | 0.3 [0.1, 0.5] | 10.9 [9.7, 12.1] | 1.0 [0.7, 1.4] |
| 2003 | 15.7 [14.3, 17.1] | 1.6 [1.2, 2.1] | 11.1 [10.0, 12.3] | 3.4 [2.7, 4.0] | 1.4 [1.0, 1.8] | 1.7 [1.3, 2.2] | 0.4 [0.2, 0.7] | 11.0 [9.8, 12.2] | 0.7 [0.4, 1.0] |
| 2004 | 15.5 [14.1, 16.9] | 2.0 [1.5, 2.5] | 12.0 [10.7, 13.2] | 2.5 [1.9, 3.0] | 1.2 [0.8, 1.6] | 1.4 [1.0, 1.8] | 0.3 [0.1, 0.4] | 10.4 [9.2, 11.5] | 1.1 [0.8, 1.5] |
| 2005 | 17.8 [16.3, 19.3] | 1.6 [1.1, 2.0] | 11.4 [10.2, 12.6] | 3.1 [2.5, 3.8] | 1.2 [0.8, 1.6] | 1.4 [0.9, 1.8] | 0.5 [0.2, 0.7] | 11.3 [10.1, 12.4] | 1.3 [0.9, 1.7] |
| 2006 | 18.7 [17.2, 20.2] | 2.0 [1.5, 2.5] | 11.2 [10.0, 12.4] | 3.0 [2.4, 3.6] | 1.3 [0.9, 1.7] | 1.0 [0.6, 1.3] | 0.7 [0.4, 0.9] | 11.7 [10.5, 12.9] | 0.7 [0.4, 1.0] |
| 2007 | 19.0 [17.5, 20.5] | 3.0 [2.4, 3.6] | 10.1 [9.0, 11.2] | 3.4 [2.7, 4.0] | 1.4 [1.0, 1.8] | 1.1 [0.8, 1.5] | 0.6 [0.3, 0.9] | 10.1 [9.0, 11.2] | 0.9 [0.6, 1.2] |
| 2008 | 19.2 [17.7, 20.7] | 2.8 [2.2, 3.4] | 9.3 [8.3, 10.4] | 3.1 [2.5, 3.7] | 1.1 [0.7, 1.4] | 1.8 [1.3, 2.2] | 0.4 [0.2, 0.6] | 10.9 [9.8, 12.0] | 0.8 [0.5, 1.1] |
| 2009 | 20.6 [19.1, 22.2] | 2.7 [2.2, 3.3] | 8.5 [7.6, 9.5] | 2.6 [2.1, 3.2] | 1.2 [0.8, 1.5] | 1.2 [0.9, 1.6] | 0.5 [0.2, 0.7] | 10.2 [9.1, 11.3] | 0.7 [0.4, 1.0] |
| 2010 | 22.1 [20.6, 23.7] | 2.5 [2.0, 3.1] | 9.5 [8.5, 10.6] | 2.6 [2.1, 3.1] | 1.1 [0.7, 1.4] | 1.3 [0.9, 1.7] | 0.5 [0.3, 0.8] | 10.8 [9.7, 11.9] | 0.7 [0.5, 1.0] |
| 2011 | 22.6 [21.0, 24.1] | 2.6 [2.1, 3.1] | 9.1 [8.1, 10.1] | 3.4 [2.8, 4.0] | 1.2 [0.8, 1.5] | 1.1 [0.7, 1.4] | 0.6 [0.3, 0.8] | 11.0 [9.9, 12.0] | 0.9 [0.6, 1.2] |
| 2012 | 20.6 [19.2, 22.1] | 2.7 [2.2, 3.2] | 8.6 [7.7, 9.5] | 2.7 [2.2, 3.2] | 1.6 [1.2, 2.1] | 1.0 [0.7, 1.4] | 0.3 [0.2, 0.5] | 10.7 [9.6, 11.7] | 0.8 [0.5, 1.1] |
| 2013 | 21.2 [19.8, 22.6] | 2.7 [2.2, 3.3] | 7.8 [6.9, 8.7] | 2.8 [2.3, 3.3] | 1.6 [1.2, 1.9] | 1.4 [1.0, 1.8] | 0.3 [0.1, 0.5] | 11.0 [10.0, 12.0] | 0.7 [0.4, 0.9] |
| 2014 | 22.2 [20.7, 23.6] | 2.8 [2.3, 3.3] | 8.5 [7.6, 9.4] | 2.3 [1.9, 2.8] | 1.5 [1.1, 1.9] | 1.1 [0.8, 1.4] | 0.4 [0.2, 0.6] | 11.4 [10.4, 12.4] | 0.6 [0.4, 0.9] |
| 2015 | 21.1 [19.7, 22.5] | 3.1 [2.5, 3.6] | 7.6 [6.7, 8.4] | 2.4 [1.9, 2.9] | 1.2 [0.9, 1.5] | 1.3 [1.0, 1.7] | 0.4 [0.2, 0.6] | 12.1 [11.1, 13.2] | 0.8 [0.5, 1.0] |
| 2016 | 22.3 [20.9, 23.7] | 3.9 [3.3, 4.4] | 7.9 [7.0, 8.7] | 2.3 [1.8, 2.7] | 1.0 [0.7, 1.3] | 1.3 [1.0, 1.6] | 0.5 [0.3, 0.7] | 12.2 [11.2, 13.2] | 0.6 [0.4, 0.9] |
| 2017 | 21.9 [20.5, 23.2] | 4.5 [3.9, 5.1] | 6.5 [5.7, 7.2] | 2.8 [2.3, 3.3] | 1.4 [1.0, 1.7] | 1.0 [0.7, 1.3] | 0.3 [0.2, 0.5] | 11.0 [10.0, 11.9] | 0.9 [0.6, 1.2] |
| 2018 | 23.2 [21.9, 24.6] | 4.8 [4.2, 5.4] | 7.2 [6.4, 7.9] | 2.2 [1.8, 2.6] | 1.3 [1.0, 1.6] | 1.2 [0.9, 1.5] | 0.4 [0.2, 0.6] | 11.8 [10.8, 12.8] | 0.7 [0.4, 0.9] |
| 2019 | 23.9 [22.5, 25.2] | 4.9 [4.2, 5.5] | 7.7 [6.9, 8.5] | 2.4 [1.9, 2.8] | 1.2 [0.9, 1.5] | 1.1 [0.8, 1.4] | 0.3 [0.2, 0.5] | 11.1 [10.2, 12.1] | 0.6 [0.4, 0.8] |
| 2020 | 25.6 [24.3, 27.0] | 5.6 [5.0, 6.3] | 5.9 [5.2, 6.5] | 2.8 [2.4, 3.3] | 1.4 [1.1, 1.7] | 1.2 [0.9, 1.5] | 0.5 [0.3, 0.7] | 11.2 [10.3, 12.1] | 0.7 [0.5, 0.9] |
| 2021 | 26.1 [24.7, 27.5] | 6.9 [6.2, 7.6] | 5.7 [5.0, 6.3] | 2.9 [2.5, 3.4] | 1.1 [0.8, 1.3] | 1.2 [0.9, 1.5] | 0.3 [0.1, 0.4] | 10.6 [9.8, 11.5] | 0.5 [0.3, 0.7] |
| 2022 | 29.2 [27.8, 30.6] | 8.0 [7.2, 8.7] | 6.4 [5.7, 7.0] | 3.1 [2.6, 3.5] | 1.5 [1.1, 1.8] | 1.4 [1.1, 1.7] | 0.4 [0.2, 0.5] | 12.1 [11.2, 13.0] | 0.8 [0.6, 1.1] |
| 2023 | 27.3 [25.9, 28.6] | 7.8 [7.1, 8.5] | 5.5 [4.9, 6.1] | 2.9 [2.5, 3.4] | 1.5 [1.2, 1.9] | 1.1 [0.8, 1.4] | 0.4 [0.3, 0.6] | 10.7 [9.9, 11.6] | 0.4 [0.2, 0.5] |

Table A8-4: Crude mortality rates (per 100 000 population) from all causes, all unintentional causes, other unintentional causes and undetermined causes, aged 65 to 79 years, Canada, from 2000 to 2023 (with 95% confidence intervals)

| Year | All causes | All unintentional causes | Other unintentional causes | Undetermined causes |
| --- | --- | --- | --- | --- |
| 2000 | 59.4 [56.6, 62.2] | 45.8 [43.3, 48.2] | 11.0 [9.8, 12.2] | 1.6 [1.2, 2.1] |
| 2001 | 57.9 [55.2, 60.6] | 44.9 [42.5, 47.3] | 10.9 [9.7, 12.1] | 1.3 [0.9, 1.7] |
| 2002 | 59.4 [56.6, 62.2] | 46.0 [43.6, 48.4] | 12.1 [10.8, 13.3] | 1.5 [1.0, 1.9] |
| 2003 | 61.0 [58.3, 63.8] | 47.7 [45.2, 50.1] | 12.2 [11.0, 13.5] | 1.6 [1.2, 2.1] |
| 2004 | 57.6 [54.9, 60.3] | 44.8 [42.4, 47.2] | 9.9 [8.8, 11.0] | 1.3 [0.9, 1.7] |
| 2005 | 61.5 [58.7, 64.2] | 47.5 [45.0, 49.9] | 10.5 [9.4, 11.7] | 1.4 [1.0, 1.8] |
| 2006 | 60.6 [57.9, 63.3] | 47.0 [44.6, 49.4] | 9.3 [8.2, 10.3] | 1.2 [0.8, 1.6] |
| 2007 | 60.1 [57.4, 62.7] | 47.9 [45.5, 50.3] | 9.3 [8.2, 10.4] | 1.1 [0.8, 1.5] |
| 2008 | 59.5 [56.9, 62.1] | 46.4 [44.1, 48.7] | 8.8 [7.8, 9.8] | 1.4 [1.0, 1.8] |
| 2009 | 57.2 [54.7, 59.7] | 45.0 [42.8, 47.3] | 7.6 [6.7, 8.6] | 1.2 [0.9, 1.6] |
| 2010 | 60.0 [57.5, 62.6] | 46.9 [44.6, 49.2] | 7.2 [6.3, 8.1] | 1.6 [1.2, 2.0] |
| 2011 | 60.4 [57.8, 62.9] | 47.4 [45.1, 49.6] | 6.8 [5.9, 7.6] | 1.1 [0.8, 1.5] |
| 2012 | 56.1 [53.7, 58.5] | 43.1 [41.1, 45.2] | 5.5 [4.7, 6.2] | 1.5 [1.1, 1.9] |
| 2013 | 57.6 [55.2, 60.0] | 44.6 [42.5, 46.7] | 6.8 [6.0, 7.6] | 1.4 [1.0, 1.7] |
| 2014 | 58.4 [56.0, 60.7] | 45.2 [43.1, 47.2] | 6.4 [5.6, 7.2] | 1.2 [0.8, 1.5] |
| 2015 | 58.1 [55.8, 60.4] | 44.3 [42.3, 46.3] | 7.2 [6.4, 8.0] | 0.9 [0.6, 1.2] |
| 2016 | 59.3 [57.0, 61.6] | 45.7 [43.7, 47.7] | 6.6 [5.8, 7.4] | 0.8 [0.5, 1.0] |
| 2017 | 57.4 [55.2, 59.6] | 44.7 [42.8, 46.6] | 6.3 [5.6, 7.1] | 0.8 [0.6, 1.1] |
| 2018 | 61.9 [59.6, 64.1] | 48.9 [46.9, 50.9] | 8.5 [7.7, 9.4] | 0.5 [0.3, 0.7] |
| 2019 | 61.3 [59.1, 63.5] | 48.7 [46.7, 50.6] | 7.3 [6.5, 8.0] | 0.8 [0.6, 1.1] |
| 2020 | 63.2 [61.0, 65.4] | 50.3 [48.4, 52.3] | 7.3 [6.6, 8.0] | 0.9 [0.7, 1.2] |
| 2021 | 67.1 [64.9, 69.3] | 55.2 [53.2, 57.2] | 11.1 [10.2, 12.0] | 0.8 [0.6, 1.0] |
| 2022 | 72.1 [69.8, 74.3] | 58.1 [56.1, 60.1] | 8.3 [7.5, 9.0] | 1.0 [0.7, 1.2] |
| 2023 | 66.4 [64.3, 68.5] | 54.3 [52.4, 56.2] | 7.7 [7.0, 8.5] | 1.0 [0.7, 1.2] |

Table A9-1: Number of deaths due to leading causes, aged 80 years and older, Canada, from 2000 to 2023

| Year | Falls | Poisonings | Land transport incidents | Suffocation | Drowning | Smoke/fire/flames | Struck by/against | Suicide | Homicide |
| --- | --- | --- | --- | --- | --- | --- | --- | --- | --- |
| 2000 | 887 | 23 | 176 | 138 | 12 | 45 | 4 | 82 | 14 |
| 2001 | 1018 | 23 | 168 | 119 | 14 | 31 | 9 | 77 | 2 |
| 2002 | 1004 | 32 | 216 | 124 | 20 | 22 | 1 | 111 | 10 |
| 2003 | 1160 | 29 | 182 | 138 | 16 | 17 | 6 | 110 | 14 |
| 2004 | 1389 | 32 | 189 | 138 | 18 | 27 | 4 | 110 | 15 |
| 2005 | 1371 | 34 | 217 | 125 | 18 | 29 | 5 | 109 | 8 |
| 2006 | 1646 | 38 | 178 | 147 | 16 | 22 | 6 | 124 | 11 |
| 2007 | 1748 | 43 | 196 | 182 | 21 | 36 | 10 | 132 | 8 |
| 2008 | 2080 | 51 | 188 | 164 | 26 | 29 | 11 | 132 | 9 |
| 2009 | 2500 | 39 | 199 | 179 | 9 | 43 | 12 | 146 | 11 |
| 2010 | 2874 | 44 | 187 | 185 | 25 | 36 | 12 | 147 | 14 |
| 2011 | 2999 | 35 | 200 | 219 | 23 | 28 | 7 | 144 | 20 |
| 2012 | 3291 | 36 | 175 | 206 | 22 | 35 | 8 | 139 | 14 |
| 2013 | 3378 | 34 | 210 | 170 | 23 | 35 | 12 | 192 | 24 |
| 2014 | 3490 | 43 | 149 | 160 | 26 | 56 | 9 | 179 | 13 |
| 2015 | 3432 | 42 | 214 | 161 | 16 | 27 | 10 | 184 | 16 |
| 2016 | 3489 | 41 | 219 | 160 | 29 | 21 | 10 | 178 | 21 |
| 2017 | 3755 | 53 | 174 | 170 | 23 | 30 | 14 | 193 | 14 |
| 2018 | 4179 | 44 | 212 | 211 | 27 | 21 | 13 | 172 | 14 |
| 2019 | 4656 | 46 | 177 | 241 | 24 | 24 | 9 | 189 | 16 |
| 2020 | 5006 | 31 | 136 | 200 | 33 | 31 | 12 | 194 | 15 |
| 2021 | 5300 | 45 | 164 | 236 | 25 | 27 | 15 | 186 | 10 |
| 2022 | 6008 | 58 | 184 | 321 | 34 | 39 | 20 | 215 | 21 |
| 2023 | 6011 | 52 | 196 | 327 | 35 | 21 | 20 | 175 | 15 |

Table A9-2: Number of deaths from all causes, all unintentional causes, other unintentional causes and undetermined causes, aged 80 years and older, Canada, from 2000 to 2023

| Year | All causes | All unintentional causes | Other unintentional causes | Undetermined causes |
| --- | --- | --- | --- | --- |
| 2000 | 2566 | 2456 | 1171 | 14 |
| 2001 | 2554 | 2466 | 1084 | 9 |
| 2002 | 2868 | 2737 | 1318 | 10 |
| 2003 | 3042 | 2899 | 1351 | 19 |
| 2004 | 3094 | 2946 | 1149 | 23 |
| 2005 | 3157 | 3025 | 1226 | 15 |
| 2006 | 3341 | 3193 | 1140 | 13 |
| 2007 | 3430 | 3272 | 1036 | 18 |
| 2008 | 3713 | 3557 | 1008 | 15 |
| 2009 | 4001 | 3833 | 852 | 11 |
| 2010 | 4418 | 4242 | 879 | 15 |
| 2011 | 4572 | 4393 | 882 | 15 |
| 2012 | 4767 | 4599 | 826 | 15 |
| 2013 | 4920 | 4693 | 831 | 11 |
| 2014 | 5057 | 4847 | 914 | 18 |
| 2015 | 5033 | 4816 | 914 | 17 |
| 2016 | 5226 | 5010 | 1041 | 17 |
| 2017 | 5478 | 5256 | 1037 | 15 |
| 2018 | 5936 | 5731 | 1024 | 19 |
| 2019 | 6481 | 6255 | 1078 | 21 |
| 2020 | 6733 | 6498 | 1049 | 26 |
| 2021 | 7281 | 7069 | 1257 | 16 |
| 2022 | 8020 | 7774 | 1110 | 10 |
| 2023 | 8025 | 7810 | 1148 | 25 |

Table A9-3: Crude mortality rates (per 100 000 population) due to leading causes, aged 80 years and older, Canada, from 2000 to 2023 (with 95% confidence intervals)

| Year | Falls | Poisonings | Land transport incidents | Suffocation | Drowning | Smoke/fire/ flames | Struck by/against | Suicide | Homicide |
| --- | --- | --- | --- | --- | --- | --- | --- | --- | --- |
| 2000 | 98.6 [92.1, 105.1] | 2.6 [1.5, 3.6] | 19.6 [16.7, 22.5] | 15.3 [12.8, 17.9] | 1.3 [0.6, 2.1] | 5.0 [3.5, 6.5] | 0.4 [0.1, 1.1] | 9.1 [7.1, 11.1] | 1.6 [0.7, 2.4] |
| 2001 | 108.1 [101.5, 114.8] | 2.4 [1.4, 3.4] | 17.8 [15.1, 20.5] | 12.6 [10.4, 14.9] | 1.5 [0.7, 2.3] | 3.3 [2.1, 4.5] | 1.0 [0.3, 1.6] | 8.2 [6.4, 10.0] | 0.2 [0.0, 0.8] |
| 2002 | 101.8 [95.5, 108.1] | 3.2 [2.1, 4.4] | 21.9 [19.0, 24.8] | 12.6 [10.4, 14.8] | 2.0 [1.1, 2.9] | 2.2 [1.3, 3.2] | 0.1 [0.0, 0.6] | 11.3 [9.2, 13.3] | 1.0 [0.4, 1.6] |
| 2003 | 112.8 [106.3, 119.3] | 2.8 [1.8, 3.8] | 17.7 [15.1, 20.3] | 13.4 [11.2, 15.7] | 1.6 [0.8, 2.3] | 1.7 [0.9, 2.4] | 0.6 [0.1, 1.1] | 10.7 [8.7, 12.7] | 1.4 [0.6, 2.1] |
| 2004 | 129.8 [123.0, 136.7] | 3.0 [2.0, 4.0] | 17.7 [15.1, 20.2] | 12.9 [10.7, 15.1] | 1.7 [0.9, 2.5] | 2.5 [1.6, 3.5] | 0.4 [0.1, 1.0] | 10.3 [8.4, 12.2] | 1.4 [0.7, 2.1] |
| 2005 | 123.2 [116.7, 129.8] | 3.1 [2.0, 4.1] | 19.5 [16.9, 22.1] | 11.2 [9.3, 13.2] | 1.6 [0.9, 2.4] | 2.6 [1.7, 3.6] | 0.4 [0.1, 0.8] | 9.8 [8.0, 11.6] | 0.7 [0.2, 1.2] |
| 2006 | 141.9 [135.1, 148.8] | 3.3 [2.2, 4.3] | 15.3 [13.1, 17.6] | 12.7 [10.6, 14.7] | 1.4 [0.7, 2.1] | 1.9 [1.1, 2.7] | 0.5 [0.1, 0.9] | 10.7 [8.8, 12.6] | 0.9 [0.4, 1.5] |
| 2007 | 146.2 [139.3, 153.0] | 3.6 [2.5, 4.7] | 16.4 [14.1, 18.7] | 15.2 [13.0, 17.4] | 1.8 [1.0, 2.5] | 3.0 [2.0, 4.0] | 0.8 [0.3, 1.4] | 11.0 [9.2, 12.9] | 0.7 [0.2, 1.1] |
| 2008 | 168.6 [161.4, 175.9] | 4.1 [3.0, 5.3] | 15.2 [13.1, 17.4] | 13.3 [11.3, 15.3] | 2.1 [1.3, 2.9] | 2.4 [1.5, 3.2] | 0.9 [0.4, 1.4] | 10.7 [8.9, 12.5] | 0.7 [0.3, 1.2] |
| 2009 | 196.9 [189.2, 204.6] | 3.1 [2.1, 4.0] | 15.7 [13.5, 17.8] | 14.1 [12.0, 16.2] | 0.7 [0.2, 1.2] | 3.4 [2.4, 4.4] | 0.9 [0.4, 1.5] | 11.5 [9.6, 13.4] | 0.9 [0.4, 1.4] |
| 2010 | 219.1 [211.1, 227.2] | 3.4 [2.4, 4.3] | 14.3 [12.2, 16.3] | 14.1 [12.1, 16.1] | 1.9 [1.2, 2.7] | 2.7 [1.8, 3.6] | 0.9 [0.4, 1.4] | 11.2 [9.4, 13.0] | 1.1 [0.5, 1.6] |
| 2011 | 221.9 [214.0, 229.9] | 2.6 [1.7, 3.4] | 14.8 [12.7, 16.9] | 16.2 [14.1, 18.4] | 1.7 [1.0, 2.4] | 2.1 [1.3, 2.8] | 0.5 [0.1, 0.9] | 10.7 [8.9, 12.4] | 1.5 [0.8, 2.1] |
| 2012 | 236.6 [228.5, 244.7] | 2.6 [1.7, 3.4] | 12.6 [10.7, 14.4] | 14.8 [12.8, 16.8] | 1.6 [0.9, 2.2] | 2.5 [1.7, 3.3] | 0.6 [0.2, 1.0] | 10.0 [8.3, 11.7] | 1.0 [0.5, 1.5] |
| 2013 | 237.2 [229.2, 245.2] | 2.4 [1.6, 3.2] | 14.7 [12.7, 16.7] | 11.9 [10.1, 13.7] | 1.6 [1.0, 2.3] | 2.5 [1.6, 3.3] | 0.8 [0.4, 1.3] | 13.5 [11.6, 15.4] | 1.7 [1.0, 2.4] |
| 2014 | 240.0 [232.1, 248.0] | 3.0 [2.1, 3.8] | 10.2 [8.6, 11.9] | 11.0 [9.3, 12.7] | 1.8 [1.1, 2.5] | 3.9 [2.8, 4.9] | 0.6 [0.2, 1.0] | 12.3 [10.5, 14.1] | 0.9 [0.4, 1.4] |
| 2015 | 232.1 [224.4, 239.9] | 2.8 [2.0, 3.7] | 14.5 [12.5, 16.4] | 10.9 [9.2, 12.6] | 1.1 [0.6, 1.6] | 1.8 [1.1, 2.5] | 0.7 [0.3, 1.1] | 12.4 [10.6, 14.2] | 1.1 [0.6, 1.6] |
| 2016 | 230.5 [222.8, 238.1] | 2.7 [1.9, 3.5] | 14.5 [12.6, 16.4] | 10.6 [8.9, 12.2] | 1.9 [1.2, 2.6] | 1.4 [0.8, 2.0] | 0.7 [0.3, 1.1] | 11.8 [10.0, 13.5] | 1.4 [0.8, 2.0] |
| 2017 | 243.4 [235.6, 251.2] | 3.4 [2.5, 4.4] | 11.3 [9.6, 13.0] | 11.0 [9.4, 12.7] | 1.5 [0.9, 2.1] | 1.9 [1.2, 2.6] | 0.9 [0.4, 1.4] | 12.5 [10.7, 14.3] | 0.9 [0.4, 1.4] |
| 2018 | 265.4 [257.4, 273.5] | 2.8 [2.0, 3.6] | 13.5 [11.7, 15.3] | 13.4 [11.6, 15.2] | 1.7 [1.1, 2.4] | 1.3 [0.8, 1.9] | 0.8 [0.4, 1.3] | 10.9 [9.3, 12.6] | 0.9 [0.4, 1.4] |
| 2019 | 288.5 [280.2, 296.8] | 2.9 [2.0, 3.7] | 11.0 [9.4, 12.6] | 14.9 [13.0, 16.8] | 1.5 [0.9, 2.1] | 1.5 [0.9, 2.1] | 0.6 [0.2, 0.9] | 11.7 [10.0, 13.4] | 1.0 [0.5, 1.5] |
| 2020 | 303.3 [294.9, 311.7] | 1.9 [1.2, 2.5] | 8.2 [6.9, 9.6] | 12.1 [10.4, 13.8] | 2.0 [1.3, 2.7] | 1.9 [1.2, 2.5] | 0.7 [0.3, 1.1] | 11.8 [10.1, 13.4] | 0.9 [0.4, 1.4] |
| 2021 | 311.5 [303.1, 319.9] | 2.6 [1.9, 3.4] | 9.6 [8.2, 11.1] | 13.9 [12.1, 15.6] | 1.5 [0.9, 2.0] | 1.6 [1.0, 2.2] | 0.9 [0.4, 1.3] | 10.9 [9.4, 12.5] | 0.6 [0.2, 1.0] |
| 2022 | 343.0 [334.3, 351.7] | 3.3 [2.5, 4.2] | 10.5 [9.0, 12.0] | 18.3 [16.3, 20.3] | 1.9 [1.3, 2.6] | 2.2 [1.5, 2.9] | 1.1 [0.6, 1.6] | 12.3 [10.6, 13.9] | 1.2 [0.7, 1.7] |
| 2023 | 331.5 [323.2, 339.9] | 2.9 [2.1, 3.6] | 10.8 [9.3, 12.3] | 18.0 [16.1, 20.0] | 1.9 [1.3, 2.6] | 1.2 [0.7, 1.7] | 1.1 [0.6, 1.6] | 9.7 [8.2, 11.1] | 0.8 [0.4, 1.2] |

Table A9-4: Crude mortality rates (per 100 000 population) from all causes, all unintentional causes, other unintentional causes and undetermined causes, aged 80 years and older, Canada, from 2000 to 2023 (with 95% confidence intervals)

| Year | All causes | All unintentional causes | Other unintentional causes | Undetermined causes |
| --- | --- | --- | --- | --- |
| 2000 | 285.3 [274.3, 296.4] | 273.1 [262.3, 283.9] | 130.2 [122.7, 137.7] | 1.6 [0.7, 2.4] |
| 2001 | 271.3 [260.8, 281.8] | 262.0 [251.6, 272.3] | 115.2 [108.3, 122.0] | 1.0 [0.3, 1.6] |
| 2002 | 290.8 [280.2, 301.5] | 277.5 [267.1, 287.9] | 133.6 [126.4, 140.9] | 1.0 [0.4, 1.6] |
| 2003 | 295.8 [285.3, 306.3] | 281.9 [271.6, 292.2] | 131.4 [124.4, 138.4] | 1.8 [1.0, 2.7] |
| 2004 | 289.2 [279.0, 299.4] | 275.4 [265.4, 285.3] | 107.4 [101.2, 113.6] | 2.1 [1.3, 3.0] |
| 2005 | 283.8 [273.9, 293.7] | 271.9 [262.2, 281.6] | 110.2 [104.0, 116.4] | 1.3 [0.7, 2.0] |
| 2006 | 288.0 [278.3, 297.8] | 275.3 [265.7, 284.8] | 98.3 [92.6, 104.0] | 1.1 [0.5, 1.7] |
| 2007 | 286.8 [277.2, 296.4] | 273.6 [264.2, 283.0] | 86.6 [81.4, 91.9] | 1.5 [0.8, 2.2] |
| 2008 | 301.0 [291.3, 310.7] | 288.4 [278.9, 297.8] | 81.7 [76.7, 86.8] | 1.2 [0.6, 1.8] |
| 2009 | 315.1 [305.3, 324.9] | 301.9 [292.3, 311.4] | 67.1 [62.6, 71.6] | 0.9 [0.4, 1.4] |
| 2010 | 336.9 [326.9, 346.8] | 323.5 [313.7, 333.2] | 67.0 [62.6, 71.5] | 1.1 [0.6, 1.7] |
| 2011 | 338.3 [328.5, 348.1] | 325.1 [315.5, 334.7] | 65.3 [61.0, 69.6] | 1.1 [0.5, 1.7] |
| 2012 | 342.7 [333.0, 352.4] | 330.6 [321.1, 340.2] | 59.4 [55.3, 63.4] | 1.1 [0.5, 1.6] |
| 2013 | 345.4 [335.8, 355.1] | 329.5 [320.1, 338.9] | 58.3 [54.4, 62.3] | 0.8 [0.3, 1.2] |
| 2014 | 347.8 [338.2, 357.4] | 333.3 [324.0, 342.7] | 62.9 [58.8, 66.9] | 1.2 [0.7, 1.8] |
| 2015 | 340.4 [331.0, 349.9] | 325.8 [316.6, 335.0] | 61.8 [57.8, 65.8] | 1.1 [0.6, 1.7] |
| 2016 | 345.3 [335.9, 354.6] | 331.0 [321.8, 340.1] | 68.8 [64.6, 73.0] | 1.1 [0.6, 1.7] |
| 2017 | 355.1 [345.7, 364.5] | 340.7 [331.5, 349.9] | 67.2 [63.1, 71.3] | 1.0 [0.5, 1.5] |
| 2018 | 377.0 [367.4, 386.6] | 364.0 [354.6, 373.4] | 65.0 [61.1, 69.0] | 1.2 [0.7, 1.7] |
| 2019 | 401.6 [391.9, 411.4] | 387.6 [378.0, 397.2] | 66.8 [62.8, 70.8] | 1.3 [0.7, 1.9] |
| 2020 | 407.9 [398.2, 417.7] | 393.7 [384.1, 403.3] | 63.6 [59.7, 67.4] | 1.6 [1.0, 2.2] |
| 2021 | 427.9 [418.1, 437.8] | 415.5 [405.8, 425.2] | 73.9 [69.8, 78.0] | 0.9 [0.5, 1.4] |
| 2022 | 457.9 [447.9, 467.9] | 443.8 [434.0, 453.7] | 63.4 [59.6, 67.1] | 0.6 [0.2, 0.9] |
| 2023 | 442.6 [432.9, 452.3] | 430.8 [421.2, 440.3] | 63.3 [59.7, 67.0] | 1.4 [0.8, 1.9] |

Table A10-1: Number of deaths by sub-cause for falls, total population, Canada, from 2000 to 2023

| Year | slip/trip | same level, involving snow/ice/skates/skis/another person | other falls on the same level | involving wheelchair/furniture | on/from stairs | other falls from one level to another | unspecified falls |
| --- | --- | --- | --- | --- | --- | --- | --- |
| 2000 | 85 | 20 | 31 | 99 | 236 | 168 | 931 |
| 2001 | 110 | 21 | 91 | 134 | 260 | 156 | 962 |
| 2002 | 107 | 26 | 182 | 145 | 257 | 202 | 860 |
| 2003 | 134 | 35 | 264 | 129 | 256 | 175 | 996 |
| 2004 | 153 | 39 | 345 | 163 | 280 | 151 | 1 094 |
| 2005 | 167 | 46 | 409 | 155 | 286 | 165 | 1 078 |
| 2006 | 186 | 37 | 494 | 173 | 277 | 184 | 1 260 |
| 2007 | 172 | 37 | 512 | 209 | 303 | 175 | 1 332 |
| 2008 | 209 | 63 | 592 | 290 | 378 | 167 | 1 420 |
| 2009 | 262 | 51 | 727 | 313 | 388 | 179 | 1 721 |
| 2010 | 302 | 52 | 934 | 347 | 394 | 183 | 1 855 |
| 2011 | 342 | 56 | 851 | 342 | 423 | 184 | 2 078 |
| 2012 | 391 | 46 | 852 | 409 | 405 | 166 | 2 241 |
| 2013 | 367 | 59 | 926 | 378 | 413 | 167 | 2 399 |
| 2014 | 351 | 69 | 1 118 | 374 | 463 | 165 | 2 358 |
| 2015 | 286 | 55 | 1 075 | 345 | 465 | 164 | 2 387 |
| 2016 | 283 | 37 | 1 106 | 375 | 472 | 170 | 2 503 |
| 2017 | 243 | 40 | 1 317 | 359 | 425 | 162 | 2 686 |
| 2018 | 251 | 61 | 1 571 | 409 | 428 | 181 | 2 864 |
| 2019 | 304 | 79 | 1 929 | 446 | 439 | 178 | 2 965 |
| 2020 | 495 | 76 | 2 345 | 479 | 466 | 174 | 2 797 |
| 2021 | 918 | 63 | 2 374 | 481 | 554 | 186 | 2 623 |
| 2022 | 1 356 | 89 | 2 578 | 536 | 629 | 204 | 2 728 |
| 2023 | 1 063 | 64 | 3 050 | 547 | 578 | 237 | 2 506 |

Table A10-2: Crude mortality rates (per 100 000 population) by sub-cause for falls, total population, Canada, from 2000 to 2023 (with 95% confidence intervals)

| Year | slip/trip | same level, involving snow/ice/skates/skis/another person | other falls on the same level | involving wheelchair/furniture | on/from stairs | other falls from one level to another | unspecified falls |
| --- | --- | --- | --- | --- | --- | --- | --- |
| 2000 | 0.3 [0.2, 0.3] | 0.1 [0.0, 0.1] | 0.1 [0.1, 0.1] | 0.3 [0.3, 0.4] | 0.8 [0.7, 0.9] | 0.5 [0.5, 0.6] | 3.0 [2.8, 3.2] |
| 2001 | 0.4 [0.3, 0.4] | 0.1 [0.0, 0.1] | 0.3 [0.2, 0.4] | 0.4 [0.4, 0.5] | 0.8 [0.7, 0.9] | 0.5 [0.4, 0.6] | 3.1 [2.9, 3.3] |
| 2002 | 0.3 [0.3, 0.4] | 0.1 [0.1, 0.1] | 0.6 [0.5, 0.7] | 0.5 [0.4, 0.5] | 0.8 [0.7, 0.9] | 0.6 [0.6, 0.7] | 2.7 [2.6, 2.9] |
| 2003 | 0.4 [0.4, 0.5] | 0.1 [0.1, 0.1] | 0.8 [0.7, 0.9] | 0.4 [0.3, 0.5] | 0.8 [0.7, 0.9] | 0.6 [0.5, 0.6] | 3.1 [3.0, 3.3] |
| 2004 | 0.5 [0.4, 0.6] | 0.1 [0.1, 0.2] | 1.1 [1.0, 1.2] | 0.5 [0.4, 0.6] | 0.9 [0.8, 1.0] | 0.5 [0.4, 0.5] | 3.4 [3.2, 3.6] |
| 2005 | 0.5 [0.4, 0.6] | 0.1 [0.1, 0.2] | 1.3 [1.1, 1.4] | 0.5 [0.4, 0.6] | 0.9 [0.8, 1.0] | 0.5 [0.4, 0.6] | 3.3 [3.1, 3.5] |
| 2006 | 0.6 [0.5, 0.7] | 0.1 [0.1, 0.2] | 1.5 [1.4, 1.7] | 0.5 [0.5, 0.6] | 0.9 [0.8, 1.0] | 0.6 [0.5, 0.6] | 3.9 [3.7, 4.1] |
| 2007 | 0.5 [0.4, 0.6] | 0.1 [0.1, 0.1] | 1.6 [1.4, 1.7] | 0.6 [0.5, 0.7] | 0.9 [0.8, 1.0] | 0.5 [0.5, 0.6] | 4.1 [3.8, 4.3] |
| 2008 | 0.6 [0.5, 0.7] | 0.2 [0.1, 0.2] | 1.8 [1.6, 1.9] | 0.9 [0.8, 1.0] | 1.1 [1.0, 1.3] | 0.5 [0.4, 0.6] | 4.3 [4.0, 4.5] |
| 2009 | 0.8 [0.7, 0.9] | 0.2 [0.1, 0.2] | 2.2 [2.0, 2.3] | 0.9 [0.8, 1.0] | 1.2 [1.0, 1.3] | 0.5 [0.5, 0.6] | 5.1 [4.9, 5.4] |
| 2010 | 0.9 [0.8, 1.0] | 0.2 [0.1, 0.2] | 2.7 [2.6, 2.9] | 1.0 [0.9, 1.1] | 1.2 [1.0, 1.3] | 0.5 [0.5, 0.6] | 5.5 [5.2, 5.7] |
| 2011 | 1.0 [0.9, 1.1] | 0.2 [0.1, 0.2] | 2.5 [2.3, 2.6] | 1.0 [0.9, 1.1] | 1.2 [1.1, 1.3] | 0.5 [0.5, 0.6] | 6.1 [5.8, 6.3] |
| 2012 | 1.1 [1.0, 1.2] | 0.1 [0.1, 0.2] | 2.5 [2.3, 2.6] | 1.2 [1.1, 1.3] | 1.2 [1.1, 1.3] | 0.5 [0.4, 0.6] | 6.5 [6.2, 6.7] |
| 2013 | 1.0 [0.9, 1.2] | 0.2 [0.1, 0.2] | 2.6 [2.5, 2.8] | 1.1 [1.0, 1.2] | 1.2 [1.1, 1.3] | 0.5 [0.4, 0.5] | 6.8 [6.6, 7.1] |
| 2014 | 1.0 [0.9, 1.1] | 0.2 [0.1, 0.2] | 3.2 [3.0, 3.3] | 1.1 [0.9, 1.2] | 1.3 [1.2, 1.4] | 0.5 [0.4, 0.5] | 6.7 [6.4, 6.9] |
| 2015 | 0.8 [0.7, 0.9] | 0.2 [0.1, 0.2] | 3.0 [2.8, 3.2] | 1.0 [0.9, 1.1] | 1.3 [1.2, 1.4] | 0.5 [0.4, 0.5] | 6.7 [6.4, 7.0] |
| 2016 | 0.8 [0.7, 0.9] | 0.1 [0.1, 0.1] | 3.1 [2.9, 3.2] | 1.0 [0.9, 1.1] | 1.3 [1.2, 1.4] | 0.5 [0.4, 0.5] | 6.9 [6.7, 7.2] |
| 2017 | 0.7 [0.6, 0.7] | 0.1 [0.1, 0.1] | 3.6 [3.4, 3.8] | 1.0 [0.9, 1.1] | 1.2 [1.1, 1.3] | 0.5 [0.4, 0.5] | 7.3 [7.1, 7.6] |
| 2018 | 0.7 [0.6, 0.8] | 0.2 [0.1, 0.2] | 4.2 [4.0, 4.4] | 1.1 [1.0, 1.2] | 1.2 [1.0, 1.3] | 0.5 [0.4, 0.6] | 7.7 [7.4, 8.0] |
| 2019 | 0.8 [0.7, 0.9] | 0.2 [0.2, 0.3] | 5.1 [4.9, 5.4] | 1.2 [1.1, 1.3] | 1.2 [1.1, 1.3] | 0.5 [0.4, 0.5] | 7.9 [7.6, 8.2] |
| 2020 | 1.3 [1.2, 1.4] | 0.2 [0.2, 0.2] | 6.2 [5.9, 6.4] | 1.3 [1.1, 1.4] | 1.2 [1.1, 1.3] | 0.5 [0.4, 0.5] | 7.4 [7.1, 7.6] |
| 2021 | 2.4 [2.2, 2.6] | 0.2 [0.1, 0.2] | 6.2 [6.0, 6.5] | 1.3 [1.1, 1.4] | 1.4 [1.3, 1.6] | 0.5 [0.4, 0.6] | 6.9 [6.6, 7.1] |
| 2022 | 3.5 [3.3, 3.7] | 0.2 [0.2, 0.3] | 6.6 [6.4, 6.9] | 1.4 [1.3, 1.5] | 1.6 [1.5, 1.7] | 0.5 [0.5, 0.6] | 7.0 [6.7, 7.3] |
| 2023 | 2.7 [2.5, 2.8] | 0.2 [0.1, 0.2] | 7.6 [7.3, 7.9] | 1.4 [1.3, 1.5] | 1.4 [1.3, 1.6] | 0.6 [0.5, 0.7] | 6.3 [6.0, 6.5] |

Table A11-1: Number of deaths by sub-cause for poisonings, total population, Canada, from 2000 to 2023

| Year | psychoactive drugs | nonopioid pain/fever & autonomic drugs | other/unspecified drugs | alcohol | chemicals & gases |
| --- | --- | --- | --- | --- | --- |
| 2000 | 482 | 28 | 285 | 71 | 97 |
| 2001 | 505 | 31 | 275 | 90 | 72 |
| 2002 | 487 | 33 | 361 | 68 | 81 |
| 2003 | 480 | 25 | 253 | 59 | 76 |
| 2004 | 491 | 31 | 280 | 75 | 80 |
| 2005 | 591 | 17 | 322 | 59 | 66 |
| 2006 | 637 | 20 | 385 | 64 | 49 |
| 2007 | 708 | 28 | 410 | 153 | 93 |
| 2008 | 716 | 30 | 408 | 215 | 68 |
| 2009 | 760 | 31 | 453 | 202 | 63 |
| 2010 | 873 | 40 | 411 | 191 | 62 |
| 2011 | 914 | 31 | 508 | 197 | 51 |
| 2012 | 1 029 | 32 | 477 | 202 | 56 |
| 2013 | 985 | 34 | 549 | 201 | 72 |
| 2014 | 1 134 | 37 | 598 | 248 | 73 |
| 2015 | 1 266 | 46 | 864 | 228 | 65 |
| 2016 | 1 889 | 42 | 1 356 | 208 | 74 |
| 2017 | 2 680 | 48 | 1 833 | 218 | 86 |
| 2018 | 2 680 | 40 | 1 958 | 213 | 73 |
| 2019 | 2 309 | 32 | 1 860 | 219 | 70 |
| 2020 | 3 541 | 54 | 2 815 | 225 | 45 |
| 2021 | 3 293 | 47 | 4 042 | 227 | 81 |
| 2022 | 3 282 | 49 | 3 844 | 201 | 96 |
| 2023 | 2 545 | 59 | 4 598 | 181 | 92 |

Table A11-2: Crude mortality rates (per 100 000 population) by sub-cause for poisonings, total population, Canada, from 2000 to 2023 (with 95% confidence intervals)

| Year | psychoactive drugs | nonopioid pain/fever & autonomic drugs | other/unspecified drugs | alcohol | chemicals & gases |
| --- | --- | --- | --- | --- | --- |
| 2000 | 1.6 [1.4, 1.7] | 0.1 [0.1, 0.1] | 0.9 [0.8, 1.0] | 0.2 [0.2, 0.3] | 0.3 [0.3, 0.4] |
| 2001 | 1.6 [1.5, 1.8] | 0.1 [0.1, 0.1] | 0.9 [0.8, 1.0] | 0.3 [0.2, 0.4] | 0.2 [0.2, 0.3] |
| 2002 | 1.6 [1.4, 1.7] | 0.1 [0.1, 0.1] | 1.2 [1.0, 1.3] | 0.2 [0.2, 0.3] | 0.3 [0.2, 0.3] |
| 2003 | 1.5 [1.4, 1.7] | 0.1 [0.0, 0.1] | 0.8 [0.7, 0.9] | 0.2 [0.1, 0.2] | 0.2 [0.2, 0.3] |
| 2004 | 1.5 [1.4, 1.7] | 0.1 [0.1, 0.1] | 0.9 [0.8, 1.0] | 0.2 [0.2, 0.3] | 0.3 [0.2, 0.3] |
| 2005 | 1.8 [1.7, 2.0] | 0.1 [0.0, 0.1] | 1.0 [0.9, 1.1] | 0.2 [0.1, 0.2] | 0.2 [0.2, 0.3] |
| 2006 | 2.0 [1.8, 2.1] | 0.1 [0.0, 0.1] | 1.2 [1.1, 1.3] | 0.2 [0.1, 0.2] | 0.2 [0.1, 0.2] |
| 2007 | 2.2 [2.0, 2.3] | 0.1 [0.1, 0.1] | 1.2 [1.1, 1.4] | 0.5 [0.4, 0.5] | 0.3 [0.2, 0.3] |
| 2008 | 2.2 [2.0, 2.3] | 0.1 [0.1, 0.1] | 1.2 [1.1, 1.3] | 0.6 [0.6, 0.7] | 0.2 [0.2, 0.3] |
| 2009 | 2.3 [2.1, 2.4] | 0.1 [0.1, 0.1] | 1.3 [1.2, 1.5] | 0.6 [0.5, 0.7] | 0.2 [0.1, 0.2] |
| 2010 | 2.6 [2.4, 2.7] | 0.1 [0.1, 0.2] | 1.2 [1.1, 1.3] | 0.6 [0.5, 0.6] | 0.2 [0.1, 0.2] |
| 2011 | 2.7 [2.5, 2.8] | 0.1 [0.1, 0.1] | 1.5 [1.4, 1.6] | 0.6 [0.5, 0.7] | 0.1 [0.1, 0.2] |
| 2012 | 3.0 [2.8, 3.1] | 0.1 [0.1, 0.1] | 1.4 [1.3, 1.5] | 0.6 [0.5, 0.7] | 0.2 [0.1, 0.2] |
| 2013 | 2.8 [2.6, 3.0] | 0.1 [0.1, 0.1] | 1.6 [1.4, 1.7] | 0.6 [0.5, 0.7] | 0.2 [0.2, 0.3] |
| 2014 | 3.2 [3.0, 3.4] | 0.1 [0.1, 0.1] | 1.7 [1.6, 1.8] | 0.7 [0.6, 0.8] | 0.2 [0.2, 0.3] |
| 2015 | 3.5 [3.4, 3.7] | 0.1 [0.1, 0.2] | 2.4 [2.3, 2.6] | 0.6 [0.6, 0.7] | 0.2 [0.1, 0.2] |
| 2016 | 5.2 [5.0, 5.5] | 0.1 [0.1, 0.2] | 3.8 [3.6, 4.0] | 0.6 [0.5, 0.7] | 0.2 [0.2, 0.3] |
| 2017 | 7.3 [7.1, 7.6] | 0.1 [0.1, 0.2] | 5.0 [4.8, 5.2] | 0.6 [0.5, 0.7] | 0.2 [0.2, 0.3] |
| 2018 | 7.2 [7.0, 7.5] | 0.1 [0.1, 0.1] | 5.3 [5.0, 5.5] | 0.6 [0.5, 0.7] | 0.2 [0.2, 0.2] |
| 2019 | 6.1 [5.9, 6.4] | 0.1 [0.1, 0.1] | 4.9 [4.7, 5.2] | 0.6 [0.5, 0.7] | 0.2 [0.1, 0.2] |
| 2020 | 9.3 [9.0, 9.6] | 0.1 [0.1, 0.2] | 7.4 [7.1, 7.7] | 0.6 [0.5, 0.7] | 0.1 [0.1, 0.2] |
| 2021 | 8.6 [8.3, 8.9] | 0.1 [0.1, 0.2] | 10.6 [10.2, 10.9] | 0.6 [0.5, 0.7] | 0.2 [0.2, 0.3] |
| 2022 | 8.4 [8.1, 8.7] | 0.1 [0.1, 0.2] | 9.9 [9.6, 10.2] | 0.5 [0.4, 0.6] | 0.2 [0.2, 0.3] |
| 2023 | 6.4 [6.1, 6.6] | 0.1 [0.1, 0.2] | 11.5 [11.1, 11.8] | 0.5 [0.4, 0.5] | 0.2 [0.2, 0.3] |

Table A12-1: Number of deaths by sub-cause for suicide, total population, Canada, from 2000 to 2023

| Year | asphyxia | drugs | firearms | jumping | Other means/sequelae |
| --- | --- | --- | --- | --- | --- |
| 2000 | 1 555 | 506 | 694 | 183 | 705 |
| 2001 | 1 522 | 559 | 661 | 187 | 813 |
| 2002 | 1 601 | 537 | 649 | 194 | 744 |
| 2003 | 1 669 | 617 | 620 | 198 | 683 |
| 2004 | 1 593 | 639 | 571 | 161 | 665 |
| 2005 | 1 688 | 626 | 594 | 183 | 671 |
| 2006 | 1 540 | 593 | 593 | 205 | 637 |
| 2007 | 1 563 | 701 | 538 | 203 | 649 |
| 2008 | 1 684 | 683 | 519 | 200 | 632 |
| 2009 | 1 819 | 722 | 535 | 194 | 661 |
| 2010 | 1 818 | 734 | 592 | 223 | 608 |
| 2011 | 1 825 | 756 | 539 | 204 | 614 |
| 2012 | 1 876 | 702 | 559 | 232 | 628 |
| 2013 | 1 902 | 706 | 544 | 227 | 678 |
| 2014 | 2 035 | 721 | 590 | 241 | 705 |
| 2015 | 2 176 | 717 | 642 | 246 | 705 |
| 2016 | 2 130 | 727 | 625 | 250 | 718 |
| 2017 | 2 232 | 651 | 622 | 257 | 716 |
| 2018 | 2 414 | 632 | 607 | 260 | 682 |
| 2019 | 2 381 | 662 | 609 | 273 | 696 |

Table A12-2: Crude mortality rates (per 100 000 population) by sub-cause for suicide, total population, Canada, from 2000 to 2023 (with 95% confidence intervals)

| Year | asphyxia | drugs | firearms | jumping | Other means/sequelae |
| --- | --- | --- | --- | --- | --- |
| 2000 | 5.1 [4.8, 5.3] | 1.6 [1.5, 1.8] | 2.3 [2.1, 2.4] | 0.6 [0.5, 0.7] | 2.3 [2.1, 2.5] |
| 2001 | 4.9 [4.7, 5.2] | 1.8 [1.7, 2.0] | 2.1 [2.0, 2.3] | 0.6 [0.5, 0.7] | 2.6 [2.4, 2.8] |
| 2002 | 5.1 [4.9, 5.4] | 1.7 [1.6, 1.9] | 2.1 [1.9, 2.2] | 0.6 [0.5, 0.7] | 2.4 [2.2, 2.5] |
| 2003 | 5.3 [5.0, 5.5] | 1.9 [1.8, 2.1] | 2.0 [1.8, 2.1] | 0.6 [0.5, 0.7] | 2.2 [2.0, 2.3] |
| 2004 | 5.0 [4.7, 5.2] | 2.0 [1.8, 2.2] | 1.8 [1.6, 1.9] | 0.5 [0.4, 0.6] | 2.1 [1.9, 2.2] |
| 2005 | 5.2 [5.0, 5.5] | 1.9 [1.8, 2.1] | 1.8 [1.7, 2.0] | 0.6 [0.5, 0.6] | 2.1 [1.9, 2.2] |
| 2006 | 4.7 [4.5, 5.0] | 1.8 [1.7, 2.0] | 1.8 [1.7, 2.0] | 0.6 [0.5, 0.7] | 2.0 [1.8, 2.1] |
| 2007 | 4.8 [4.5, 5.0] | 2.1 [2.0, 2.3] | 1.6 [1.5, 1.8] | 0.6 [0.5, 0.7] | 2.0 [1.8, 2.1] |
| 2008 | 5.1 [4.8, 5.3] | 2.1 [1.9, 2.2] | 1.6 [1.4, 1.7] | 0.6 [0.5, 0.7] | 1.9 [1.8, 2.0] |
| 2009 | 5.4 [5.2, 5.7] | 2.1 [2.0, 2.3] | 1.6 [1.5, 1.7] | 0.6 [0.5, 0.7] | 2.0 [1.8, 2.1] |
| 2010 | 5.3 [5.1, 5.6] | 2.2 [2.0, 2.3] | 1.7 [1.6, 1.9] | 0.7 [0.6, 0.7] | 1.8 [1.6, 1.9] |
| 2011 | 5.3 [5.1, 5.6] | 2.2 [2.0, 2.4] | 1.6 [1.4, 1.7] | 0.6 [0.5, 0.7] | 1.8 [1.6, 1.9] |
| 2012 | 5.4 [5.2, 5.6] | 2.0 [1.9, 2.2] | 1.6 [1.5, 1.7] | 0.7 [0.6, 0.8] | 1.8 [1.7, 2.0] |
| 2013 | 5.4 [5.2, 5.7] | 2.0 [1.9, 2.2] | 1.6 [1.4, 1.7] | 0.6 [0.6, 0.7] | 1.9 [1.8, 2.1] |
| 2014 | 5.7 [5.5, 6.0] | 2.0 [1.9, 2.2] | 1.7 [1.5, 1.8] | 0.7 [0.6, 0.8] | 2.0 [1.8, 2.1] |
| 2015 | 6.1 [5.8, 6.4] | 2.0 [1.9, 2.2] | 1.8 [1.7, 1.9] | 0.7 [0.6, 0.8] | 2.0 [1.8, 2.1] |
| 2016 | 5.9 [5.6, 6.1] | 2.0 [1.9, 2.2] | 1.7 [1.6, 1.9] | 0.7 [0.6, 0.8] | 2.0 [1.8, 2.1] |
| 2017 | 6.1 [5.9, 6.4] | 1.8 [1.6, 1.9] | 1.7 [1.6, 1.8] | 0.7 [0.6, 0.8] | 2.0 [1.8, 2.1] |
| 2018 | 6.5 [6.3, 6.8] | 1.7 [1.6, 1.8] | 1.6 [1.5, 1.8] | 0.7 [0.6, 0.8] | 1.8 [1.7, 2.0] |
| 2019 | 6.3 [6.1, 6.6] | 1.8 [1.6, 1.9] | 1.6 [1.5, 1.7] | 0.7 [0.6, 0.8] | 1.9 [1.7, 2.0] |

Table A13-1: Number of deaths by sub-cause for land transport incidents, total population, Canada, from 2000 to 2023

| Year | motor vehicle traffic crashes | non motor vehicle traffic crashes |
| --- | --- | --- |
| 2000 | 1 909 | 1 090 |
| 2001 | 1 922 | 971 |
| 2002 | 2 097 | 963 |
| 2003 | 1 970 | 984 |
| 2004 | 1 990 | 911 |
| 2005 | 2 191 | 942 |
| 2006 | 2 132 | 936 |
| 2007 | 1 965 | 1 052 |
| 2008 | 1 786 | 926 |
| 2009 | 1 599 | 877 |
| 2010 | 1 671 | 878 |
| 2011 | 1 498 | 837 |
| 2012 | 1 532 | 883 |
| 2013 | 1 423 | 845 |
| 2014 | 1 352 | 770 |
| 2015 | 1 393 | 810 |
| 2016 | 1 438 | 813 |
| 2017 | 1 374 | 737 |
| 2018 | 1 359 | 832 |
| 2019 | 1 251 | 756 |
| 2020 | 1 183 | 727 |
| 2021 | 1 185 | 697 |
| 2022 | 1 240 | 855 |
| 2023 | 932 | 929 |

Table A13-2: Crude mortality rates (per 100 000 population) by sub-cause for land transport incidents, total population, Canada, from 2000 to 2023 (with 95% confidence intervals)

| Year | motor vehicle traffic crashes | non motor vehicle traffic crashes |
| --- | --- | --- |
| 2000 | 6.2 [5.9, 6.5] | 3.6 [3.3, 3.8] |
| 2001 | 6.2 [5.9, 6.5] | 3.1 [2.9, 3.3] |
| 2002 | 6.7 [6.4, 7.0] | 3.1 [2.9, 3.3] |
| 2003 | 6.2 [6.0, 6.5] | 3.1 [2.9, 3.3] |
| 2004 | 6.2 [6.0, 6.5] | 2.9 [2.7, 3.0] |
| 2005 | 6.8 [6.5, 7.1] | 2.9 [2.7, 3.1] |
| 2006 | 6.5 [6.3, 6.8] | 2.9 [2.7, 3.1] |
| 2007 | 6.0 [5.7, 6.2] | 3.2 [3.0, 3.4] |
| 2008 | 5.4 [5.1, 5.6] | 2.8 [2.6, 3.0] |
| 2009 | 4.8 [4.5, 5.0] | 2.6 [2.4, 2.8] |
| 2010 | 4.9 [4.7, 5.1] | 2.6 [2.4, 2.8] |
| 2011 | 4.4 [4.1, 4.6] | 2.4 [2.3, 2.6] |
| 2012 | 4.4 [4.2, 4.6] | 2.5 [2.4, 2.7] |
| 2013 | 4.1 [3.8, 4.3] | 2.4 [2.2, 2.6] |
| 2014 | 3.8 [3.6, 4.0]  4.4 [4.2, 4.6]  4.1 [3.8, 4.3]4.9 [4.7, 5.1] | 2.2 [2.0, 2.3] |
| 2015 | 3.9 [3.7, 4.1] | 2.3 [2.1, 2.4] |
| 2016 | 4.0 [3.8, 4.2] | 2.3 [2.1, 2.4] |
| 2017 | 3.8 [3.6, 4.0] | 2.0 [1.9, 2.2] |
| 2018 | 3.7 [3.5, 3.9] | 2.2 [2.1, 2.4] |
| 2019 | 3.3 [3.1, 3.5] | 2.0 [1.9, 2.2] |
| 2020 | 3.1 [2.9, 3.3] | 1.9 [1.8, 2.1] |
| 2021 | 3.1 [2.9, 3.3] | 1.8 [1.7, 2.0] |
| 2022 | 3.2 [3.0, 3.4] | 2.2 [2.0, 2.3] |
| 2023 | 2.3 [2.2, 2.5] | 2.3 [2.2, 2.5] |
