# Supplementary material for: Temporal trends in leading causes of injury deaths in Canada from 2000 to 2023: a time series analysis of mortality data
Source: Inj Epidemiol. 2026 Aug 6;13:61. doi: 10.1186/s40621-026-00702-4 (PMC13587287; doi:10.1186/s40621-026-00702-4)
Supplement: Supplementary file 2 [file 40621_2026_702_MOESM4_ESM.docx]

Table A16: The difference between this study and the Canadian Vital Statistics - Death database (CVSD) for suicide numbers in Canada from 2020 to 2023

| **Year** | **CVSD** | **This study** | **Difference *n (%)*** |
| --- | --- | --- | --- |
| 2020 | 4 283 | 4 523 | 240 (5.3%) |
| 2021 | 3 927 | 4 508 | 581 (12.9%) |
| 2022 | 4 218 | 4 982 | 764 (15.3%) |
| 2023 | 3 837 | 4 735 | 898 (19.0%) |

Table A17: Canadian age-standardized suicide rates (per 100 000 population) with a stratification based on a wider age interval (0–9, 10–14, 15–19, 20–24, 25–44, 45–64, 65–79, 80+) compared to a 5-year interval from 2000 to 2019

| **Year** | **5-year interval** | **Wider age interval** | **Absolute difference** | **Percent difference** |
| --- | --- | --- | --- | --- |
| 2000 | 11.88839 | 12.09776 | 0.20937 | 1.76% |
| 2001 | 12.06265 | 12.31349 | 0.25084 | 2.08% |
| 2002 | 11.94887 | 12.12612 | 0.17725 | 1.48% |
| 2003 | 12.00545 | 12.17226 | 0.16681 | 1.39% |
| 2004 | 11.33038 | 11.50279 | 0.17241 | 1.52% |
| 2005 | 11.49882 | 11.78125 | 0.28242 | 2.46% |
| 2006 | 10.97956 | 11.12459 | 0.14503 | 1.32% |
| 2007 | 10.95993 | 11.14860 | 0.18867 | 1.72% |
| 2008 | 11.07743 | 11.22004 | 0.14261 | 1.29% |
| 2009 | 11.54209 | 11.65720 | 0.11512 | 1.00% |
| 2010 | 11.55600 | 11.64665 | 0.09065 | 0.78% |
| 2011 | 11.34331 | 11.41918 | 0.07587 | 0.67% |
| 2012 | 11.36893 | 11.42814 | 0.05921 | 0.52% |
| 2013 | 11.46317 | 11.52789 | 0.06473 | 0.56% |
| 2014 | 11.99635 | 12.05848 | 0.06213 | 0.52% |
| 2015 | 12.47339 | 12.51352 | 0.04014 | 0.32% |
| 2016 | 12.26201 | 12.30009 | 0.03808 | 0.31% |
| 2017 | 12.19392 | 12.21784 | 0.02392 | 0.20% |
| 2018 | 12.34826 | 12.36536 | 0.01710 | 0.14% |
| 2019 | 12.26294 | 12.27042 | 0.00748 | 0.06% |
